# Theoretical Insights into the Resonant Suppression Effect in Vibrational Polariton Chemistry

**DOI:** 10.1021/jacs.5c03182

**Published:** 2025-06-02

**Authors:** Sebastian Montillo Vega, Wenxiang Ying, Pengfei Huo

**Affiliations:** † Department of Chemistry, 6927 University of Rochester, Rochester, New York 14627, United States; ‡ The Institute of Optics, Hajim School of Engineering, 6927 University of Rochester, Rochester, New York 14627, United States; § Center for Coherence and Quantum Science, 6927 University of Rochester, Rochester, New York 14627, United States

## Abstract

Recent experiments demonstrated the possibilities of modifying ground-state chemical reaction rates by placing an ensemble of molecules in an optical microcavity through a resonant coupling between the cavity mode and molecular vibrations. Typical VSC experiments operate in the absence of any light source. The VSC-induced rate constant suppression occurs under the resonance condition when the cavity frequency matches the molecular vibrational frequency, and only under the normal incidence when considering in-plane momentum inside a Fabry–Pérot cavity. In this work, we use quantum dynamics simulations and analytic theories to provide valuable insights into observed VSC phenomena, including the resonance behavior, the nonlinear change of the rate constant when increasing Rabi splitting, modification of both reactive enthalpy and entropy, and a reason why, with a very low barrier, there is a lack of the cavity modification. The analytic theory also exhibits the normal incidence condition and collective coupling effects.

## Graphical abstract

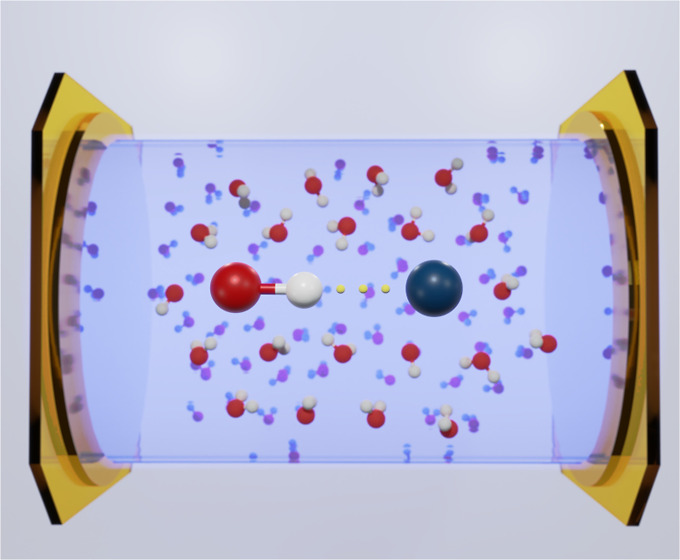

## Introduction

Recent experiments^1–10^ by Ebbesen, Simpkins, and others demonstrated that chemical reaction rate constants could be suppressed^1,7^ or enhanced^8–11^ by resonantly coupling molecular vibrations to quantized radiation modes inside a Fabry–Pérot (FP) microcavity.^12–14^ This effect, known as vibrational strong coupling (VSC) modified ground-state chemical reactivity, has the potential to selectively slow down competing reactions^2^ or speed up a target reaction, thus achieving mode selectivity^2,15^ and offering new chemical transformation strategies in synthetic chemistry.

There are several characteristic, universal phenomena in the VSC experiments,^12,13,16,17^ including (1) the resonance effect,^1,7^ where the maximum VSC effect occurs when the cavity frequency is tuned to the vibrational frequency ω_c_ = ω_Q_; (2) the normal incidence effect,^1^ where the VSC effect only happens when the in-plane photon momentum is *k*_∥_ = 0; (3) the collective effect^1,4,8^ where the magnitude of VSC modification increases when increasing the number of molecules *N*; (4) the reaction is under thermal activation without any optical pumping.^1,2^ To the best of our knowledge, there is no unified, microscopic theory that can explain all of the above-observed phenomena,^16–19^ despite many insightful hypothesis and mechanisms^7,15,20–42^ that have been proposed to explain the VSC effects. In particular, there is no analytic rate constant theory that could explain the sharp resonance suppression behavior. The transition state theory (TST) predicts no frequency-dependent VSC effects,^20,25^ and existing rate constant theory often depends on the barrier frequency, or has a very broad cavity-frequency dependence for the rate changes.^20,21,32,43^ These difficulties in using the existing rate theories to explain the sharp resonance VSC effects hint that a proper mechanistic description of the VSC resonance suppression might need a completely new analytic rate constant expression. Recently, several key theoretical advances^21,44^ have demonstrated that a sharp resonance enhancement of the rate constant occurs when using a quantum state description of the vibrational states, a finding verified by exact quantum dynamics simulations.^21,44–46^

In this work, we present a microscopic theory and mechanism that provide insights into understanding the characteristic features of the observed VSC effects mentioned above. We consider a theoretical model where a cavity mode couples to a set of solvent vibrations $\left\{Q_{j}\right\}$ (spectator modes, or rate-promoting vibrations) which in turn couple to a reaction coordinate *R*_0_. This model captures the key features of recent VSC experiments^1,7^ and has been employed in previous theoretical studies on VSC-modified reactivities.^21,31^ It also reflects the characteristics observed in theoretical simulations of polaritonic vibrational energy relaxation,^29^ which demonstrate collective and resonance behavior. We derive an analytic rate constant expression based on Fermi’s Golden Rule (FGR) and employ numerically exact quantum dynamics simulations to demonstrate the accuracy of the theory. The theory provides theoretical insights into the resonance VSC suppression of the rate constant under normal-incidence conditions, collective light-matter coupling, and the thermally activated regime.

## Methods

### Theoretical Model

We use the Pauli–Fierz (PF) Hamiltonian to describe the light-matter interaction of molecular vibrations in an optical cavity,^17,47^ expressed as follows

1 $$Ĥ={Ĥ}_{0}+{Ĥ}_{Q}+{Ĥ}_{LM}+{Ĥ}_{\nu }+{Ĥ}_{loss}$$

where ${Ĥ}_{0}={\mathrm{T̂}}_{0}+\mathrm{V̂}\left({\mathrm{R̂}}_{0}\right)$ is the Hamiltonian of the reactive mode, with the reaction coordinate ${\mathrm{R̂}}_{0}$ , and ground state potential energy surface as a symmetric double well potential along *R*_0_. We use a set of localized diabatic vibrational states, |ν_L_⟩ and |ν_R_⟩ to represent ground vibrational states associated with the left well (reactant) and the right well (product), as well as |ν_L_^′^⟩ and |ν_R_^′^⟩ associated with the vibrational excited states, depicted in Figure 1d. In this picture, the vibrational frequency ω_0_ for the reactant is defined as the transition frequency associated with |ν_L_⟩ → |ν_L_^′^⟩, that is ω_0_ ≡ (*E*_ν_L_^′^_ – *E*_νL_)/ℏ. The details of the vibrational states and the description of ${Ĥ}_{\nu }$ and ${Ĥ}_{loss}$ are provided in Supporting Information, Section 1.

**Figure 1:**
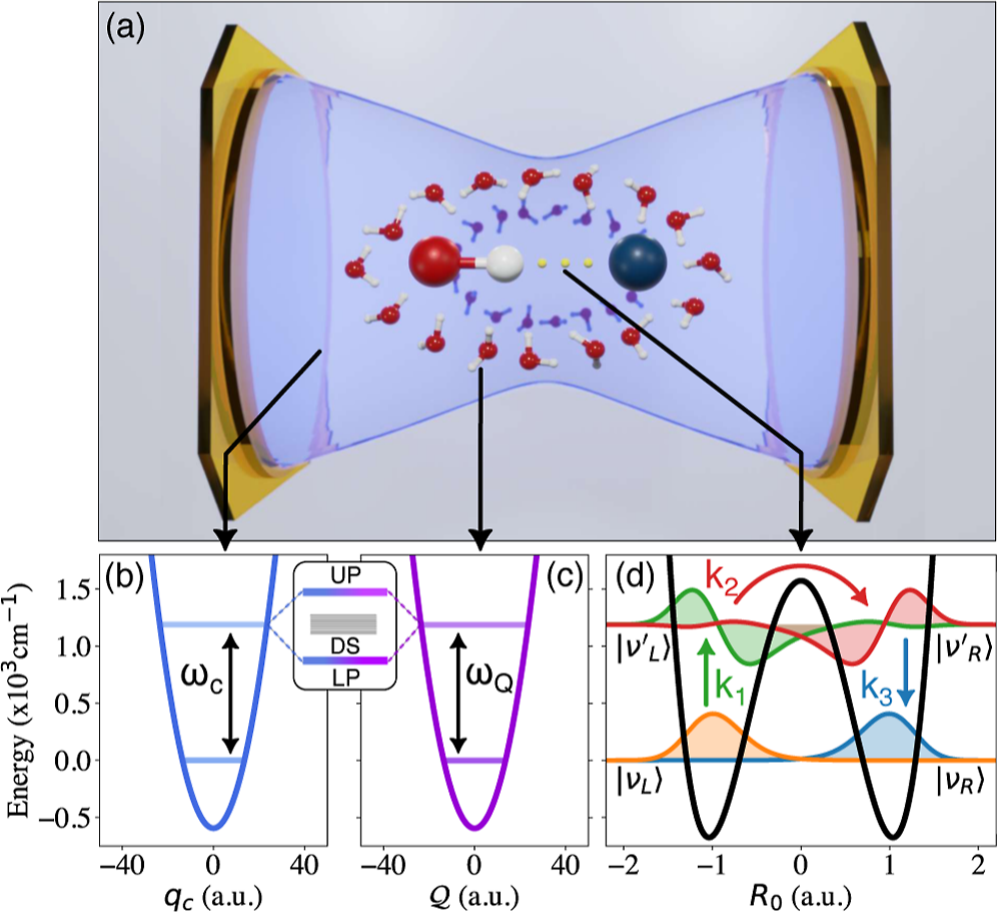
Schematic illustration of the VSC-modified reactions and the mechanisms. (a) Schematic illustration of a reactive molecule whose reaction coordinate is coupled to a set of solvent vibrations, which are in turn coupled to a cavity mode. (b) Photonic potential coupled to (c) an intermolecular vibration whose hybridization leads to a set of polaritonic states with Rabi splitting Ω_R_. The intermolecular vibration, in turn, couples to a reaction coordinate *R*_0_. (d) Potential energy surface of the chemical reaction along the reaction coordinate, with four key vibrational states visualized.

We further consider that the reaction coordinate ${\mathrm{R̂}}_{0}$ is coupled to a set of nonreactive spectator modes $\left\{Q_{j}\right\}$ . We model the interactions between Q modes and *R*_0_ using the Hamiltonian^21^

2 $${Ĥ}_{Q}=\sum_{j=1}^{N}\frac{{\mathrm{\Pi ̂}}_{j}^{2}}{2}+\frac{\omega_{Q}^{2}}{2}{\left({\mathrm{Q̂}}_{j}-\frac{C_{j}}{\omega_{Q}^{2}}{\mathrm{R̂}}_{0}\right)}^{2}$$

where ${\mathrm{\Pi ̂}}_{j}$ and ${\mathrm{Q̂}}_{j}$ are the momentum and coordinate for the Q modes, with coupling strength $C_{j}$ . These modes have a uniform frequency ω_Q_. This model captures essential features of several VSC reactions. For example, in ref 1, the C–Si is the *R*_0_ reaction coordinate, and Si–(Me)_3_ is nonreactive Q mode that strongly couple to *R*_0_ (for *N* = 1). In ref 7, the spectator mode Q could be the N–C–O vibrational mode in the reactant (for *N* = 1). The solvent vibrations could also be viewed as the spectator mode $\left\{Q_{j}\right\}$ (for *N* ≫ 1), which was studied in the recent VSC theoretical investigations.^21,31^

We define the reorganization energy for *R*_0_ caused by $Q_{j}$ coupling as

3 $$\Lambda =\sum_{j=1}^{N}C_{j}^{2}/\left(2\omega_{Q}^{2}\right)$$

Note that Λ is a collective quantity between the reaction coordinate *R*_0_ and the solvent DOF $Q_{j}$ . For the solvent–solute interactions in eq 2, with an increasing number of solvent molecules, the outer sphere solvents will only be weakly coupled to the solute, while the solvent inner sphere will be strongly coupled, resulting in a solute–solvent distance-specific coupling strength $C_{j}$ . In this study, we keep Λ as a fixed parameter.

The light-matter interaction Hamiltonian ${Ĥ}_{LM}$ considers one cavity mode coupled to the $\left\{Q_{j}\right\}$ modes, expressed as

4 $${Ĥ}_{LM}=\frac{{\mathrm{p̂}}_{c}^{2}}{2}+\frac{\omega_{c}^{2}}{2}{\left({\mathrm{q̂}}_{c}+\sqrt{\frac{2}{\omega_{c}}}\eta_{c}\sum_{j=1}^{N}{\mathrm{Q̂}}_{j}\cdot \cos\varphi_{j}\right)}^{2}$$

where the photonic position operator ${\mathrm{q̂}}_{c}=\sqrt{\hbar /\left(2\omega_{c}\right)}\left({â}^{†}+â\right)$ and momentum operator ${\mathrm{p̂}}_{c}=i\sqrt{\hbar \omega_{c}/2}\left({â}^{†}-â\right)$ are expressed in terms of the photonic creation operator ${â}^{†}$ and annihilation â for a given cavity mode, with ω_c_ as the cavity frequency. A generalization of this term with many cavity modes will be discussed at the end of the paper. Further, $\eta_{c}=\sqrt{1/\left(2\hbar \omega_{c}\epsilon_{0}V\right)}$ is the light-matter coupling strength, where ϵ_0_ is the permittivity inside the cavity, and V is the effective quantization volume of that mode. For simplicity, we have assumed that the dipole operators are linear^21,27^ such that $\mathrm{\mu ̂}\left(Q_{j}\right)\cdot {ê}_{⊥}\approx {\mathrm{Q̂}}_{j}\cdot \cos\varphi_{j}$ , where ${ê}_{⊥}$ is the transverse field polarization direction (one of the two inplane directions in a FP cavity^17,46^ ). In contrast to the $R_{0}-Q_{j}$ interaction characterized by $C_{j}$ (electrostatic interactions) which depends on the solute–solvent distance, the cavity mode, on the other hand, interacts with all solvent modes $\left\{Q_{j}\right\}$ with η_c_ weighted by cos φ_*j*_, resulting in delocalized interactions that can be observed from the Rabi splitting from spectroscopy.^48^

Under the resonance condition of ω_c_ = ω_Q_, the total Rabi splitting from the spectral measurements is related to the light-matter coupling strength. When there is no dipole angle disorder (cos φ_*j*_ = 1 for all *j*), the Rabi splitting is expressed as^16,17,48,49^

5 $$\Omega_{R}=2\sqrt{N}\eta_{c}\omega_{c}\mu_{Q}$$

the transition dipole matrix element associated with $Q_{j}$ is expressed as $\mu_{Q}=\left\langle 0\left|{\mathrm{Q̂}}_{j}\right|1\right\rangle$ (for linear dipole approximation^20^) which is assumed to be identical for all $Q_{j}$ modes.

Based on this model and the effective spectral density theory, we show how the cavity mode collectively coupled to the spectator modes influences the forward reaction along a reaction coordinate *R*_0_. We then derive an analytical rate constant using Fermi’s Golden Rule (FGR), the accuracy of which is confirmed by numerically exact quantum dynamics simulations via the Hierarchical equations of motion (HEOM) approach.

## Results and Discussions

### Mechanism and Analytic Rate Constant Expression

Under the thermally activated initial condition, the reaction coordinate *R*_0_ undergoes a barrier-crossing process, where the transition state is reached and finally relaxes to the product configuration. Quantum mechanically, the same process is described as (1) the thermally activated vibrational excitation on the reactant side |ν_L_⟩ → |ν_L_^′^⟩, (2) vibrational excited states transition $|\nu_{L}^{\prime }\rangle \to |\nu_{R}^{\prime }\rangle$ , and (3) vibrational relaxation on the product side |ν_R_^′^⟩ → |ν_R_⟩. The population dynamics from the HEOM exact simulations (see Supporting Information 4) indicate that the reaction mechanism can be described as follows^45^

6 $$|\nu_{L}\rangle \overset{k_{1}}{\to }|\nu_{L}^{\prime }\rangle \overset{k_{2}}{\to }|\nu_{R}^{\prime }\rangle \overset{k_{3}}{\to }|\nu_{R}\rangle$$

where the initial vibrational excitation (|ν_L_⟩ → |ν_L_^′^⟩) is the rate-limiting step, such that *k*_1_ ≪ *k*_2_, *k*_3_, see Figure 1d. This leads to a steady state population (time-independent plateau population) of the intermediate states |ν_L_^′^⟩ and |ν_R_^′^⟩, and the overall rate constant for the entire reaction (*k*) can be approximate as *k* ≈ *k*_1_. The |ν_L_⟩ → |ν_L_^′^⟩ transition is influenced by the energy exchange between the phonon bath and the spectator modes $\left\{Q_{j}\right\}$ . When these spectator modes are resonantly coupled to the cavity, the light-matter hybrid system has a set of polaritonic modes with frequencies ω_±_ = ω_Q_ ± Ω_R_/2, thus effectively removing the influence of Q from the |ν_L_⟩ → |ν_L_^′^⟩ transition. Another way to understand such influence is the strong coupling between $\left\{Q_{j}\right\}$ and ${\mathrm{q̂}}_{c}$ causes a fast exchange of energy among them (with frequency Ω_R_), and thus effectively decoupled from the |ν_L_⟩ → |ν_L_^′^⟩ transition. This will explicitly decrease the value of *k*_1_ and, as |ν_L_⟩ → |ν_L_^′^⟩ is still the rate-limiting step for the system compiled inside the cavity (see Supporting Information 4), the influence of cavity on *k*_1_ explicitly shows up in the entire apparent rate constant of the reaction because *k* ≈ *k*_1_.

Based on this observation, the impact of VSC on the rate is solely attributed to the cavity interacting with the spectator modes, and influencing the rate of the |ν_L_⟩ → |ν_L_^′^⟩ transition, hence imprint its impact on the apparent rate constant of the reaction. This is confirmed by the exact quantum dynamics simulations presented in Figure S2 (Supporting Information 4), where the steady-state population of the |ν_L_^′^⟩ is suppressed when coupling to the cavity. To develop an analytic theory, we expressed the overall rate constant as *k* ≈ *k*_1_ = *k*_D_ + α·*k*_VSC_, where *k*_D_ is the rate constant for the double-well potential without coupling to any spectator modes $Q_{j}$ or the cavity mode *q*_c_, and *k*_VSC_ the rate provided by the spectator modes with α as a scaling parameter. When FGR is exact and the role of $\left\{Q_{j}\right\}$ and *q*_c_ are directly additive to *k*, α = 1. Throughout the paper, we report the ratio of the rate constant inside and outside the cavity as follows

7 $$k/k_{0}=k_{D}/k_{0}+\alpha \cdot k_{VSC}/k_{0}$$

where *k*_0_ and *k*_D_ are directly obtained from HEOM simulations as they are not related to the coupling of the cavity mode. In this work, under the condition η_c_ = 0 (outside the cavity), we find that α ≈ 0.7 will bring the FGR analytic results to quantitatively agree with the numerically exact simulations. This parameter is then fixed for all cases of η_c_ when coupling the Q mode to the cavity. Note *k*_VSC_ is the contribution of the spectator modes to the overall rate, thus when η_c_ = 0, *k*_VSC_/*k*_0_ has the largest contribution as the Q modes are in resonance with the |ν_L_⟩ → |ν_L_^′^⟩ transition, promoting the vibrational excitation along *R*_0_. When gradually increasing the light-matter coupling strength, *k*_VSC_/*k*_0_ will decrease (see eq 11), and eventually goes to zero, such that the *Q* modes will no longer promote the rate, and *k* ∼ *k*_D_. In this sense, one can say the resonance VSC between cavity and $\left\{Q_{j}\right\}$ modes includes a “polaron decoupling” ^50–52^ between $\left\{Q_{j}\right\}$ and *R*_0_, slowing down the reaction along *R*_0_.

We aim to develop an analytic rate constant theory that describes the role of $\left\{Q_{j}\right\}$ and ${\mathrm{q̂}}_{c}$ on the rate constant. To this end, we derived an effective spectral density, *J*_eff_(ω), that describes the coupling of ${Ĥ}_{Q}+{Ĥ}_{LM}+{Ĥ}_{loss}$ to the reaction coordinate *R*_0_, based on the effective spectral density theory.^53,54^ The derivation is provided in Supplementary Note 5, and the general expression is presented in eq S55. A more insightful, approximate form is

8 $$J_{eff}\left(\omega \right)=\frac{\Lambda \cdot \omega_{Q}^{2}\cdot \omega \Gamma_{Q}\left(\omega \right)}{{\left[\omega_{Q}^{2}-\omega^{2}+\mathrm{R̃}\left(\omega \right)\right]}^{2}+{\left[\omega \Gamma_{Q}\left(\omega \right)\right]}^{2}}$$

where Λ (defined in eq 3) characterizes the couplings between *N* spectator Q modes (e.g., solvent DOF) with *R*_0_, and ω_Q_ is the solvent frequency which we assume to be identical for all $\left\{{\mathrm{Q̂}}_{j}\right\}$ . In addition, Γ_Q_ characterizes the excitation decay rate in the $\left\{Q_{j}\right\}$ and cavity modes

$$\Gamma_{Q}\left(\omega \right)=\frac{2\lambda_{Q}}{\gamma_{Q}}+\frac{2N\chi^{2}\cdot \omega_{c}^{3}\eta_{c}^{2}\tau_{c}^{-1}}{{\left(\omega_{c}^{2}-\omega^{2}\right)}^{2}+\omega^{2}\tau_{c}^{-2}}$$

where λ_Q_ and γ_Q_ are phonon bath parameters for $\left\{Q_{j}\right\}$ modes (see eq S13 in Supporting Information 1), τ_c_ is the cavity lifetime, and ω_c_ is the cavity frequency. Further, χ characterizes the angle of the vibrational dipole operator relative to the cavity mode ${\mathrm{q̂}}_{c}$ , with the following expression

9 $$\chi =\frac{1}{N}\sum_{j}\cos\varphi_{j}\equiv \left\langle \cos\varphi \right\rangle$$

Finally, R̃(ω) in eq 8 is expressed as

$$\mathrm{R̃}\left(\omega \right)=\frac{2N\chi^{2}\cdot \omega_{c}\eta_{c}^{2}\omega^{2}}{{\left(\omega_{c}^{2}-\omega^{2}\right)}^{2}+\omega^{2}\tau_{c}^{-2}}\cdot \left(\omega^{2}-\omega_{c}^{2}+\tau_{c}^{-2}\right)$$

Note that in this approximate form of *J*_eff_(ω) in eq 8, there are *N* – 1 dark states that do not appear, thus being decoupled^31^ from the reaction coordinate ${\mathrm{R̂}}_{0}$ . This is due to a mean-field-like approximation for the $C_{j}$ when deriving eq 8. In the general expression of the spectral density (eq S55), the dark states can still show up (see Figure S5 and S6 in Supporting Information 5) when having disorders. This will be discussed in the Collective coupling mechanism section.

Figure 2a presents *J*_eff_(ω) at various Ω_R_. At Ω_R_ = 0 (black curve), the peak of *J*_eff_(ω) is located at the frequency of ω_Q_, and the spectator mode effect is at its maximum due to ω_Q_ = ω_0_. Note that this frequency matching between solvent vibration ω_Q_ and ω_0_ has been observed in experiments^8–11^ and was referred to as the cooperative coupling,^8^ although the VSC kinetics effect is rate constant enhancement in those studies.^8–11^ In ref 7, the solvent C–H vibration is strongly coupled to the cavity mode, which could also impact the rate constant and leads to a rate constant suppression effect, although the solvent frequency ω_Q_ seems to be largely detuned from the frequency of ω_0_, likely due to the anharmonic interaction effects^55^ which are not included by the bilinear interaction term in eq 2. As Ω_R_ increases, *J*_eff_(ω) split into two peaks (corresponding to the two vibrational polariton frequencies ω_±_ ≈ ω_Q_ ± Ω_R_/2), and is moving away from ω_0_, hence reducing the spectator mode effect and the value of *k*_1_ for |ν_L_⟩ → |ν_L_^′^⟩ transition (cf. eq 6). Note that the *J*_eff_(ω) expression (eq 8) is only sensitive to the collective coupling strength Ω_R_, and for *N* > 1, the behavior of *J*_eff_(ω) is identical to *N* = 1 as long as Ω_R_ and Λ are identical. The rest of the dark vibrational modes are decoupled from ${\mathrm{R̂}}_{0}$ and will not explicitly show up in *J*_eff_(ω).

**Figure 2:**
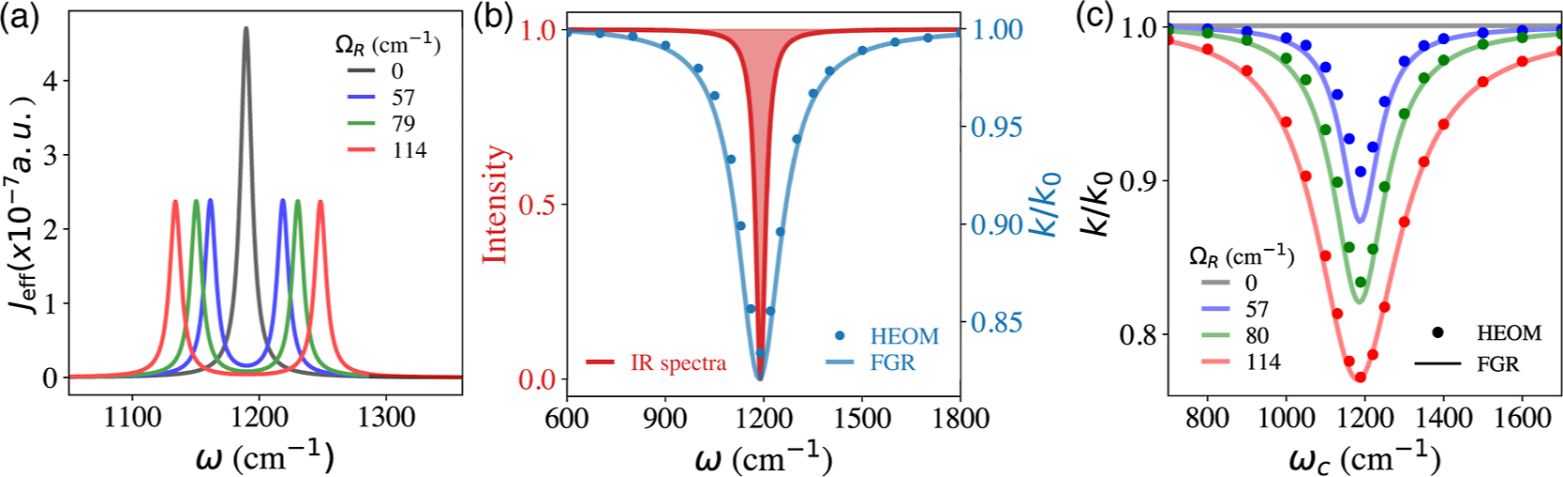
Resonance behavior of the VSC effect. (a) Effective spectral density *J*_eff_(ω) (eq 8) with different values of Ω_R_, where the two peaks correspond to the polariton modes. (b) VSC enabled rate constant reduction (blue) and the IR spectra (red) for the spectator mode $Q_{j}$ outside the cavity, both of which occur at ω_c_ = ω_Q_. The ratio of inside and outside the cavity rate constant *k*/*k*_0_ obtained from analytic FGR theory (blue solid line) and HEOM (blue solid circles) for *N* = 1 with Ω_R_ = 57 cm^–1^. (c) Normalized rate constant profile *k*/*k*_0_ (with respect to the outside cavity case *k*_0_) as a function of cavity frequency ω_c_ and various Ω_R_. All calculations are done with a cavity lifetime τ_c_ = 500 fs. The FGR results (solid lines) are rescaled by a factor of α = 0.7 (see eq 7).

To evaluate the |ν_L_⟩ → |ν_L_^′^⟩ transition rate constant *k*_VSC_ (eq 7), we use the FGR theory expressed as follows

10 kVSC=2|Δx|2∫0∞dωΛωQ2·ωΓQ(ω)·A0(ω−ω0)·e−βℏω0[ωQ2−ω2+R̃(ω)]2+[ωΓQ(ω)]2,

where ω is the transition frequency, ω_0_ is the vibrational frequency for the reactive mode *R*_0_, $A_{0}$ is the broadening function that accounts for the fluctuations in ω_0_ (see details in Supporting Information 1), $\Delta_{x}=\left\langle \nu_{L}\left|{\mathrm{R̂}}_{0}\right|\nu_{L}^{\prime }\right\rangle$ the vibrational transition dipole for the |ν_L_⟩ → |ν_L_^′^⟩ transition, β ≡ 1/(*k*_B_ *T*), and *k*_B_ is the Boltzmann constant.

The *k*_VSC_ expression in eq 10 is the first key result of this work. Note that the validity of FGR is under the condition $\sqrt{\Lambda \omega_{Q}}\cdot \left|\Delta_{x}\right|\ll k_{B}T\approx 200$ cm^–1^ under *T* = 300 K, and the “strong coupling condition” $\left(\Omega_{R}\gg \frac{1}{2}\tau_{c}^{-1}+\lambda_{Q}/\gamma_{Q}\right)$ will not break the validity of FGR. In this work, the model system has $\sum_{j}^{N}C_{j}\cdot \left|\Delta_{x}\right|/\sqrt{2\omega_{Q}}\approx 10$ cm^–1^, which was used in ref 21. Further simplifications of eq 8 under the resonance condition ω_c_ = ω_Q_ (see Supporting Information 6) indicate a scaling relation

11 $$k_{VSC}\propto \frac{1}{\Omega_{R}^{2}}\propto \frac{1}{N\eta_{c}^{2}}$$

both the HEOM numerical data (Figure 3a) and the experimental results (e.g., Figure 3D in ref 4) can be fitted well with *k*/*k*_0_ = *k*_D_/*k*_0_ + *a*/(1 + *b*·Ω_R_^2^) (with *a*, *b* as fitting parameters), details are provided in Supporting Information 6. This scaling relation is the second key result of this work.

**Figure 3:**
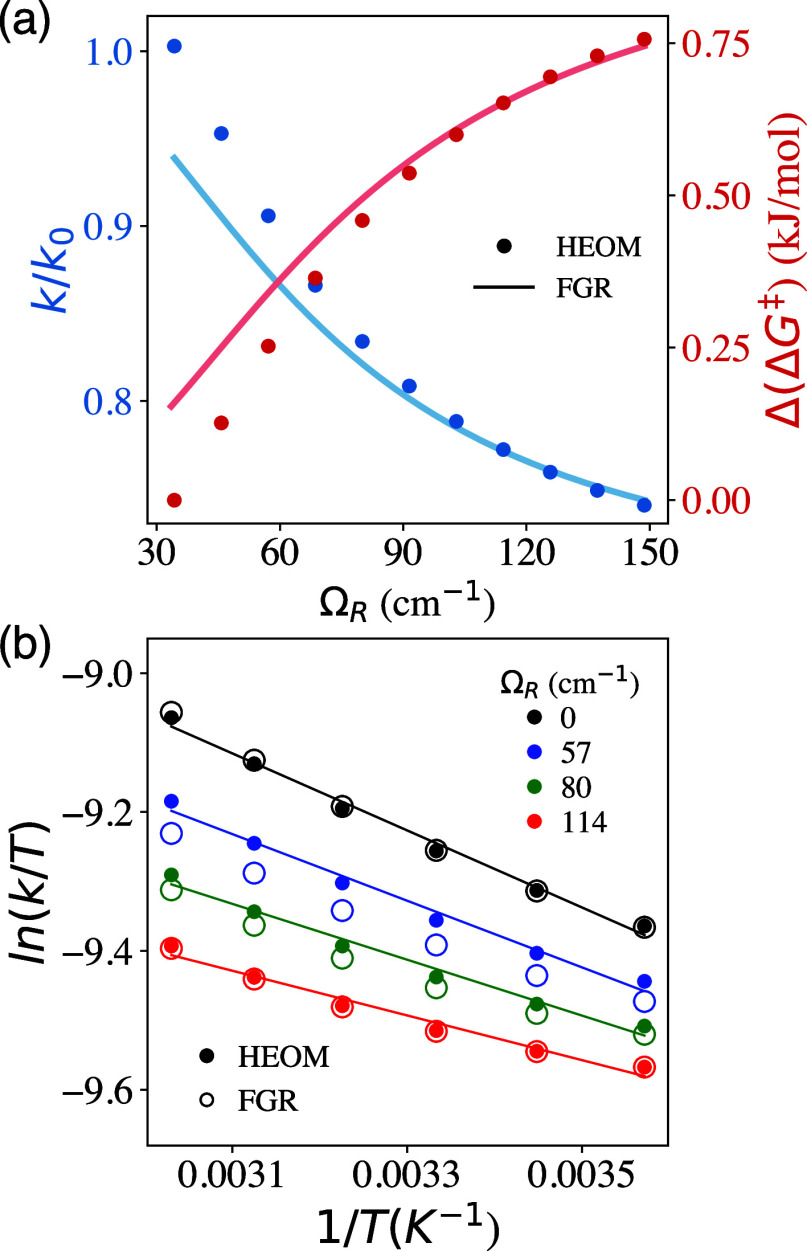
VSC modification of the rate constant predicted by FGR theory (eq 10). (a) Ratio of the rate constant inside and outside the cavity, *k*/*k*_0_, and effective free energy barrier change Δ­(Δ*G* ^⧧^) at different Ω_R_. (b) Eyring plot of ln­(*k*/*T*) vs 1/*T* at various Ω_R_. In all cases, the cavity is under the resonance condition ω_c_ = ω_Q_.

### VSC Rate Constant Modifications

To validate the *k*_VSC_ theory in eq 10, we perform numerical simulations of the rate constants for a single spectator mode Q(N=1) coupled to a single cavity mode, details are provided in Supporting Information 1. For *N* = 1, the Q mode could be either an intermolecular vibration or a nearby solvent that has a frequency ω_Q_ = ω_0_. Further, we set χ = 1 that corresponds to a vibrational dipole aligned to the cavity mode. All the simulations are performed at *T* = 300 K and a cavity lifetime τ_c_ = 500 fs, which are the typical values for the VSC experiments.^2,7^ The exact quantum dynamics propagation is done by using the HEOM approach.^56–58^ The details of the rate constant calculations are provided in Supporting Information 3.

Figure 2b presents the infrared (IR) profile of the spectator mode (red curve), alongside the cavity-frequency-dependent rate constant profile (blue curve) obtained from the HEOM simulations (blue dots) and the FGR approach (blue solid line, using eq 7). Both profiles exhibit a distinct sharp peak around ω_Q_, which is an essential feature observed in most VSC experiments.^1,7^ In particular, existing theories for resonance suppression often have a much broader peak^20,43^ for the rate constant profile (as a function of cavity frequency), which is significantly broader than the width of the line shape, as in those rates theories,^20,43^ the rate constant is not sensitive to ω_Q_ but rather the partition function expressions (which are influenced by multiple frequencies), resulting in a significantly broader frequency dependence.^45,59^ The new theory in eq 10 is capable of describing a sharp resonance suppression behavior. Reference 21 further demonstrates that for both energy diffusion-limited (see Figure 4 in ref 21) and spatial diffusion-limited regimes (see Figure S14 in ref 21), resonantly coupled cavity mode to Q will lead to a resonant suppression of the rate constant. The parameters used in our work are under the energy diffusion-limited regime. Meanwhile, ref 21 also demonstrates the resonance enhancement of VSC rate constant when coupling the cavity mode to the |ν_L_⟩ → |ν_L_^′^⟩ transition, which can also be explained by the FGR level of analytic theory in our previous work.^44^

**Figure 4:**
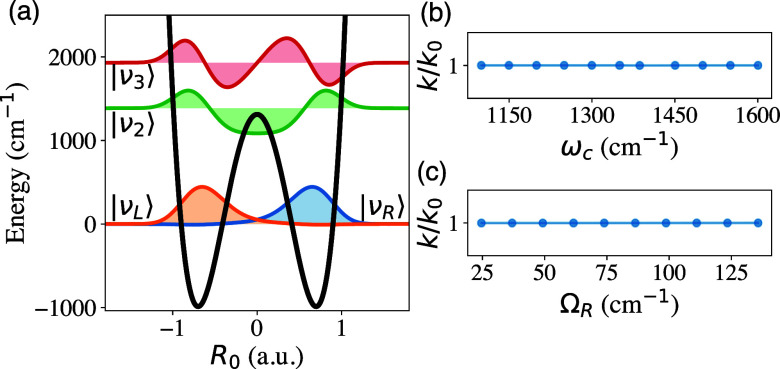
Null results of the VSC effects^60,61^ for a reaction that has a low energy barrier. (a) Reaction Potential along the reaction coordinate *R*_0_, with the low reaction barrier^60,61^ such that the vibrational excited states (green and red) are above the barrier, and the mechanistic steps in eq 6 are no longer satisfied. *k*/*k*_0_ at various (b) cavity frequencies and (c) Ω_R_, all showing null VSC reactivities.

Figure 2c presents the cavity frequency dependence of *k*/*k*_0_. Here, the maximum suppression is achieved when ω_c_ = ω_Q_. The VSC suppression effects are enlarged at a larger Ω_R_, because the peaks in *J*_eff_(ω) are farther away from the |ν_L_⟩ → |ν_L_^′^⟩ transition (as can be seen from Figure 2a). This increase in Ω_R_ also allows for light-matter interactions at larger cavity detuning, contributing to the broader rate profile. Our analytic FGR expression (eq 10) accurately captures the cavity frequency dependence of the rate constants for a wide range of Ω_R_. As such, *k*_VSC_ in eq 10 has achieved a quantitative agreement with the HEOM simulation results and is capable of describing the sharp resonance behavior observed in the experiments.^1,7^ We want to emphasize that the proposed VSC mechanism (eq 6), the shape resonance behavior of *k*/*k*_0_ as a function of ω_c_, as well as the analytic rate constant in eq 10 are robust against the detailed shape of the reactive potential. In Supporting Information 4C, we investigated the model reaction with an asymmetrical double-well potential and verified the validity of the mechanism as well as the sharp resonance behavior of *k*/*k*_0_, in good agreement with the analytical FGR theory.

Figure 3a presents *k*/*k*_0_ (blue dots and curve) as a function of Ω_R_ in the range in which the strong coupling condition is achieved. The results are obtained from HEOM simulations (blue dots), as well as from the FGR analytic expression (blue curve) based on eqs 7 and 10. The results clearly show a nonlinear relation of the rate constant with the Rabi splitting. In particular, at a large Rabi splitting, the maximum suppression of *k*/*k*_0_ converges to the value of the bare double rate (*k* → *k*_D_). As shown in Figure 2a, the cavity ultimately removes the effects the spectator mode has on the reaction rate by splitting the spectral density and shifting it away from the frequency of ω_0_. The trend of *k*/*k*_0_ closely resembles the experimental trend (e.g., Figure 3D in ref 4), and the FGR analytic expression closely agrees with the HEOM results, especially for a large value of Ω_R_. The fundamental scaling in eq 11 is also observed in our HEOM results, with details provided in Supporting Information 6. Note that the validity of FGR improves as Ω_R_ increases (FGR results get closer to HEOM). The reason for this “counterintuitive” result is that, in the current theory, Ω_R_ is not the perturbative parameter. The approximation in eq 10 comes from the perturbative treatment of the $Q-q_{c}$ DOF coupled to the |ν_L_⟩→|ν_L_^′^⟩ transition in the *R*_0_ DOF. As Ω_R_ increases, the $Q-q_{c}$ subsystem is further decoupled from the |ν_L_⟩ → |ν_L_^′^⟩ transition, making FGR more accurate.

Figure 3a also presents the change of the effective free energy barrier Δ­(Δ*G* ^⧧^) (red), directly calculated from the rate constant ratio *k*/*k*_0_ obtained from HEOM simulations (red dots) and FGR (red line). One can interpret the rate constant changes as the change of the effective free energy barrier Δ­(Δ*G* ^⧧^) through^4,8,45^ Δ­(Δ*G* ^⧧^) = Δ*G* ^⧧^ – Δ*G*_0_^‡^ = −*k*_B_ *T* ln­(*k*/*k*_0_). Note that this is not an actual change in the free-energy barrier, but rather an effective measure of the purely kinetic effect. Here, one can see a nonlinear relation of Δ­(Δ*G* ^⧧^) with Ω_R_ that agrees with what has been observed experimentally (e.g., Figure 3C in ref 4). Preliminary experimental investigations^4^ suggest a nonlinear trend between Δ­(Δ*G* ^⧧^) and Ω_R_, and future experimental investigations should focus on measuring more data points to determine the fundamental scaling relations.

Figure 3b presents the temperature dependence of the VSC rate constant using Eyring-type analysis for reactions outside the cavity (black) and inside a resonant cavity under various light-matter coupling strengths (colors). The rate constants were obtained from HEOM simulations (filled circles), and fitted by the least-squares fitting procedure to obtain linearity (thin lines), as well as from FGR rate theory (open circles). If one assumes the rate constant could be described by an Eyring-type equation (transition state theory), then $\ln\left(k/T\right)\propto -\frac{\Delta H^{‡}}{k_{B}}\cdot \frac{1}{T}+\frac{\Delta S^{‡}}{k_{B}}$ , where the slope is related to Δ*H* ^⧧^ and the *y*-intercept to Δ*S* ^⧧^. In Figure 3b it can be seen that as Ω_R_ increases, both Δ*H* ^⧧^ and Δ*S* ^⧧^ changes. Here, both the HEOM simulation and the FGR rate theory predict the same trend. We emphasize that based on our current theory, the VSC reaction mechanism is not related to the direct modification of the Δ*H* ^⧧^ nor Δ*S* ^⧧^ (as also been suggested from the previous theories^20^), but rather how cavity can mediate vibrational excitations (see eq 6), and described in eq 10.

Another factor that influences the VSC-rate constant is the cavity lifetime τ_c_ (which also explicitly shows up in the analytic expression of Γ_Q_(ω) and R̃(ω) . However, we find that *k*/*k*_0_ is not particularly sensitive to τ_c_, and the magnitude of suppression will be maximized under τ_c_ → ∞ limit, but start to converge when τ_c_ ≥ 300 fs (see Supporting Information 8).

### Null VSC Results due to Low Reaction Barrier

Recent experiments^60,61^ on CN radical-hydrogen atom abstraction reactions do not reveal any noticeable change in the rate constant, even though the molecular system is under the VSC condition. These seemingly null results on the VSC effect could indirectly inform the fundamental mechanism and limitations of VSC-induced rate constant modifications, providing insights into when VSC will not be able to change rate constants (as negative controls). Based on our theory, the cavity can modify the rate constant *k*_1_, and when *k*_1_ ≪ *k*_2_, *k*_3_, such that *k* ≈ *k*_1_ (eq 6), the cavity effect on *k*_1_ manifests in the overall rate of reaction *k*. If the reaction mechanism in eq 6 no longer holds, then coupling to the cavity will not modify the rate constant at all. This will indeed be the case when the potential barrier height is even lower than the first vibrational excited state, and ground state tunneling |ν_L_⟩ → |ν_R_⟩ becomes the dominating reactive channel.^60^

To confirm this hypothesis, we contract a model reactive potential depicted in Figure 4a with a lower barrier energy *E* ^⧧^, such that there is only one localized vibrational state, |ν_L_⟩ (orange) in the reactant side. The details of the parameters are provided in Supplemental Note 1 (model 2). The frequency of the spectator mode ω_Q_ in this model is matched to |ν_L_⟩ → |ν_2_⟩ transition. Note that one can still achieve VSC when coupling *q*_c_ with Q when ω_c_ = ω_Q_.

Figure 4b presents the HEOM results of *k*/*k*_0_ under various cavity frequency ω_c_ with a strong coupling Ω_R_ = 60 cm^–1^. We do not observe any noticeable change of *k*/*k*_0_ over a wide range of cavity frequencies, agreeing with the null experimental results in ref 60. These results can be interpreted from the vibrational population dynamics provided in Figure S3 in Supporting Information 4, where the vibrationally excited states do not actively participate in the forward reaction. Similarly, in ref 61, a range of Ω_R_ were used in the experiments (25 ≤ Ω_R_ ≤ 75 cm^–1^), and one still finds null VSC results. Figure 4c presents *k*/*k*_0_ over various Ω_R_ under the resonance condition ω_c_ = ω_Q_, and predicts the same null effect. The current theory based on the mechanism in eq 6 supports the null VSC results recently discovered in the experiments.^60,61^

As discussed above, we have seen two main mechanisms by which chemical reactions can proceed: (a) the initial vibrational excitation as the limiting reaction step (described by eq 6) and (b) the direct tunneling from reactants to products as the main reactive pathway. Whether a reaction proceeds by the first or second mechanism is dictated by the position of the reaction barrier and the conditions are *E*_I_^⧧^ > ω_0_ (for case a) or *E*_II_^‡^ < ω_0_ (for case b), respectively. Thus, for two chemically similar reactions, only the one obeying mechanism (a) will be modified by the VSC effects. Experimental measurements^62^ of the barrier height *E* ^⧧^ based on infrared absorption spectroscopy (in conjunction with DFT simulations) suggest that (I) alcoholysis reaction between phenylisocyanate and cyclohexanol has a *E*_I_^⧧^ = 6.7 kcal/mol (2343 cm^–1^), and for a similar alcoholysis reaction (II) between 2,4-toluene-diisocyanate and chloralhydrate, the activation energy is *E*_II_^⧧^ = 2.8 kcal/mol (973 cm^–1^). Interestingly, *E*_I_^⧧^ > {ω_0_,ω_Q_} > *E*_II_^⧧^, and reaction (I) has been investigated by Simpkins et al.^7^ in a cavity under VSC and found a sharp resonance suppression. The current theory thus predicts that coupling reaction (II) to the cavity will give null results (similar to Figure 4) due to its low barrier. Future experiments are encouraged to test this theoretical prediction.

### Collective Coupling Effect

The current theory *k*_VSC_ (eq 10) also exhibits collectiveness of the rate constant modification, originating from the collective coupling between *R*_0_ and $\left\{Q_{j}\right\}$ through ${Ĥ}_{Q}$ in eq 2, and the light-matter couplings between *q*_c_ and $\left\{Q_{j}\right\}$ through ${Ĥ}_{LM}$ (see eq 4). We consider that *N* solvent DOF $Q_{j}$ have identical frequency ω_Q_, with details in Supporting Information 5. When $C_{j}$ are identical, and there is no disorder in the light-matter coupling angles, only the polaritonic states show up in *J*_eff_(ω) meaning that the *N* – 1 dark states present are completely decoupled from the reaction coordinate and will not affect the rate of reaction. This can also be understood from a normal-mode analysis approach^31^ shown in Supporting Information 7. As such, the dark vibrational modes are no longer directly coupled to *R*_0_ and will not influence the rate constant. When there are certain disorders in $C_{j}$ and also in cos φ_*j*_, the dark vibrational modes will have a finite spectral density contribution (see Figures S8 and S9 in Supporting Information 5), and will gradually diminish the VSC collective effect.

Figure 5a presents *k*/*k*_0_ with *N* = 10^4^, at various light-matter coupling strength per molecule *g*_c_ = η_c_ω_c_μ_Q_, where the collective Rabi splitting is $\Omega_{R}=2\sqrt{N}g_{c}$ (cf. eq 5). The overall collective coupling between $\left\{Q_{j}\right\}$ and *R*_0_ is kept fixed at Λ = 1.71 cm^–1^ (see eq 3). Note that as $\sqrt{N}$ always pairs up with η_c_ in *k*_VSC_, the results are thus identical to the case of *N* = 1 with a much larger η_c_. The theory still predicts a sharp resonance suppression when ω_c_ = ω_Q_.

**Figure 5:**
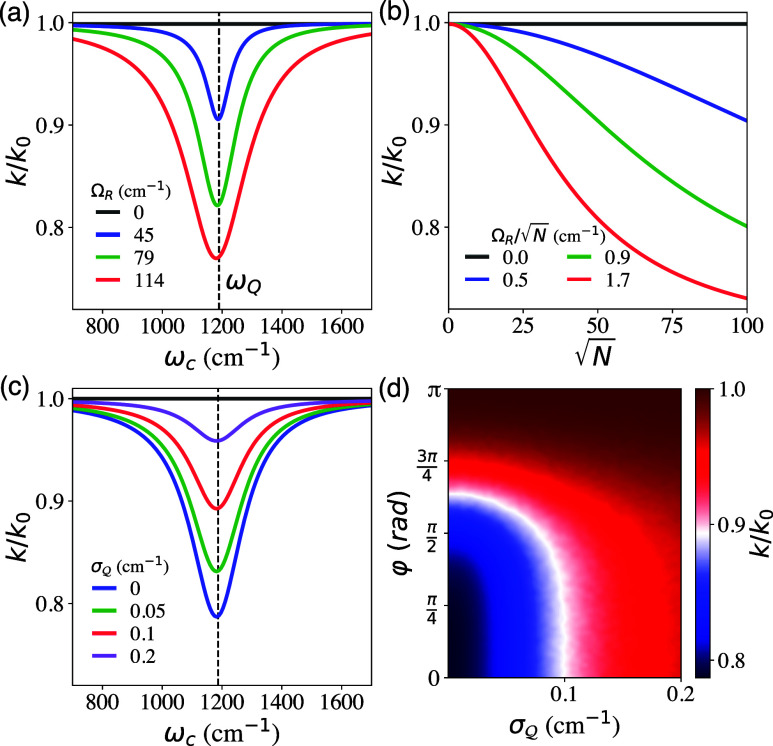
Collective Effect predicted by the FGR theory. (a,b). *k*/*k*_0_ as a function of (a) the cavity frequency under different Ω_R_ and (b) *N* while the light-matter coupling per molecule *g*_c_ is kept constant, with identical $C_{j}$ for each vibrational mode. The dipole is assumed to be fully aligned, such that χ = 1. (c) *k*/*k*_0_ as a function of ω_c_, for a system with *N* = 1000, Ω_R_ = 100 cm^–1^, and χ = 1, with $C_{j}$ disorder sampled from a normal distribution with $\sigma_{Q}$ . (d) *k*/*k*_0_ for *N* = 1000, as a function of $C_{j}$ disorder $\left(\sigma_{Q}\right)$ and the angle {φ_*j*_} between the dipole and cavity polarization, which are sampled from a uniform distribution in the range {φ_*j*_} ∈ [0, π].

Figure 5b further present *k*/*k*_0_ as a function of increasing *N*, while keeping the light-matter coupling strength per molecule (*g*_c_) a constant, agreeing to the fundamental scaling relation *k*_VSC_ ∝ 1/*N* in eq 11. The theory demonstrates the same essential feature of the collective coupling effects observed in experiments,^4^ namely the rate constant suppression as the number of molecules (or their concentration) increases. We want to point out that in our early work^44,45^ we have demonstrated the rate constant enhancement based on strong coupling between a single *R*_0_ and *q*_c_. When the cavity mode is explicitly coupled to both *Q* and *R*_0_, one would expect competition between these two types of mechanisms.^21^ However, under the collective coupling limit *N* ≫ 1, the enhancement effects will be negligibly small as η_c_ → 0, and the leading effect will be the resonance suppression reported here.

Note that the light-matter coupling strength per molecule, $\eta_{c}\propto \Omega_{R}/\sqrt{N}$ , used in Figure 5 may still be much larger than what is typically expected in VSC experiments. Early theoretical estimates^16,49^ suggest that at least *N* = 10^6^ to 10^12^ molecules must be collectively coupled to achieve the experimentally^48^ observed Rabi splitting Ω_R_. In contrast, recent experimental work by Xiong^63^ shows as few as *N* = 10^4^ molecules per cavity mode under the vibrational strong coupling condition. Future experimental and theoretical work is necessary to address this fundamental question.

Figure 5c,d further explores the effects of having disorders in $C_{j}$ , using the exact expression for *J*_eff_(ω). Figure 5c shows the cavity frequency dependence of *k*/*k*_0_ for *N* = 10^3^ at a collective Rabi splitting of Ω_R_ = 100 cm^–1^. The values of $C_{j}$ are taken from a normal distribution with standard deviation $\sigma_{Q}$ , while Λ is kept constant, and there is no angle disorder for the light-matter couplings, such that χ = 1 (cf. eq 9). As shown in Figure 5c, increasing the disorder in the coupling (increasing $\sigma_{Q}$ ) decreases the cavity effects. This is because adding disorder to the couplings allows the dark states coupling to *R*_0_ to show up in *J*_eff_(ω) (see Supporting Information 5, Figure S9), thus reducing the cavity effect as these dark modes have the same frequency as the vibrations outside the cavity. Nevertheless, the cavity modification effect will survive with a moderate magnitude of disorder, and the fundamental scaling indicated in eq 11 is also preserved (see Figure S9b in Supporting Information 5). We have also explored the inhomogeneous broadening of the solvent frequency ω_Q_ using Gaussian static disorders, with details provided in Supporting Information 5, Figure S10. We found that increasing the magnitude of the static disorder slightly decreases the value of *k*/*k*_0_, but it does not change the sharp resonance behavior of its cavity frequency dependence.

Figure 5d shows *k*/*k*_0_ for *N* = 1000 at resonance and Ω_R_ = 100 cm^–1^, with $C_{j}$ disorder sampled from a normal distribution with standard deviation $\sigma_{Q}$ and the random distribution of the angle between the dipole and cavity field {φ_*j*_} ∈ [0, φ], with φ changing from 0 (fully ordered) to π (isotropic). One can see that with a small $C_{j}$ disorder, the collective VSC effect will survive even for a random disorder of dipole angle for {φ_*j*_} ∈ [0, 3π/4], as dark modes have a small contribution in the spectral density (see Figure S9 of Supplementary Note 5). Here, we demonstrate that the extent of the dark states highly depends on the disorder of the system, where the cavity can modify the reaction rates even in the presence of such states.

### Resonance at the Normal Incidence

The dispersion relation of a FP microcavity^12,17,64^ is

12 $$\omega_{k}\left(k_{∥}\right)=\frac{c}{n_{c}}\sqrt{k_{⊥}^{2}+k_{∥}^{2}}$$

where *c* is the speed of light in vacuum, and we have assumed the refractive index inside the cavity *n*_c_ ≈ 1. The expression of the many-mode Hamiltonian is provided in Supporting Information 9, Section A. When *k*_∥_ = 0 (or θ = 0), the photon frequency is ω_c_ ≡ ω_**k**_(*k*_∥_ = 0) = *ck*_⊥_, which is the cavity frequency we introduced in eq 4. Experimentally, it is observed that only at *k*_∥_ = 0, ω_**k**_ = ω_Q_ (known as the normal incidence condition) will there be VSC effects.^2,7,65^ Here, we generalize the single-mode expression in eq 10 into many modes to understand the normal incidence condition. By considering many modes, the FGR rate constant inside a 1D FP cavity (1-dimension along the *k*_∥_ direction) reduces back to the single mode case because of the van-Hove-type singularity^66^ in the photonic DOS.^46^ As a result, inside a 1D FP cavity, VSC modification occurs only at the normal incidence ω_c_ = ω_0_.

For molecules coupled inside a 2D FP cavity (2-dimensional along the in-plane direction), the FGR rate constant is expressed as follows

13 kVSC2D=2|Δx|2∫0∞dωΛωQ2·ωΓQ2D(ω)·A0(ω−ω0)·e−βℏω0[ωQ2−ω2+R̃2D(ω)]2+[ωΓQ2D(ω)]2,

where Γ_Q_^2D^(ω) and ${\mathrm{R̃}}^{2D}\left(\omega \right)$ are generalized expressions for a 2D FP cavity, both of which contain the effective photon lifetime due to propagation in the in-plane direction^46^ $\tau_{∥}\left(\omega \right)=D\sqrt{k_{⊥}^{2}+k_{∥}^{2}}/\left(c\cdot k_{∥}\right)$ with D as the effective mode diameter.^46^ The detailed expressions are provided in Supporting Information 9. The effective photon lifetime has a maximum value at *k*_∥_ = 0, leading to the largest magnitude of rate constant suppression. The finite in-plane momentum of the mode effectively decreases the effective lifetime of the thermal photon in a given mode volume.^46^ Note that the interpretation of τ_∥_(ω) is consistent with the polariton transport picture,^52,67–69^ because for *k*_∥_ = 0, the group velocity for photon and polariton is zero, leading to no transport. And for *k*_∥_ > 0, the group velocity for thermal photons is fast, leading it to travel a large distance and not be confined in a given region.^46^ Overall, this leads to a sharp resonance at ω_c_ = ω_Q_ when *k*_∥_ = 0. The rate constant expression *k*_VSC_^2D^ (eq 13) is the third key result of this paper, which predicts that the maximum suppression of *k*/*k*_0_ happens at the normal incidence at *k*_∥_ = 0.

Figure 6 presents the VSC-suppressed rate constant using the FGR expression (eq 10) under different Rabi splitting Ω_R_ values and a cavity lifetime τ_c_ = 500 fs. Here, we consider the molecule coupled inside (a) a 1D FP cavity and (b) a 2D FP cavity. Figure 6a presents the results of *k*/*k*_0_ value using eq 10 (dashed line) or numerically evaluating the frequency integral related to the photonic DOS (solid line). The result is visually (near) identical to the single-mode case due to the van-Hove-type singularity in the 1D DOS, depicted in the dashed curve. Figure 6b presents *k*/*k*_0_ for many $Q_{j}$ DOF coupled to many modes inside a 2D FP cavity, using eq 7, and the *k*_VSC_^2D^ expression in eq 13, with the effective mode diameter D=3.33μm , which is a typical value estimated^46^ from the VSC experiments.^48^ One can see that the resonance peak is still sharply centered around ω_c_ = ω_Q_, where ω_c_ is the normal incidence frequency in a 2D FP cavity. The theory, *k*_VSC_^2D^ in eq 13 thus exhibits the features of (1) resonance condition, (2) normal incidence condition and operates under the (3) collective coupling regime and (4) thermally activated condition.

**Figure 6:**
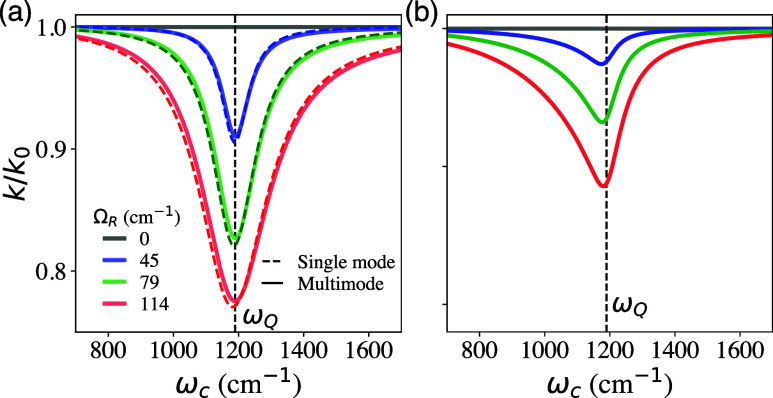
Normal Incidence effect for an 1D and 2D FP cavity. FGR rate profiles of *k*/*k*_0_ as a function of ω_c_, where the cavity lifetime is τ_c_ = 500 fs. Results are presented for various light-matter coupling strengths. (a) FGR rate profiles inside a 1D FP cavity. (b) FGR rate profiles for many mode cases inside a 2D FP cavity.

## Conclusions

To summarize, we present a microscopic mechanism and an analytic rate theory that provide theoretical insights into the resonance VSC suppression of the rate constant under normal-incidence conditions, collective light-matter coupling, and the thermally activated regime. For the model reaction, the reactant vibrational excitation |ν_L_⟩ → |ν_L_^′^⟩ is the rate-limiting step and thus controls the overall apparent rate constant. Coupling to the cavity explicitly splits the effective solvent spectral density that influences the dynamics of the reaction coordinate (see Figure 2a), and thus reduces the thermal activation rate *k*_1_, hence reducing the overall apparent rate constant. For a single molecule coupled to a single mode cavity, the theory *k*_VSC_ (eq 10) exhibits (i) a sharp resonance suppression behavior^1,7^ at ω_c_ = ω_Q_ (Figure 2); (ii) nonlinear scaling^1,4^ of *k*/*k*_0_ with respect to Ω_R_ (Figure 3a), as well as the nonlinear scaling^1,4^ of ΔΔ*G* ^‡^ with respect to Ω_R_; (iii) modification of both effective activation Entropy and Enthalpy^4^ (Figure 3b). (iv) The null effect^60,61^ because of a very low reaction barrier (Figure 4), such that the mechanistic in eq 6 no longer hold. The analytic theory (eq 7) agrees very well with the numerically exact simulations for all cases. The theory *k*_VSC_ (eq 10) also exhibits the collective effect, where both the collective solvent–solute coupling Λ as well as the collective light-matter coupling Ω_R_ show up in the rate constant expression. The theory *k*_VSC_^2D^ in eq 13 thus demonstrates all key features in the VSC experiments.

The theory (eq 10 or eq 13) has several interesting predictions that can be experimentally tested. (a) The magnitude of the VSC effect on rate constant scales (eq 11) as Ω_R_^2^ or *N*, which should be experimentally checked. (b) For two chemically similar reactions, if one operates under the mechanism in eq 6 and satisfies *k*_1_ ≪ *k*_2_, *k*_3_ but the other does not (due to the low reaction barrier), then the current theory predicts that there will be a VSC effect for the first reaction but not for the second one. (c) Based on *k*_VSC_^2D^ (eq 13), the action spectrum should have a longer tail at the red frequency (ω_c_ < ω_Q_). Based on the current theory, we recommend future experimental investigations to focus on (1) confirming the scaling relation of *k*/*k*_0_ with respect to Ω_R_ (or *N*) with more data points. (2) confirming potentially null VSC results in the alcoholysis reaction between 2,4-toluene-diisocyanate and chloralhydrate due to its low barrier,^62^ which will greatly inform the VSC mechanism, given that the similar alcoholysis reaction between phenylisocyanate and cyclohexanol has shown VSC resonance suppression effects.^7^ (3) More data points on the cavity frequency dependence of *k*/*k*_0_ to provide the shape of the action spectrum. (4) control the dipole orientations^70,71^ (so one can control χ in eq 9) and measure the corresponding VSC effects.

The cavity-mediated vibrational energy transfer process has been experimentally verified when the system is directly pumped into polariton states (as opposed to the VSC ground state reactions, which occur under dark and thermally activated conditions). These experiments by Xiong and co-workers,^72^ together with the cavity Molecular Dynamics simulations^29,73,74^ and the FGR type of theory,^30,39^ indicate that the cavity can indeed mediate the vibrational energy transfer rates under the nonequilibrium polariton pumping conditions. These experiments^72^ and theoretical works may be closely connected with the currently investigated VSC reactions. Using simple models to understand those nonequilibrium pumping VSC experiments^72^ would also be valuable, as this could indirectly probe the fundamental mechanism of the VSC-modified rate constant. We also need to point out the limitations of the model systems in eq 2. Although it could be an appropriate model for describing the vibrational energy transfer between solute and solvent,^29^ any potentially relevant anharmonic interaction effects are not captured by the bilinear interaction term in eq 2 of the present manuscript.

The current rate theory (eqs 10 or eq 13) requires several key parameters as input, including the collective light-matter coupling Ω_R_, Q mode reorganization energy Λ, cavity frequency ω_c_, etc. These key parameters can be extracted from ab initio simulations of a particular reaction^28^ or atomistic cavity molecular dynamics simulations,^29^ as well as from experiments that often report line shape. In an analogy, the situation is similar to Marcus theory in electron transfer reactions, where key parameters are required before one can use it as a predictive rate constant expression.

## Supplementary Material



## Notes

Details of model systems; exact quantum dynamics using hierarchical equations of motion; rate constant calculations; derivation of *J*_eff_(ω); *J*_eff_(ω) simplification and scaling analysis; normal mode analysis; cavity lifetime dependence; many modes Hamiltonian and the normal incidence condition for VSC effects (PDF)
