# Supplementary material for: Theoretical Insights into the Resonant Suppression Effect in Vibrational Polariton Chemistry
Source: J Am Chem Soc. 2025 Jun 2;147(23):19727–37. doi: 10.1021/jacs.5c03182 (PMC12164353; doi:10.1021/jacs.5c03182)
Supplement: Supplementary file 1 [file ja5c03182_si_001.pdf]

Supporting Information: Theoretical insights into the Resonant Suppression Effect in Vibrational Polariton Chemistry

Sebastian Montillo Vega[#,†] Wenxiang Ying[#,†] and Pengfei Huo[*,†,‡,¶]

†*Department of Chemistry, University of Rochester, 120 Trustee Road, Rochester, New York 14627, USA*

‡*The Institute of Optics, Hajim School of Engineering, University of Rochester, Rochester, New York 14627, USA*

¶*Center for Coherence and Quantum Science, University of Rochester, Rochester, New York 14627, USA*

[#]S.M.V. and W.Y. contributed equally to this work.

E-mail: pengfei.huo@rochester.edu

Contents

Supporting Information 1: Details of Model Systems **S4**

A. Details of the Model Hamiltonian S4

B. Detailed expression of the broadening function $\mathcal{A}_0$ S6

C. Model Hamiltonian used in the HEOM simulations and model Parameters S6

Supporting Information 2: Exact Quantum Dynamics using Hierarchical Equations of Motion **S12**

A. A brief introduction to *dissipaton* S12

B. Bath TCF decomposition schemes S14

C. Details of the HEOM Calculations S15

D. Infrared Spectra of the Bare-molecular System S16

Supporting Information 3: Rate Constant Calculations S17

Supporting Information 4: Population Dynamics and Kinetics Analysis S19

A. Population Dynamics Analysis for Resonance Suppression S19

B. Population Dynamics for Null VSC effect S21

C. Population Dynamics and Rate Constants for an Asymmetrical Double-Well Model Coupled to the Cavity S21

Supporting Information 5: Derivation of $J_{\text{eff}}(\omega)$ S27

A. The most general case S27

B. Uniform light-matter and spectator - reaction coordinate interactions S31

C. Uniform $\mathcal{Q}_j - R_0$ couplings and disorders in dipole orientations S32

D. Uniform light-matter interaction and $\mathcal{C}_j$ disorder S35

E. Static Disorder in spectator modes' frequency ω_{Q} S37

F. Generalized case in C and D S38

Supporting Information 6: $J_{\text{eff}}(\omega)$ Simplification and Scaling Analysis S41

A. Lossless cavity S41

B. Scaling analysis S42

Supporting Information 7: Normal Mode Analysis S44

A. Uniform light-matter coupling and spectator mode - Rxn interactions S46

B. $\mathcal{Q}_j - R_0$ coupling disorder S48

Supporting Information 8: Cavity Lifetime Dependence S49

Supporting Information 9: Many Modes Hamiltonian and the Normal Incidence Condition for VSC effects S50

A. The many-mode Hamiltonian S50

B. Effective spectral density with many cavity modes: the most general case S52

C. Effective spectral density with many cavity modes: analytic form with approximations S52

D. Numerical details of evaluating $k_{\mathrm{VSC}}^{\mathrm{2D}}$ S56

References **S57**

Supporting Information 1: Details of Model Systems

A. Details of the Model Hamiltonian

The Hamiltonian of the system studied in this work is given by

$$\hat{H} = \hat{H}_{\mathrm{M}} + \hat{H}_{\mathcal{Q}} + \hat{H}_{\mathrm{LM}} + \hat{H}_{\nu} + \hat{H}_{\mathrm{loss}}, \tag{S1}$$

with a detailed expression of spectator mode term $\hat{H}_{\mathcal{Q}}$ and light-matter interaction term $\hat{H}_{\mathrm{LM}}$ are provided in Eq. 2 and Eq. 4 of the main text,respectively.

Here, $\hat{H}_{\mathrm{M}} = \hat{T}_0 + \hat{V}(\hat{R}_0)$ is the molecular Hamiltonian, with $\hat{T}_0 = \hat{P}_0/2M$, and M is the effective mass associated with reaction coordinate $\hat{R}_0$. For models 1 and 2, we consider a double-well (DW) potential to model the ground state chemical reaction[1,2]

$$V(\hat{R}_0) = -\frac{M\omega_{\mathrm{b}}^2}{2}\hat{R}_0^2 + \frac{M^2\omega_{\mathrm{b}}^4}{16E^{\ddagger}}\hat{R}_0^4.$$

For model 1, $M = 1836$ a.u., $\omega_{\mathrm{b}} = 1000$ cm^{-1} is the barrier frequency, and $E^{\ddagger} = 2250$ cm^{-1} is the barrier height. For model 2, $M = 1836$ a.u., $\omega_{\mathrm{b}} = 1500$ cm^{-1} is the barrier frequency, and $E^{\ddagger} = 2300$ cm^{-1} is the barrier height. The details of parameters are provided in Table S1 and Table S2.

For model 3, we consider an asymmetrical double-well potential, with the following

$$V(\hat{R}_0) = -\frac{M\omega_{\mathrm{b}}^2}{2}\hat{R}_0^2 - c\hat{R}_0^3 + \frac{M^2\omega_{\mathrm{b}}^4}{16E^{\ddagger}}\hat{R}_0^4.$$

where the $-c\hat{R}_0^3$ term provides the asymmetry fo the potential. The details of the parameters are provided in Table S3.

For the matter Hamiltonian $\hat{H}_{\mathrm{M}} = \hat{T} + \hat{V}$, the vibrational eigenstates $|\nu_i\rangle$ and eigenenergies E_i are obtained by solving $\hat{H}_{\mathrm{M}}|\nu_i\rangle = E_i|\nu_i\rangle$ numerically using the discrete variable representation (sinc-DVR) basis[3] with 1001 grid points in the range of $R_0 \in [-2.5, 2.5]$. To facilitate the mechanistic studies, for Model I, we diabatize the two lowest eigenstates and obtain two energetically degenerate

diabatic states

$$|\nu_{\rm L}\rangle = \frac{1}{\sqrt{2}}\big(|\nu_0\rangle + |\nu_1\rangle\big), \quad |\nu_{\rm R}\rangle = \frac{1}{\sqrt{2}}\big(|\nu_0\rangle - |\nu_1\rangle\big),$$

both with energies of $\mathcal{E} = (E_1 + E_0)/2$ and a small tunneling splitting of $\Delta = (E_1 - E_0)/2$. Similarly, for the vibrational excited states $\{|\nu_2\rangle, |\nu_3\rangle\}$, we diabatize them and obtain the first excited *diabatic* vibrational states in the left and right wells as follows

$$|\nu'_{\rm L}\rangle = \frac{1}{\sqrt{2}}\big(|\nu_2\rangle + |\nu_3\rangle\big), \quad |\nu'_{\rm R}\rangle = \frac{1}{\sqrt{2}}\big(|\nu_2\rangle - |\nu_3\rangle\big),$$

with degenerate diabatic energy of $\mathcal{E}' = (E_3 + E_2)/2$ and a tunneling splitting of $\Delta' = (E_3 - E_2)/2 \approx 47.68$ cm^{-1}. A schematic representation of these diabatic states is provided in Fig. 1d of the main text. Based on the two diabatic states $|\nu_{\rm L}\rangle$ and $|\nu'_{\rm L}\rangle$ in the left well, we define the quantum vibration frequency of the reactant as $\omega_0 \equiv \mathcal{E}' - \mathcal{E} = 1189.7$ cm^{-1}, which is directly related to the quantum transition of $|\nu_{\rm L}\rangle \to |\nu'_{\rm L}\rangle$.

Further, $\hat{H}_\nu$ in Eq. S1 is the system-bath Hamiltonian where each matter DOF is linearly coupled to a set of dissipative phonon bath modes

$$\begin{aligned} \hat{H}_\nu = \frac{1}{2}\sum_i \Big[\hat{p}_i^2 + \omega_i^2\Big(\hat{x}_i - \frac{c_i}{\omega_i^2}\hat{R}_0\Big)^2\Big] \\ + \frac{1}{2}\sum_{j,\zeta}\Big[\hat{p}_{j,\zeta}^2 + \omega_{j,\zeta}^2\Big(\hat{x}_{j,\zeta} - \frac{c_{j,\zeta}}{\omega_{j,\zeta}^2}\mathcal{Q}_j\Big)^2\Big], \end{aligned} \tag{S2}$$

where the frequencies (ω_i, ω_ζ) and couplings (c_i, c_ζ) are defined from the spectral density function as follows

$$J_0(\omega) \equiv \frac{\pi}{2}\sum_i \frac{c_i^2}{\omega_i}\delta(\omega - \omega_i) = \frac{2\lambda_0\gamma_0\omega}{\omega^2 + \gamma_0^2}, \tag{S3a}$$

$$J_{\rm Q}(\omega) \equiv \frac{\pi}{2}\sum_\zeta \frac{c_\zeta^2}{\omega_\zeta}\delta(\omega - \omega_\zeta) = \frac{2\lambda_{\rm Q}\gamma_{\rm Q}\omega}{\omega^2 + \gamma_{\rm Q}^2}. \tag{S3b}$$

We use Drude-Lorentz model spectral density, with $\gamma_0 = 200$ cm^{-1}, $\gamma_{\rm Q} = 6000$ cm^{-1} for the bath characteristic frequencies and $\lambda_0 = 83.6$ cm^{-1}, $\lambda_{\rm Q} = 0.147$ cm^{-1} for the reorganization energies,

similar to the previous work.[4]

Finally, the cavity mode interacts with the outside cavity electromagnetic field that acts as a bath for the cavity mode, thus describing the cavity loss

$$\hat{H}_{\rm loss} = \frac{1}{2}\sum_{\upsilon}\left[\hat{p}_{\upsilon} + \omega_{\upsilon}^{2}\left(\hat{x}_{\upsilon} - \frac{c_{\upsilon}}{\omega_{\upsilon}^{2}}\hat{q}_{\rm c}\right)^{2}\right], \tag{S4}$$

where the couplings (c_υ) and frequencies (ω_υ) are sample from the photon-loss bath spectral density $J_{\rm loss}(\omega) \equiv (\pi/2)\sum_{\upsilon}(c_{\upsilon}^{2}/\omega_{\upsilon})\delta(\omega-\omega_{\upsilon}) = (\omega/\tau_{\rm c})\exp(-\omega/\omega_{\rm m})$, where $\tau_{\rm c}$ is the cavity lifetime,[5] and we had assumed that photon loss satisfies strict Ohmic dissipation. In other words, as the cutoff frequency $\omega_{\rm m}\to\infty$, the photon bath dynamics reach the Markovian limit.[5,6] A detailed discussion can be found in Ref. 5.

B. Detailed expression of the broadening function $\mathcal{A}_0$

For the fixed energy level between $|\nu_{\rm L}\rangle$ and $|\nu'_{\rm L}\rangle$ states, the FGR rate constant is expressed as $k_{\rm VSC} = \kappa(\omega_0)$, where $\hbar\omega_0$ is the energy difference for the transition. However, R_0 also couples to its own solvent bath (see Eq. S2), and has fluctuating energy levels. To account for this effect, we add a broadening function $\mathcal{A}_0(\omega-\omega_0)$ to the frequency ω_0, with a Lorentzian shape[5,7] (for the case under the homogeneous limit[8]) expressed as

$$\mathcal{A}_0(\omega-\omega_0) = \frac{1}{\pi}\frac{\Gamma_0}{(\omega-\omega_0)^2+\Gamma_0^2}, \tag{S5}$$

with the broadening parameter[5] expressed as

$$\Gamma_0^2 = (\epsilon_z^2/\pi)\int_0^{\infty} d\omega\; J_0(\omega)\coth(\beta\omega/2), \tag{S6}$$

and $\epsilon_z \equiv \langle\nu'_{\rm L}|\hat{R}_0|\nu'_{\rm L}\rangle - \langle\nu_{\rm L}|\hat{R}_0|\nu_{\rm L}\rangle$.

C. Model Hamiltonian used in the HEOM simulations and model Parameters

For the HEOM simulations of the rate constant and the population dynamics, we consider the cavity mode (as well as its loss bath) as part of the bath degrees of freedom (DOF). This is done

by performing a harmonic analysis of the equations of motion of the cavity-loss sub-system leading to the effective Hamiltonian (see Ref. 5 for details)

$$\hat{H}_{\text{eff}}^{(1)} = \hat{H}_{\text{LM}} + \hat{H}_{\text{loss}} = \sum_n \frac{\hat{\pi}_n^2}{2} + \frac{\omega_n^2}{2}\left(\hat{x}_n - \frac{\tilde{c}_n}{\omega_n^2}\hat{\mathcal{Q}}\right)^2, \tag{S7}$$

where the normal modes $\{\hat{x}_n\}$ are directly coupled to the spectator mode $\mathcal{Q}$, with couplings and frequencies (in the Markovian limit, which considers $\gamma_{\text{c}} \to \infty$) are given by the spectral density

$$J_{\text{eff}}^{(1)}(\omega) = \frac{2\eta_{\text{c}}^2\omega_{\text{c}}^3\tau_{\text{c}}^{-1}\omega}{(\omega_{\text{c}}^2 - \omega^2)^2 + (\tau_{\text{c}}^{-1}\omega)^2}, \tag{S8}$$

where τ_{c} is the cavity lifetime, ω_{c} the cavity frequency and η_{c} the light-matter coupling strength. In this representation, the cavity and its loss are part of the spectator mode bath modes, therefore it is not necessary to simulate the cavity DOF explicitly (e.g. by means of Fock states), thus significantly reducing the computational costs of HEOM.[5] Then, the system Hamiltonian can be rewritten as

$$\hat{H} = \hat{H}_{\text{M}} + \hat{H}_{\mathcal{Q}} + \hat{H}_{\text{B}} + \hat{H}_{\text{SB}} + \hat{H}_{\text{ren}}, \tag{S9}$$

where each term is expressed as follows

$$\hat{H}_{\text{B}} = \tfrac{1}{2}\sum_i \left(\hat{p}_i^2 + \hat{x}_i^2\omega_i^2\right) + \tfrac{1}{2}\sum_j \left(\hat{p}_j^2 + \hat{x}_j^2\omega_j^2\right) + \tfrac{1}{2}\sum_n \left(\hat{\pi}_n^2 + \hat{x}_n^2\omega_n^2\right), \tag{S10a}$$

$$\hat{H}_{\text{SB}} = \hat{F}_0 \otimes \hat{R}_0 + \left(\hat{F}_{\text{Q}} + \hat{F}_{\text{eff}}^{(1)}\right) \otimes \hat{\mathcal{Q}}, \tag{S10b}$$

$$\hat{H}_{\text{ren}} = \lambda_0\hat{R}_0^2 + \left(\lambda_{\text{Q}} + \Lambda_{\text{c}}\right)\hat{\mathcal{Q}}^2. \tag{S10c}$$

In particular, the terms $\hat{H}_{\text{B}}$, $\hat{H}_{\text{SB}}$ and $\hat{H}_{\text{ren}}$ are the bath Hamiltonian, the molecular - bath coupling, expressed in terms of the collective bath coordinates

$$\hat{F}_0 = \sum_i c_i\hat{x}_i, \quad \hat{F}_{\text{Q}} = \sum_j c_j\hat{x}_j, \quad \hat{F}_{\text{eff}}^{(1)} = \sum_n \tilde{c}_n\hat{x}_n, \tag{S11}$$

and the bath reorganization energy is expressed as

$$\lambda_0 = \sum_i \frac{c_i^2}{2\omega_i^2}, \quad \lambda_{\mathrm{Q}} = \sum_j \frac{c_j^2}{2\omega_j^2}, \quad \Lambda_{\mathrm{c}} = \eta_{\mathrm{c}}^2 \omega_{\mathrm{c}}, \tag{S12}$$

whose couplings and frequencies are sampled from the Drude - Lorentz spectral densities described as

$$J_0(\omega) = \frac{2\lambda_0 \gamma_0 \omega}{\omega^2 + \gamma_0^2}, \quad J_{\mathrm{Q}}(\omega) = \frac{2\lambda_{\mathrm{Q}} \gamma_{\mathrm{Q}} \omega}{\omega^2 + \gamma_{\mathrm{Q}}^2}. \tag{S13}$$

We briefly summarize the main model parameters used in numerical simulations in Table S1 and Table S2 below. Model 1 is used for all results except for Fig. 4 of the main text and Fig. S3 in this SI. Model 2 is used to demonstrate the null VSC effect in Fig. 4 of the main text. Model 3 is used to demonstrate the robustness of the proposed mechanism in an asymmetrical double well potential $V(R_0)$ for the model reaction, with the results provided in Supplementary Note 4.

Table S1: Table of major parameters for the VSC reactive model (Model 1)

Parameters of Reaction coordinate (Rxn)	Notation	Value or expression
Effective mass of the reaction coordinate	M	1836 a.u.
Barrier height	E_b	2250 cm^{-1}
Barrier frequency	ω_b	1000 cm^{-1}
Transition frequency ($\|\nu_\mathrm{L}\rangle \to \|\nu'_\mathrm{L}\rangle$)	ω_0	1189.7 cm^{-1}
Tunneling splitting between $\|\nu_\mathrm{L}\rangle$ and $\|\nu_\mathrm{R}\rangle$	Δ	1.02 cm^{-1}
Tunneling splitting between $\|\nu'_\mathrm{L}\rangle$ and $\|\nu'_\mathrm{R}\rangle$	Δ'	47.68 cm^{-1}
$\langle\nu'_\mathrm{L}\| \hat{R}_0 \|\nu'_\mathrm{L}\rangle - \langle\nu_\mathrm{L}\| \hat{R}_0 \|\nu_\mathrm{L}\rangle$	ϵ_z	0.220 a.u.
$\langle\nu'_\mathrm{L}\| \hat{R}_0 \|\nu_\mathrm{L}\rangle$	Δ_x	0.214 a.u.
Broadening parameter	Γ_0	29.23 cm^{-1}
spectator mode reorganization energy	Λ	1.71 cm^{-1}
Number of states used	$\mathcal{F}_0$	5
Parameters of the Spectator Mode ($\mathcal{Q}$)		
Effective mass	M_Q	1 a.u.
Frequency	ω_Q	1189.7 cm^{-1}
Number of states used	$\mathcal{F}_\mathrm{Q}$	3
Coupling to Rxn	$\mathcal{C}_\mathrm{Q}$	0.110 cm^{-1}
Parameters of bath DOFs		
Temperature	T	300 K
Inverse temperature	β	$1/(k_\mathrm{B}T)$
Rxn phonon bath spectral density	$J_0(\omega)$	$2\lambda_0\gamma_0\omega/(\omega^2+\gamma_0^2)$
Characteristic frequency of Rxn phonon bath	γ_0	200 cm^{-1}
Friction parameter of Rxn phonon bath	η_0	0.1
Reorganization energy of the Rxn phonon bath	λ_0	$\eta_0 M\gamma_0\omega_\mathrm{b}/2$ ($\approx$ 83.6 cm^{-1})
Spectator mode bath spectral density	$J_\mathrm{Q}(\omega)$	$2\lambda_\mathrm{Q}\gamma_\mathrm{Q}\omega/(\omega^2+\gamma_\mathrm{Q}^2)$
Characteristic frequency of spectator mode phonon bath	γ_Q	6000 cm^{-1}
Reorganization energy of spectator mode phonon bath	λ_Q	0.147 cm^{-1}
Effective bath spectral density	$J_\mathrm{eff}(\omega)$	Eq. S8
Reorganization of the effective bath	λ_eff	$\eta_\mathrm{c}^2\omega_\mathrm{c}$
Cavity lifetime	τ_c	500 fs

Table S2: Table of parameters for the null reactive model (Model 2)

Parameters of Reaction coordinate (Rxn)	Notation	Value or expression
Effective mass of the reaction coordinate	M	1836 a.u.
Barrier height	E_{b}	2300 cm^{-1}
Barrier frequency	ω_{b}	1500 cm^{-1}
Transition frequency ($\lvert\nu_{\mathrm{L}}\rangle \to \lvert\nu_2\rangle$)	ω_0	1385.2 cm^{-1}
Tunneling splitting between $\lvert\nu_{\mathrm{L}}\rangle$ and $\lvert\nu_{\mathrm{R}}\rangle$	Δ	17.33 cm^{-1}
Number of states used	$\mathcal{F}_0$	6
Parameters of the Spectator Mode ($\mathcal{Q}$)		
Frequency	ω_{Q}	1385.2 cm^{-1}
Coupling to Rxn	$\mathcal{C}_{\mathrm{Q}}$	0.219 cm^{-1}
Parameters of bath DOFs		
Friction parameter of Rxn phonon bath	η_0	0.25
Reorganization energy of the Rxn phonon bath	λ_0	$\eta_0 M\gamma_0\omega_{\mathrm{b}}/2$ (≈ 150 cm^{-1})
Spectator modes bath spectral density	$J_{\mathrm{Q}}(\omega)$	$2\lambda_{\mathrm{Q}}\gamma_{\mathrm{Q}}\omega/(\omega^2+\gamma_{\mathrm{Q}}^2)$
Characteristic frequency of spectator mode phonon bath	γ_0	6000 cm^{-1}
Reorganization energy of spectator mode phonon bath	λ_0	0.11 cm^{-1}
Effective bath spectral density	$J_{\mathrm{eff}}(\omega)$	Eq. S8
Reorganization of the effective bath	λ_{eff}	$\eta_{\mathrm{c}}^2\omega_{\mathrm{c}}$
Cavity lifetime	τ_{c}	500 fs

Table S3: Table of parameters for the asymmetrical double well (Model 3)

Parameters of Reaction coordinate (R_0)	Notation	Value or expression
Effective mass of the reaction coordinate	M	1836 a.u.
Barrier height	E_{b}	2730 cm^{-1}
Barrier frequency	ω_{b}	1030 cm^{-1}
Asymmetric term	c	$2.278 \cdot 10^{-8}$ a.u.
Transition frequency ($\|\nu_1\rangle \rightarrow \|\nu'_{\mathrm{L}}\rangle$)	ω_0	1192.2 cm^{-1}
Tunneling splitting between $\|\nu'_{\mathrm{L}}\rangle$ and $\|\nu'_{\mathrm{R}}\rangle$	Δ'	67.41 cm^{-1}
$\langle\nu'_{\mathrm{L}}\| \hat{R}_0 \|\nu'_{\mathrm{L}}\rangle - \langle\nu_1\| \hat{R}_0 \|\nu_1\rangle$	ϵ_z	12.5 a.u.
$\langle\nu'_{\mathrm{L}}\| \hat{R}_0 \|\nu_1\rangle$	Δ_x	9.20 a.u.
Broadening parameter	Γ_0	39.5 cm^{-1}
$\mathcal{Q}$-mode reorganization energy	Λ	1.76 cm^{-1}
Number of states used	$\mathcal{F}_0$	6
**Parameters of the Spectator Mode ($\mathcal{Q}$)**		
Effective mass	M_{Q}	1 a.u.
Frequency	ω_{Q}	1175 cm^{-1}
Number of states used	$\mathcal{F}_{\mathrm{Q}}$	3
Coupling to Rxn	$\mathcal{C}_{\mathrm{Q}}$	0.110 cm^{-1}
**Parameters of bath DOFs**		
Characteristic frequency of Rxn phonon bath	γ_0	200 cm^{-1}
Friction parameter of Rxn phonon bath	η_0	0.1
Reorganization energy of the Rxn phonon bath	λ_0	$\eta_0 M \gamma_0 \omega_{\mathrm{b}}/2$ (≈ 86.1 cm^{-1})
Spectator bath spectral density	$J_{\mathrm{Q}}(\omega)$	$2\lambda_{\mathrm{Q}}\gamma_{\mathrm{Q}}\omega/(\omega^2 + \gamma_{\mathrm{Q}}^2)$
Characteristic frequency of Spectator phonon bath	γ_0	6000 cm^{-1}
Reorganization energy of Spectator phonon bath	λ_0	0.15 cm^{-1}
Effective bath spectral density	$J_{\mathrm{eff}}(\omega)$	Eq. S8
Reorganization of the effective bath	λ_{eff}	$\eta_{\mathrm{c}}^2 \omega_{\mathrm{c}}$
Cavity lifetime	τ_{c}	500 fs

Supporting Information 2: Exact Quantum Dynamics using Hierarchical Equations of Motion

The Liouville space hierarchical equations of motion (HEOM) describe the reduced dynamics of the system linearly coupled to a Gaussian environment, which can be derived either from the standard path-integral formalism[9? –11] or the stochastic field method.[12,13] Yan put forward the concept of *dissipatons*, a kind of statistical quasi-particle, to identify the physical meanings of all dynamical quantities.[14–16] The corresponding formalism of quantum dynamics is characterized by the dissipaton equations of motion (DEOM). The total system-plus-bath composite Hamiltonian of an open quantum system reads as

$$\hat{H} = \hat{H}_{\rm S} + \hat{h}_{\rm B} + \hat{H}_{\rm SB}, \tag{S14}$$

where our model system for VSC (Eq. 17-19 of the main text) also belongs to this form. For arbitrary linear system-bath coupling with $\mathcal{M}$ modes,

$$\hat{H}_{\rm SB} = \sum_{a=1}^{\mathcal{M}} \hat{Q}_a \otimes \hat{F}_a. \tag{S15}$$

A. A brief introduction to *dissipaton*

The concept of dissipaton is capable to characterize the influence of bulk environments with only a few of them.[14,15] They arise strictly from the linear bath coupling components

$$\hat{F}_a = \sum_{k=1}^{K} \hat{f}_{ak}, \tag{S16}$$

with single-damping parameters given by

$$\langle \hat{f}_{ak}(t)\hat{f}_{bj}(0)\rangle_{\rm B} = \delta_{kj}\eta_{abk}e^{-\gamma_{ak}t}, \tag{S17a}$$

$$\langle \hat{f}_{bj}(0)\hat{f}_{ak}(t)\rangle_{\rm B} = \delta_{kj}\eta^{*}_{ab\bar{k}}e^{-\gamma_{ak}t}. \tag{S17b}$$

The associated index $\bar{k}$ in Eq. S17b is defined as $\gamma_{a\bar{k}} = \gamma_{ak}^{*}$. Eq. S16 and S17 lead to the bare-bath time-correlation function (TCF) expression as below,

$$C_{ab}(t) \equiv \langle \hat{F}_a(t)\hat{F}_b(0)\rangle_{\rm B} = \sum_{k=1}^{K} \eta_{abk} e^{-\gamma_{ak} t}, \tag{S18}$$

and its complex conjugation.

The dynamical variables in DEOM are the dissipaton density operators (DDOs), defined as follows,

$$\rho_{\mathbf{n}}^{(n)}(t) \equiv {\rm Tr}_{\rm B}\left[\Big(\prod_{ak} \hat{f}_{ak}^{n_{ak}}\Big)^{\circ} \hat{\rho}_{\rm T}(t)\right], \tag{S19}$$

where $\hat{\rho}_{\rm T}(t)$ is the time-dependent total density matrix, the product of dissipatons inside $(\cdots)^{\circ}$ means *irreducible.* And (c-number)$^{\circ}$ = 0. Bosonic dissipatons are symmetric under permutation, $(\hat{f}_{ak}\hat{f}_{bj})^{\circ} = (\hat{f}_{bj}\hat{f}_{ak})^{\circ}$. Each DDO in Eq. S19 represents a specific configuration of $\mathbf{n} \equiv \{\cdots, n_{ak}, \cdots \,|\, a = 1, \cdots, \mathcal{M};\ k = 1, \cdots, K\}$, with $n = \sum_{ak} n_{ak}$ dissipatons in total (*i.e.*, the number of tiers). We also denote that the associated DDO's index $\mathbf{n}_{ak}^{\pm}$ differs from $\mathbf{n}$ at the specified n_{ak} by ± 1, which means n_{ak} is replaced by $n_{ak} \pm 1$. The system reduced density operator is just the zeroth order DDO, $\hat{\rho}_{\rm S}(t) \equiv \rho_{\mathbf{n}=\mathbf{0}}^{(0)}(t)$.

The DEOM formalism can be constructed according to the dissipaton algebras, which include the *generalized diffusion equation* and *generalized Wick's theorem.* The generalized diffusion equation arises from the single-damping character in Eq. S17, which is

$${\rm Tr}_{\rm B}\left[\left(\frac{\partial \hat{f}_{ak}}{\partial t}\right)_{\rm B} \hat{\rho}_{\rm T}(t)\right] = -\gamma_{ak}{\rm Tr}_{\rm B}\left[\hat{f}_{ak}\hat{\rho}_{\rm T}(t)\right]. \tag{S20}$$

The generalized diffusion equation is applicable for the $h_{\rm B}$-action

$$\begin{aligned}\rho_{\mathbf{n}}^{(n)}(t; h_{\rm B}^{\times}) &\equiv {\rm Tr}_{\rm B}\left\{\Big(\prod_{ak} \hat{f}_{ak}^{n_{ak}}\Big)^{\circ}[\hat{h}_{\rm B}, \hat{\rho}_{\rm T}(t)]\right\} = {\rm Tr}_{\rm B}\left\{\Big[\Big(\prod_{ak} \hat{f}_{ak}^{n_{ak}}\Big)^{\circ}, \hat{h}_{\rm B}\Big]\ \hat{\rho}_{\rm T}(t)\right\} \\ &= -i\left(\sum_{ak} n_{ak}\gamma_{ak}\right)\rho_{\mathbf{n}}^{(n)}(t),\end{aligned} \tag{S21}$$

where $h_{\rm B}^{\times}\,\cdot \equiv [\hat{h}_{\rm B},\ \cdot]$. The second equality of Eq. S21 arises from the equivalence between the Schrödinger and Heisenberg prescription, and the last line arises from the Heisenberg equations of motion, $(\partial \hat{f}_{ak}/\partial t)_{\rm B} = -i[\hat{f}_{ak}, \hat{h}_{\rm B}]$. Eq. S21 summarizes the contribution by the bath Hamiltonian to the DDOs dynamics.[14,15] The generalized Wick's theorem deals with the system-hybrid-bath interaction, reading as

$$\mathrm{Tr}_{\rm B}\left[\Big(\prod_{ak}\hat{f}_{ak}^{n_{ak}}\Big)^{\circ}\hat{f}_{bj}\hat{\rho}_{\rm T}(t)\right] = \rho^{(n+1)}_{\mathbf{n}^{+}_{bj}}(t) + \sum_{ak} n_{ak}\langle \hat{f}_{ak}\hat{f}_{bj}\rangle^{>}_{\rm B}\rho^{(n-1)}_{\mathbf{n}^{-}_{ak}}(t), \tag{S22a}$$

$$\mathrm{Tr}_{\rm B}\left[\Big(\prod_{ak}\hat{f}_{ak}^{n_{ak}}\Big)^{\circ}\hat{\rho}_{\rm T}(t)\hat{f}_{bj}\right] = \rho^{(n+1)}_{\mathbf{n}^{+}_{bj}}(t) + \sum_{ak} n_{ak}\langle \hat{f}_{ak}\hat{f}_{bj}\rangle^{<}_{\rm B}\rho^{(n-1)}_{\mathbf{n}^{-}_{ak}}(t). \tag{S22b}$$

They will be used in evaluating the commutator action of linear system-bath coupling terms. The bosonic DEOM formalism is now readily to be constructed, reading as[14,15]

$$\begin{aligned}\dot{\rho}^{(n)}_{\mathbf{n}}(t) = &-\Big(i\mathcal{L}_{\rm S} + \sum_{ak} n_{ak}\gamma_{ak}\Big)\rho^{(n)}_{\mathbf{n}}(t) - i\sum_{ak}\mathcal{Q}_a^{\times}\rho^{(n+1)}_{\mathbf{n}^{+}_{ak}}(t)\\ &- i\sum_{abk} n_{ak}\Big(\eta'_{abk}\mathcal{Q}_b^{\times} + i\eta''_{abk}\mathcal{Q}_b^{\circ}\Big)\rho^{(n-1)}_{\mathbf{n}^{-}_{ak}}(t).\end{aligned} \tag{S23}$$

The involved superoperators and coefficients are defined as below:

$$\mathcal{L}_{\rm S}\hat{O} \equiv [\hat{H}_{\rm S}, \hat{O}], \qquad \mathcal{Q}_a^{\times}\hat{O} \equiv [\hat{Q}_a, \hat{O}], \qquad \mathcal{Q}_a^{\circ}\hat{O} \equiv \{\hat{Q}_a, \hat{O}\},$$
$$\eta'_{abk} \equiv \frac{\eta_{abk} + \eta^{*}_{ab\bar{k}}}{2}, \qquad \eta''_{abk} \equiv \frac{\eta_{abk} - \eta^{*}_{ab\bar{k}}}{2i}.$$

B. Bath TCF decomposition schemes

The central problem in HEOM/DEOM is to decompose the bare-bath TCF into a sum of exponential series, as is shown in Eq. S18. Based on the bosonic fluctuation-dissipation theorem (FDT),[17]

$$C_{ab}(t) = \frac{1}{\pi}\int_{-\infty}^{+\infty} d\omega\; \frac{e^{-i\omega t}J_{ab}(\omega)}{1 - e^{-\beta\omega}}, \tag{S24}$$

the decomposition can be realized by expanding-over-pole strategies with respect to the Bose-Einstein distribution function, such as Matsubara spectral decomposition (MSD)[17] and Padé spectral decomposition (PSD),[18–20] or directly by various least-square fitting schemes.[21–25] The tradi-

tional expanding-over-poles strategies are usually restricted to certain forms of bath spectral density functions.

In this work, the molecular phonon bath is assumed to be described by the Drude-Lorentz spectral density function (Eq. S3), so that its TCF decomposition can be achieved by PSD scheme. Here, we use the $[N-1/N]$ scheme[20] with three low-temperature correction terms. The cavity mode-photon-loss bath is described by the effective spectral density function $J_{\text{eff}}^{(1)}(\omega)$ (Eq. S8), which includes the effect of the cavity mode as well as its associated photon loss, is also treated by PSD with $[N-1/N]$ scheme and four low-temperature correction terms when the photon-loss bath is Markovian (so that is $J_{\text{eff}}^{(1)}(\omega)$ a Brownian).

C. Details of the HEOM Calculations

The quantum dynamics simulations are performed using the model Hamiltonian in Eq. S1 (Supplementary Note 1), where the double well and the spectator $\mathcal{Q}$ mode states are explicitly simulated and the cavity mode and its loss are incorporated into the bath. We use the basis $\{|\nu_i\rangle \otimes |n\rangle\}$ to describe the R_0 and $\mathcal{Q}$ DOF, where $|\nu_i\rangle$ is the eigenvector of $V(\hat{R}_0)$, and $|n\rangle$ is the Fock state for the harmonic $\mathcal{Q}$ mode. The influence of q_{c}, and its loss dynamics due to $\hat{H}_{\text{loss}}$ are treated using the effective spectral density description in Eq. S8. The influence from phonon bath $\hat{H}_{\nu}$ is treated using spectral density described in Eq. S13.

For practical reasons, the matter states $\{|\nu_i\rangle\}$ are truncated to confine the dynamics to the low-energy subspace. For models 1 and 2 (symmetrical double well), we used a total of $\mathcal{F}_0 = 5$ states for the double well and $\mathcal{F}_{\text{Q}} = 3$ states for the $\mathcal{Q}$ mode, leading to a total of $\mathcal{F} = \mathcal{F}_0 \cdot \mathcal{F}_{\text{Q}} = 15$ vibrational states. For model 3 (asymmetrical double well), we use $\mathcal{F}_0 = 6$ and $\mathcal{F}_{\text{Q}} = 3$. The $\hat{H}_{\text{B}} + \hat{H}_{\text{SB}}$ terms (see Eq. S10b) are the bath degrees of freedom (DOF) and the system-bath couplings, which are propagated based on the HEOM formalism with the spectral densities described in Eq. S13 for the thermal baths of the reaction coordinate and $\mathcal{Q}$ mode and Eq. S8 for the cavity mode. The propagation used the fourth order Runge-Kutta (RK-4) integrator with a 0.05 fs time step, along with the on-the-fly filtering algorithm with an error tolerance of 1×10^{-7}.

D. Infrared Spectra of the Bare-molecular System

The infrared (IR) spectra can be directly computed from the Fourier transform of the dipole-dipole correlation function according to[8]

$$\mathcal{A}(\omega) \propto \int_0^\infty dt \, \langle \hat{\mu}(t)\hat{\mu}(0)\rangle e^{-i\omega t}, \tag{S25}$$

where the dipole-dipole correlation function is defined as

$$\langle \hat{\mu}(t)\hat{\mu}(0)\rangle = \mathrm{Tr}\left[\hat{\mu}(t)\hat{\mu}(0)\hat{\rho}_{\mathrm{S}}(0)\right], \tag{S26}$$

which is obtained using the HEOM steady-state solver.[26,27] The dipole operator is described as $\hat{\mu} = \mu_0' \hat{R}$ for our model (linear dipole model). The IR spectra intensity is normalized in Fig. 3b of the main text. Here, we assume that the reaction coordinate R_0 does not carry any transition dipole. However, due to the coupling between R_0 and $\mathcal{Q}$, with a large enough coupling strength $\mathcal{C}_{\mathcal{Q}}$, it will eventually cause a peak splitting and shows a double peak for IR spectra (due to the intensity borrowing from $\mathcal{Q}$ mode). In Ref. 28, it was stated that "The cavity is tuned so that it is resonant with the stretching transitions of the C-Si bond around 860 cm^{-1}, a double peak corresponding to the C-C-Si and Si-(Me)$_3$ modes" and indeed found the splitting of the IR spectra of the molecule outside the cavity.

Supporting Information 3: Rate Constant Calculations

A factorizable initial condition between the system and bath subspaces is assumed and used in the HEOM simulations. Here, we assume an initial density matrix

$$\hat{\rho}(0) = |\nu_{\mathrm{L}}\rangle\langle\nu_{\mathrm{L}}| \otimes \frac{e^{-\beta\hat{H}_{\mathcal{Q}}}}{Z_{\mathcal{Q}}} \otimes \frac{e^{-\beta\hat{H}_{\mathrm{B}}}}{Z_{\mathrm{B}}}, \tag{S27}$$

where $Z_{\mathcal{Q}} \equiv \mathrm{Tr}_{\mathcal{Q}}[e^{-\beta\hat{H}_{\mathcal{Q}}}]$ and $Z_{\mathrm{B}} \equiv \mathrm{Tr}_{\mathrm{B}}[e^{-\beta\hat{H}_{\mathrm{B}}}]$ are partition functions associated with the spectator modes $\{\mathcal{Q}_j\}$ and the bath $\hat{H}_{\nu}$, respectively. Further, $\mathrm{Tr}_k[\cdot]$ denotes the partial trace over the k_{th} DOF. Eq. S27 is the initial condition we adopt for all the numerical simulations, including the rate constant and the population dynamics.

The reduced density matrix associate with R_0 (the reaction coordinate) is given by

$$\hat{\rho}_0 = \mathrm{Tr}_{\mathrm{B}}\,\mathrm{Tr}_{\mathcal{Q}}[\hat{\rho}(t)], \tag{S28}$$

where $\hat{\rho}(t)$ is the density matrix at time t. The time-dependent populations of reactants ($\mathcal{R}$) and products ($\mathcal{P}$) are computed as

$$P_{\mathcal{R}}(t) = \mathrm{Tr}_0[(1-\hat{h})\hat{\rho}_0(t)], \tag{S29a}$$

$$P_{\mathcal{P}}(t) = 1 - P_{\mathcal{R}}(t), \tag{S29b}$$

where $\mathrm{Tr}_0[\cdot]$ as the trace over the R_0 DOF, $\hat{h} = h(\hat{R}_0 - R^{\ddagger})$ is the Heaviside operator which projects onto the product states, having the values $h(R) = 1$ for $R > R^{\ddagger}$ and $h(R) = 0$ for $R < R^{\ddagger}$, $R^{\ddagger}$ is the position of the diving surface which is placed at $R^{\ddagger} = 0$ for the model considered here. Finally, the forward rate constant is calculated as[2,4,29]

$$k = -\lim_{t\to t_p} \frac{\dot{P}_{\mathcal{R}}(t)}{P_{\mathcal{R}}(t) + \chi_{\mathrm{eq}} \cdot [P_{\mathcal{R}}(t) - 1]}, \tag{S30}$$

where t_{p} is the plateau time under which the function in Eq. S30 plateaus. In addition, $\chi_{\mathrm{eq}} = P_R/P_P$ is the ratio of the reactant and product population at equilibrium that for the symmetric double well considered in this work is equal to 1 and $\dot{P}_R(t)$ is the time derivative of the reactant population

which is evaluated numerically. Examples of Eq. S30 under different conditions are presented in Fig. S1.

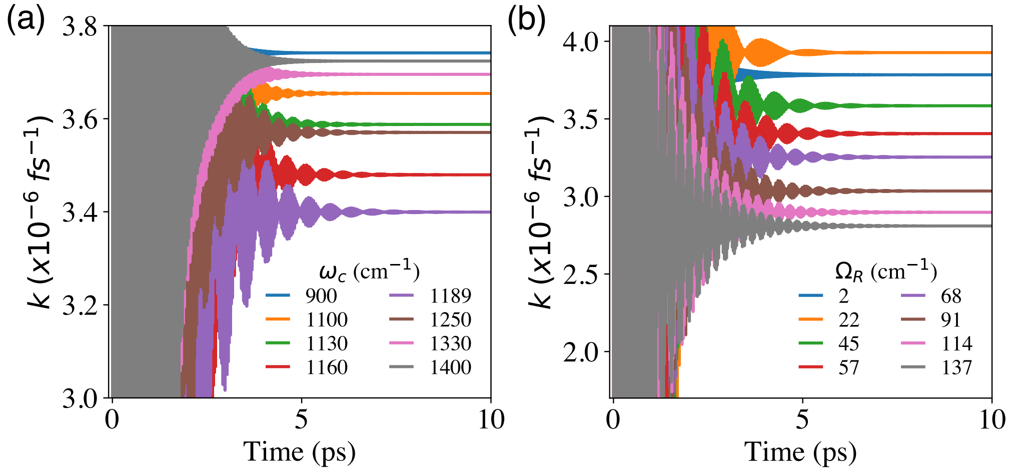

Figure S1: Flux-side correlation function calculated using Eq. S30 for **(a)** various cavity frequencies at $\Omega_\mathrm{R} \approx 60$ cm^{-1} and **(b)** Rabi splitting at resonance ($\omega_\mathrm{c} = \omega_\mathrm{Q}$). For both plots, the cavity lifetime is $\tau_\mathrm{c} = 500$ fs.

Supporting Information 4: Population Dynamics and Kinetics Analysis

A. Population Dynamics Analysis for Resonance Suppression

Fig. S2 shows the population dynamics of Model 1 (see Supplemental Note 1), with the vibrational diabatic states $|\nu'_\mathrm{L}\rangle$ (green), $|\nu'_\mathrm{R}\rangle$ (red) and $|\nu_\mathrm{R}\rangle$ (blue). The higher energy eigenstate, such as $|\nu_4\rangle$ (gray) are not significantly populated. Both outside the cavity (dashed lines) and inside a resonant cavity ($\omega_\mathrm{c} = \omega_\mathrm{Q}$) (solid) are presented, with $|\nu_\mathrm{L}\rangle$ as the initial populated state. The cavity lifetime is taken as $\tau_\mathrm{c} = 500$ fs and light-matter coupling $\eta_\mathrm{c} = 0.005$ which corresponds to a Rabi splitting of $\Omega_\mathrm{R} \approx 100\ \mathrm{cm}^{-1}$. The short-time dynamics in Fig. S2b indicates that there is an initial rise of the population of the $|\nu'_\mathrm{L}\rangle$ state followed by the $|\nu'_\mathrm{R}\rangle$ state, through the tunneling induced by the Δ' coupling, which finally relaxes and accumulates on the product state $|\nu_\mathrm{R}\rangle$. We note that there is also a population transfer to the higher order $|\nu_4\rangle$ state. However, this is negligible (compared to the other channel) and rapidly decays to zero, and could be ignored. Thus, the reaction mechanism for both the inside and outside cavity scenarios is represented as follows

$$|\nu_\mathrm{L}\rangle \xrightarrow{k_1} |\nu'_\mathrm{L}\rangle \xrightarrow{k_2} |\nu'_\mathrm{R}\rangle \xrightarrow{k_3} |\nu_\mathrm{R}\rangle \tag{S31}$$

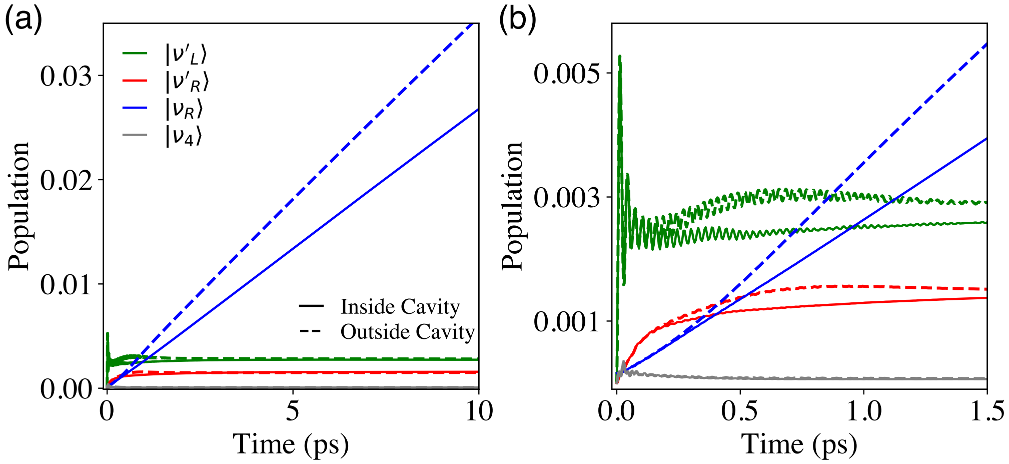

Figure S2: Population dynamics of the product state and the first 3 excited states of the reactive model at **(a)** long and **(b)** short times.

The steady-state behavior of $|\nu'_\mathrm{L}\rangle$ indicates that $k_1 \ll k_2$, such that the rate constant of the entire reaction can be approximated as $k_0 \approx k_1$ for outside the cavity case as well as $k \approx k_1$ for

inside the cavity case. The detailed discussion can be found in Ref. 5. This figure also shows that the cavity coupling effect is to decrease the steady state population of $|\nu'_{\rm L}\rangle$ (green).

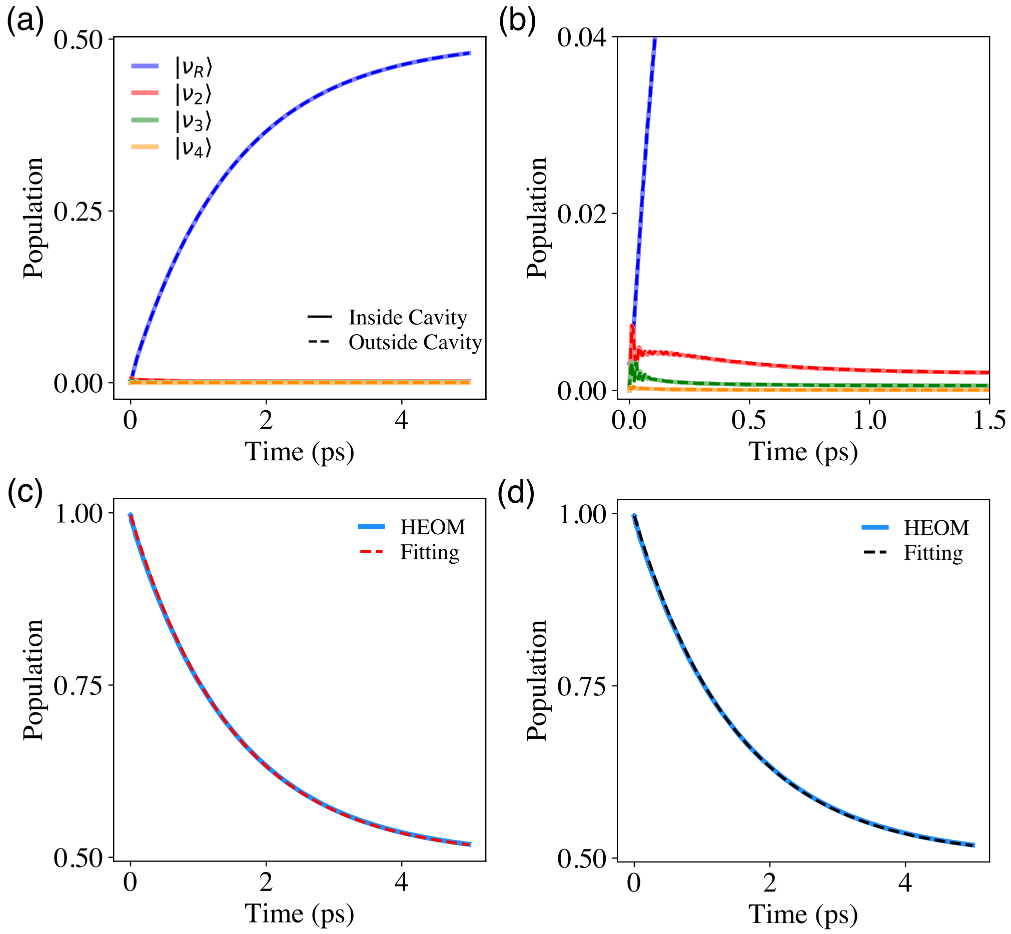

Figure S3: (**a**). Population dynamics of the first 5 double well states for model II (null VSC effect). (**b**) The short-time zoom-in of (**a**) during the first 1.5 ps. Example of the exponential decay fitting of the reactant state population ($P_{\mathcal{R}}$) for (**c**) $\Omega_{\rm R} = 60$ cm^{-1}, $\omega_{\rm c} = 1200$ cm^{-1} and (**d**) $\Omega_{\rm R} = 100$ cm^{-1}, $\omega_{\rm c} = \omega_{\rm Q} = 1253$ cm^{-1}. In all cases, the cavity lifetime is set to be $\tau_{\rm c} = 500$ fs.

B. Population Dynamics for Null VSC effect

In Fig. S3, the population dynamics of the null reactive model (model 2 in Supplemental note 1) are presented for the product state $|\nu_{\mathrm{R}}\rangle$ and the first 3 vibrationally excited eigenstates, for outside the cavity case (dashed lines), as well as coupled inside a resonant cavity (solid lines) under $\omega_{\mathrm{c}} = \omega_{\mathrm{Q}}$ with a light-matter coupling $\eta_{\mathrm{c}} = 0.005$ (corresponding to a Rabi splitting $\Omega_{\mathrm{R}} \approx 100$ cm^{-1}) and a cavity lifetime $\tau_{\mathrm{c}} = 500$ fs. The short-time dynamics in Fig. S3b shows that after some initial transient dynamics, the population of the excited vibration states stays constant throughout at a longer time. The direct tunneling from the reactant to the product state ($|\nu_{\mathrm{L}}\rangle \rightarrow |\nu_{\mathrm{R}}\rangle$) is the main pathway by which the reaction proceeds.

To obtain the rate constant for this model, we fitted the population dynamics according to[30]

$$P_{\mathrm{R}}(t') = k\tilde{P}_{\mathrm{L}}(t') - k_{\mathrm{b}}\tilde{P}_{\mathrm{R}}(t') \quad \text{(S32)}$$

where $\tilde{P}_{\mathcal{L}}(t') = \int_0^{t'} P_{\mathrm{L}}(t) \cdot dt$ and $\tilde{P}_{\mathcal{R}}(t') = \int_0^{t'} P_{\mathrm{R}}(t) \cdot dt$. In the above equation, k and k_{b} are the forward and backward rate constant, respectively. Further, L and R refer to reactants and products, which are defined as the population on the left and right sides of a dividing surface placed at $R_0 = 0$. After obtaining the populations P_{L} and P_{R} from the HEOM simulations, we integrated them to obtain $\tilde{P}_{\mathrm{L}}(t')$ and $\tilde{P}_{\mathrm{R}}(t')$, finally, Eq. S32 was fitted to get k and k_{b} in a range of time $[0, t_{\mathrm{f}}]$. Here we use $t_{\mathrm{f}} = 5$ ps. The comparison between the numerical simulations and the fitting to Eq. S32 are provided in Fig. S3, with the rate constants results provided in Fig. 4 of the main text.

C. Population Dynamics and Rate Constants for an Asymmetrical Double-Well Model Coupled to the Cavity

To verify our proposed mechanism in Eq. 6 of the main text is general and regardless of the symmetry of the double well potential,[4] we have performed additional simulations with an asymmetrical double well potential. Note that a similar test for q_{c} directly coupled to R_0 (that leads to resonance enhancement) has already been performed in Ref. 4 (see Fig. S4 in Supplementary Note 4 of that work).

Fig. S4**a** presents the asymmetric double-well potential as a function of the reaction coordinate

R_0, with detailed model parameters have been provided in Table. S3. We refer to it as Model 3. On top of the potential, we visualize six vibrational eigenstates of $V(R_0)$, which we label based on their energy (from low to high) as $|\nu_0\rangle$, $|\nu_1\rangle$, ..., $|\nu_5\rangle$. Due to the asymmetry of the potential, some of these eigenstates are localized in the left well ($|\nu_1\rangle$) or in the right well ($|\nu_0\rangle$ and $|\nu_2\rangle$). Other states are delocalized vibrational excited states ($|\nu_3\rangle$, $|\nu_4\rangle$, and $|\nu_5\rangle$). We set $|\nu_1\rangle$ as the initially populated state because it is the only localized eigenstate in the left well and can be viewed as the reactant ground state. In addition, a spectator mode with frequency ω_Q matches the $|\nu_1\rangle \rightarrow |\nu_3\rangle$ transition frequency ($\omega_\mathrm{Q} = (E_3 - E_1)/\hbar \approx 1175$ cm^{-1}) is coupled to R_0, with the expression in Eq. 2 of the main text.

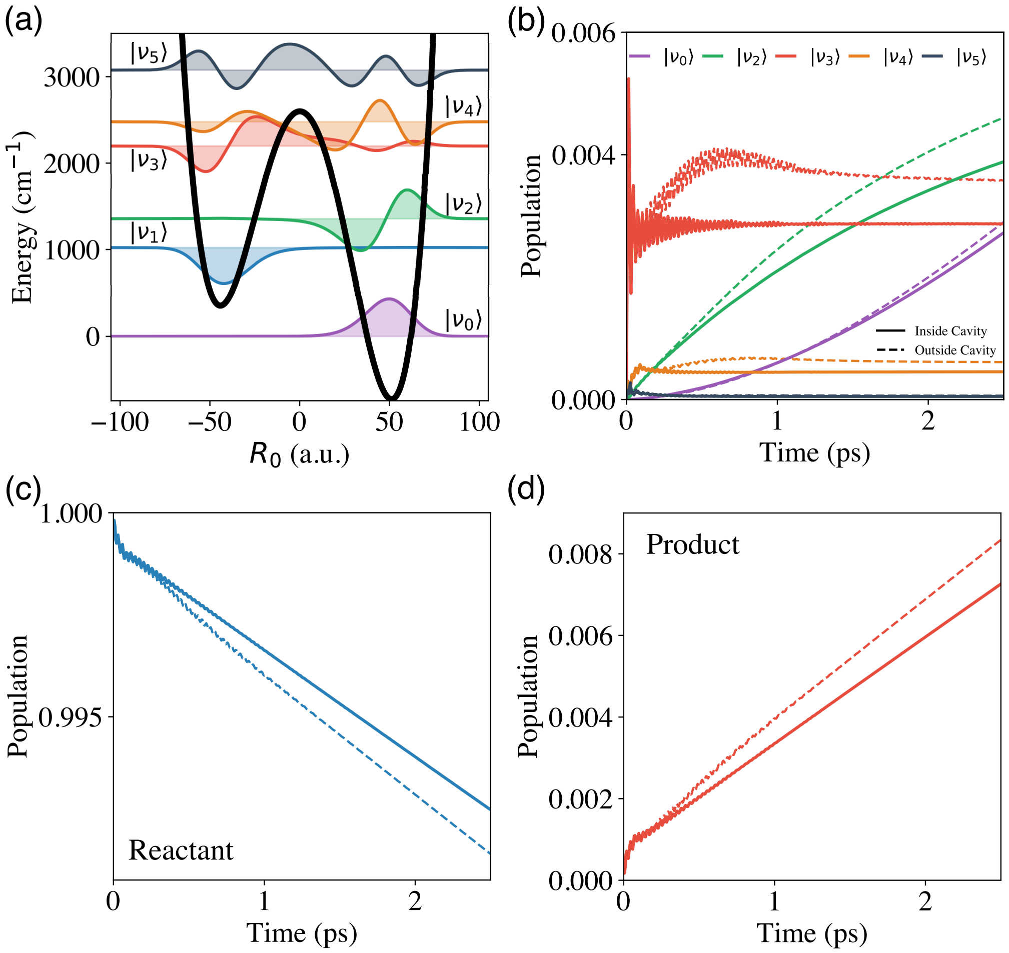

Figure S4: (**a, b**). Population dynamics of 5 asymmetric double well vibrational states for model III. Population dynamics of (**c**) reactants and (**d**) products are defined as the population at the left and right sides of a dividing surface placed at $R_0 = 0$, respectively. Here $\omega_c = \omega_\mathrm{Q} = 1175$ cm^{-1}, $\Omega_\mathrm{R} = 180$ cm^{-1} and $\tau_c = 500$ fs.

Fig. S4**b** presents the population dynamics obtained using HEOM, for the case outside the cavity (dashed lines) and inside the cavity (solid lines) when resonantly couple q_c with $\mathcal{Q}$ mode under $\omega_c = \omega_Q$ and light-matter coupling strength $\Omega_R = 180$ cm^{-1}. The color coding for each state is consistent with the vibrational states presented in Fig S4**a**. The population dynamics shows an initial population transfer from $|\nu_1\rangle$ to $|\nu_3\rangle$ and to $|\nu_4\rangle$, followed by a relaxation to $|\nu_2\rangle$, and finally accumulates on $|\nu_0\rangle$. There is a population transfer to the higher energy state $|\nu_5\rangle$; however, its occupation is negligible (compared to the other states) and quickly decays to zero.

Fig. S4**c-d** further presents the population of reactant and product, defined in Eq. S29, for outside the cavity case (dashed line) and coupled inside the cavity (solid lines). The dividing surface is placed at $\hat{R}_0 = R^\ddagger = 0$. As expected, coupling to the resonant cavity reduces the product population and rate constant (which is approximately the slope of the product population).

To compute the rate constant for the asymmetrical double well case, additional basis and tiers in HEOM simulations are required to fully converge the flux-side correlation function (Eq. S30). Therefore, we opted to fit the population dynamics according to Eq. S32 (same fitting procedure as used for the Null VSC result case). Here we use $t_f = 3$ ps.

To apply our FGR theory to calculate the VSC suppression of the rate constant, we need to construct diabatic states that are localized on the left and right wells. To this end, we perform diabatization of state $|\nu_3\rangle$ and $|\nu_4\rangle$. We defined the diabatic states

$$\begin{bmatrix} |\nu'_L\rangle \\ |\nu'_R\rangle \end{bmatrix} = \begin{bmatrix} c_3 & c_4 \\ \tilde{c}_3 & \tilde{c}_4 \end{bmatrix} \begin{bmatrix} |\nu_3\rangle \\ |\nu_4\rangle \end{bmatrix} \tag{S33a}$$

$$\left|\nu'_L\right\rangle = c_3 \left|\nu_3\right\rangle + c_4 \left|\nu_4\right\rangle \qquad \left|\nu'_R\right\rangle = \tilde{c}_3 \left|\nu_3\right\rangle + \tilde{c}_4 \left|\nu_4\right\rangle , \tag{S33b}$$

where the coefficients are chosen to maximize the following overlaps[31,32]

$$\begin{aligned} \int_{-\infty}^{R^\ddagger} \langle \nu'_L|x\rangle\langle x|\nu'_L\rangle dx = |c_3|^2 \int_{-\infty}^{R^\ddagger} \langle \nu_3|x\rangle\langle x|\nu_3\rangle dx + c_3^* c_4 \int_{-\infty}^{R^\ddagger} \langle \nu_3|x\rangle\langle x|\nu_4\rangle dx \\ + c_4^* c_3 \int_{-\infty}^{R^\ddagger} \langle \nu_4|x\rangle\langle x|\nu_3\rangle dx + |c_4|^2 \int_{-\infty}^{R^\ddagger} \langle \nu_4|x\rangle\langle x|\nu_4\rangle dx \end{aligned} \tag{S34a}$$

$$\int_{R^\ddagger}^{\infty} \langle \nu'_{\rm R}|x\rangle\langle x|\nu'_{\rm R}\rangle dx = |\tilde{c}_3|^2 \int_{R^\ddagger}^{\infty} \langle \nu_3|x\rangle\langle x|\nu_3\rangle dx + \tilde{c}_3^*\tilde{c}_4 \int_{R^\ddagger}^{\infty} \langle \nu_3|x\rangle\langle x|\nu_4\rangle dx + \tilde{c}_4^*\tilde{c}_3 \int_{R^\ddagger}^{\infty} \langle \nu_4|x\rangle\langle x|\nu_3\rangle dx + |\tilde{c}_4|^2 \int_{R^\ddagger}^{\infty} \langle \nu_4|x\rangle\langle x|\nu_4\rangle dx \quad \text{(S34b)}$$

while satisfying the constraints $\langle \nu'_{\rm L}|\nu'_{\rm L}\rangle = 1$, $\langle \nu'_{\rm R}|\nu'_{\rm R}\rangle = 1$, and $\langle \nu'_{\rm L}|\nu'_{\rm R}\rangle = 0$. This way of choosing the diabats maximizes the amplitude of $|\nu'_{\rm L}\rangle$ and $|\nu'_{\rm R}\rangle$ on the left and right side of the well, respectively. A similar procedure can also be performed for $|\nu_1\rangle$ and $|\nu_2\rangle$, which will generate nearly identical diabatic states as the original vibrational eigenstates, indicating that $|\nu_1\rangle$ and $|\nu_2\rangle$ are already very localized. The constrained optimization in Eq. S34 is performed using the Lagrangian multiplier method implemented in the optimize.minimize function of SciPy, resulting in

$$\begin{bmatrix} |\nu'_{\rm L}\rangle \\ |\nu'_{\rm R}\rangle \end{bmatrix} = \begin{bmatrix} 0.969 & 0.247 \\ -0.247 & 0.969 \end{bmatrix} \begin{bmatrix} |\nu_3\rangle \\ |\nu_4\rangle \end{bmatrix} \quad \text{(S35)}$$

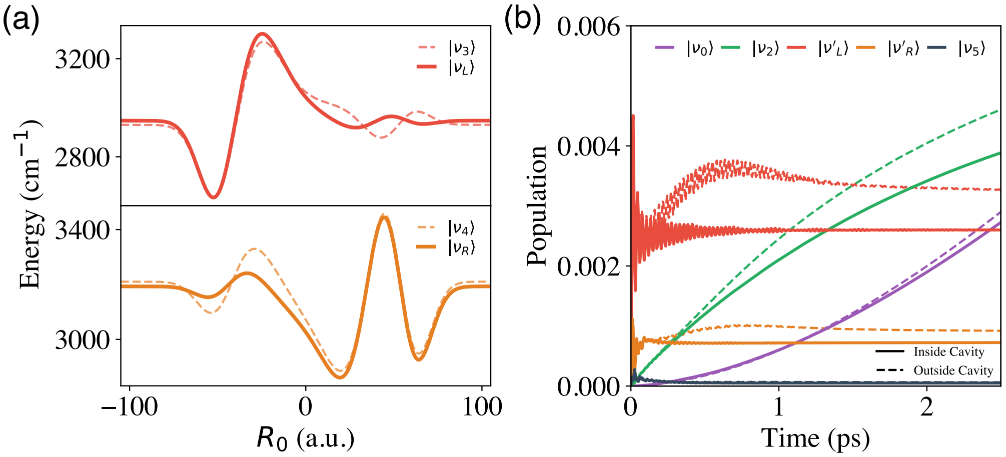

Figure S5: (**a**) Comparison between the adiabatic ($|\nu_3\rangle$, $|\nu_4\rangle$) and diabatic states ($|\nu'_{\rm L}\rangle$, $|\nu'_{\rm R}\rangle$) obtained through Eq. S34. (**b**) Population dynamics of 5 asymmetric double well vibrational states, the diabatic states ($|\nu'_{\rm L}\rangle$, $|\nu'_{\rm R}\rangle$) are considered. Here $\omega_c = \omega_{\rm Q} = 1175$ cm^{-1}, $\Omega_{\rm R} = 180$ cm^{-1} and $\tau_c = 500$ fs

Fig. S5(**a**) presents the comparison between the adiabatic ($|\nu_3\rangle$, $|\nu_4\rangle$) and diabatic states ($|\nu'_{\rm L}\rangle$, $|\nu'_{\rm R}\rangle$). As expected, $|\nu'_{\rm L}\rangle$ and $|\nu'_{\rm R}\rangle$ closely resembles the $|\nu_3\rangle$ and $|\nu_4\rangle$ states, respectively, but becomes more localized. Fig. S5(**b**) presents the population dynamics for the outside cavity (dashed lines) and inside cavity (solid lines) cases in the diabatic representation of $|\nu'_{\rm L}\rangle$ (red) and $|\nu'_{\rm R}\rangle$ (orange). Based on the above discussions, the reaction mechanism of this asymmetrical double well model can

be summarized as

$$|\nu_1\rangle \xrightarrow{k_1} |\nu'_{\mathrm{L}}\rangle \xrightarrow{k_2} |\nu'_{\mathrm{R}}\rangle \xrightarrow{k_3} |\nu_2\rangle \xrightarrow{k_4} |\nu_0\rangle \tag{S36}$$

which is consistent with the case for the symmetrical double well presented in the main text. Further, we can observe that the populations for vibrationally excited diabatic states $|\nu'_{\mathrm{L}}\rangle$ and $|\nu'_{\mathrm{R}}\rangle$ exhibit the plateau population (steady state behavior) after the transient short time in Fig S4**b**. Thus, the assumption of k_1 being the rate-limiting step still holds. Then the overall rate constant can be approximated as

$$k \approx k_1 = k_{\mathrm{D}} + \alpha \cdot k_{\mathrm{VSC}}, \tag{S37}$$

where k_{D} is the rate constant for the double-well potential without coupling to any $\mathcal{Q}$ modes or the cavity mode q_{c}, and α is a scaling parameter. This means we can use our analytic k_{VSC} expression in Eq. 10 of the main text to estimate k/k_0 for the asymmetrical case.

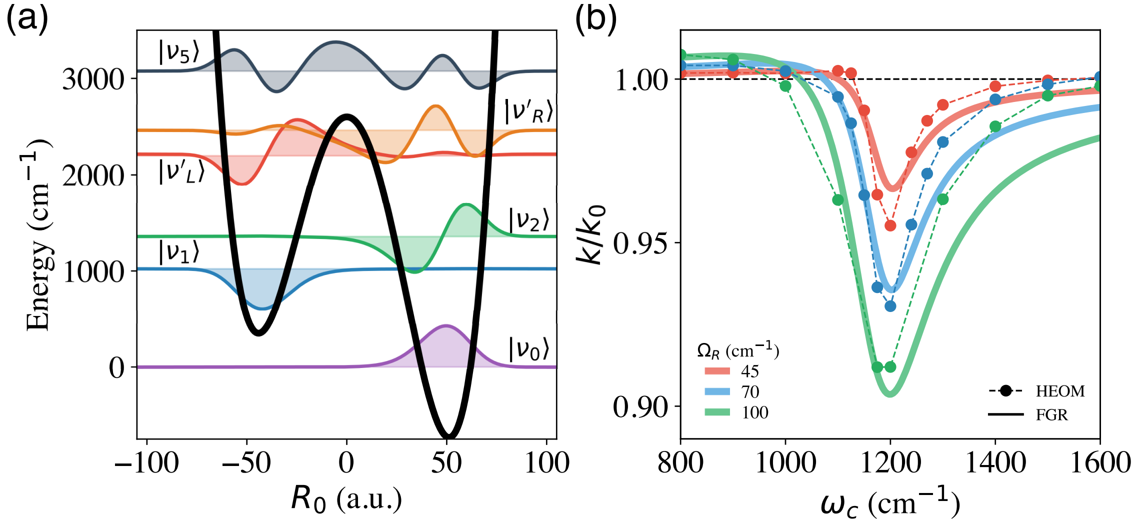

Figure S6: (**a**) Asymmetric double-well potential with six lowest vibrational states presented; states $|\nu_3\rangle$ and $|\nu_4\rangle$ are diabatized as $|\nu'_{\mathrm{L}}\rangle$ and $|\nu'_{\mathrm{R}}\rangle$. (**b**) Normalized rate constant k/k_0 (with respect to the outside cavity case) as a function of the cavity frequency ω_{c} at various Rabi splitting Ω_{R}. Here $\tau_c = 500$ fs and $\omega_{\mathrm{Q}} = 1175$ cm^{-1}.

We further use Eq. S37 to estimate the rate constant from our current FGR theory of k_{VSC} (Eq. 10 of the main text). Here, under the condition $\eta_{\mathrm{c}} = 0$ (outside the cavity), we find that $\alpha = 0.6$ will bring the FGR analytic results to quantitatively agree with the numerically exact results from HEOM. With the definition of diabatic states, the transition frequency is given by $\omega_0 = (E'_{\mathrm{L}} - E_1)/\hbar \approx 1192.16$ cm^{-1}, which is used in the FGR expression (Eq. 11 of the main text).

Fig. S6**b** presents the cavity frequency dependence of the normalized rate constant k/k_0, obtained from HEOM (dots) as well as from the current FGR theory (solid lines). For all three considered coupling strengths, the FGR expression provides a good quantitative agreement with the HEOM data, in terms of capturing the sharp resonance behavior and the width of the action spectrum. Compared to the symmetrical case (Fig. 2 of the main text), the deviation between FGR and HEOM becomes slightly large, likely due to the more complicated dynamics in the asymmetrical double well systems. On the other hand, the mechanism in Eq. S37, as well as the basic feature of k/k_0 is well captured by the current theory of k_{VSC}. We thus conclude that the VSC mechanism and FGR theory are not sensitive to the detailed shape of the double well potential, at least for the model systems we investigated here.

Supporting Information 5: Derivation of $J_{\text{eff}}(\omega)$

In this section, we derive the effective spectral density function in Eq. 9 of the main text. We will also derive the most general form of the spectral density, and various special cases with approximations to obtain closed-form analytic expressions.

A. The most general case

We start by considering the Hamiltonian which describes a single cavity mode coupling to many solvent DOF, which is expressed as follows[4,33]

$$\hat{H} = \hat{H}_{\text{M}} + \hat{H}_{\mathcal{Q}} + \hat{H}_{\text{LM}} + \hat{H}_{\nu} + \hat{H}_{\text{loss}}, \tag{S38}$$

Each term of the above Hamiltonian is expressed as

$$\hat{H}_{\text{M}} = \frac{\hat{P}_{\text{M}}^2}{2} + \hat{V}(\hat{R}_0), \tag{S39a}$$

$$\hat{H}_{\mathcal{Q}} = \sum_{j=1}^{N} \left[\frac{\hat{\Pi}_j^2}{2} + \frac{\omega_j^2}{2} \left(\hat{\mathcal{Q}}_j - \frac{\mathcal{C}_j}{\omega_j^2} \cdot \hat{R}_0 \right)^2 \right], \tag{S39b}$$

$$\hat{H}_{\text{LM}} = \frac{\hat{p}_{\text{c}}^2}{2} + \frac{\omega_{\text{c}}^2}{2} \left[\hat{q}_{\text{c}} + \sum_{j=1}^{N} \nu_j \cdot \hat{\mathcal{Q}}_j \right]^2, \tag{S39c}$$

$$\hat{H}_{\nu} = \sum_{j=1}^{N} \frac{1}{2} \sum_{m} \left[\hat{p}_{m,j}^2 + \omega_{m,j}^2 \left(\hat{x}_{m,j} - \frac{c_{m,j}}{\omega_{m,j}^2} \cdot \hat{\mathcal{Q}}_j \right)^2 \right], \tag{S39d}$$

$$\hat{H}_{\text{loss}} = \frac{1}{2} \sum_{n} \left[\hat{p}_n^2 + \omega_n^2 \left(\hat{x}_n - \frac{c_n}{\omega_n^2} \cdot \hat{q}_{\text{c}} \right)^2 \right], \tag{S39e}$$

where $\nu_j = \sqrt{\frac{2}{\omega_{\text{c}}}} \eta_j \cdot \cos\varphi_j$, and η_j is the light-matter coupling of spectator mode j. The reorganization energy defined in Eq. 3 of the main text should be generalized as

$$\Lambda = \sum_{j=1}^{N} \mathcal{C}_j^2 / (2\omega_j^2). \tag{S40}$$

The angle between the dipole operator associated with $\mathcal{Q}_j$ and the field polarization is φ_j. Note that the Hamiltonian above is of the most general form for a single mode case, which considers

R_0-$\{\mathcal{Q}_j\}$ coupling disorders described by $\{\mathcal{C}_j\}$, frequency disorders of the spectator modes $\{\mathcal{Q}_j\}$ described by $\{\omega_j\}$, as well as dipole orientation disorders described by $\{\eta_j\}$ and $\{\cos\varphi_j\}$.

Next, we will adopt the approach followed by Garg et al[6] and others.[34,35] We also note that the spectral density of this system can also be constructed by means of normal mode transformation,[33] which is, in principle equivalent to the results via harmonic analysis adopted in this section, and will be discussed later in Supplemental Note 7. Consequently, the EOMs for this system in Eq. S39 are expressed as

$$-\ddot{R}_0 = V'(R_0) - \sum_j \mathcal{C}_j \left(\mathcal{Q}_j - \frac{\mathcal{C}_j}{\omega_j^2} R_0\right), \tag{S41a}$$

$$-\ddot{\mathcal{Q}}_j = \omega_j^2 \left(\mathcal{Q}_j - \frac{\mathcal{C}_j}{\omega_j^2} R_0\right) + \omega_c^2 \nu_j \left(q_c + \sum_j \nu_j \mathcal{Q}_j\right) - \sum_m c_{m,j} \left(x_{m,j} - \frac{c_{m,j}}{\omega_{m,j}^2} \mathcal{Q}_j\right), \tag{S41b}$$

$$-\ddot{q}_c = \omega_c^2 \left(q_c + \sum_j \nu_j \mathcal{Q}_j\right) - \sum_n c_n \left(x_n - \frac{c_n}{\omega_n^2} q_c\right), \tag{S41c}$$

$$-\ddot{x}_{m,j} = \omega_{m,j}^2 \left(x_{m,j} - \frac{c_{m,j}}{\omega_{m,j}^2} \mathcal{Q}_j\right), \tag{S41d}$$

$$-\ddot{x}_n = \omega_n^2 \left(x_n - \frac{c_n}{\omega_n^2} q_c\right). \tag{S41e}$$

where $V'(R_0) = \partial_{R_0} V$ is the derivative of the potential. Performing Fourier transforms on the above equations, we have

$$-\tilde{V}'_\omega(\tilde{R}_0) = \left(\sum_j \frac{\mathcal{C}_j^2}{\omega_j^2} - \omega^2\right) \tilde{R}_0(\omega) + \sum_j \mathcal{C}_j \tilde{\mathcal{Q}}_j, \tag{S42a}$$

$$-\mathcal{C}_{j'} \tilde{R}_0 + \omega_c^2 \nu_{j'} \tilde{q}_c - \sum_m c_{m,j'} \tilde{x}_{m,j'} + \omega_c^2 \nu_{j'} \sum_j \nu_j \tilde{\mathcal{Q}}_j + \left(\omega_{j'}^2 - \omega^2 + \sum_m \frac{c_{m,j}^2}{\omega_{m,j}^2}\right) \tilde{\mathcal{Q}}_{j'} = 0, \tag{S42b}$$

$$\omega_c^2 \sum_j \nu_j \tilde{\mathcal{Q}}_j - \sum_n c_n \tilde{x}_n + \left(\omega_c^2 - \omega^2 + \sum_n \frac{c_n^2}{\omega_n^2}\right) \tilde{q}_c = 0, \tag{S42c}$$

$$\left(\omega_m^2 - \omega^2\right) \tilde{x}_{m,j} - c_{m,j} \tilde{\mathcal{Q}}_j = 0, \tag{S42d}$$

$$\left(\omega_n^2 - \omega^2\right) \tilde{x}_n - c_n \tilde{q}_c = 0. \tag{S42e}$$

where $\tilde{V}'_\omega(\tilde{R}_0)$ is the Fourier tranform of $V'(R_0)$. Solving Eq. S42d and Eq. S42e for $\tilde{x}_m$ and $\tilde{x}_n$, we

have

$$\tilde{x}_n(\omega) = \frac{c_n}{\omega_n^2 - \omega^2}\tilde{q}_{\rm c}, \qquad \tilde{x}_{m,j}(\omega) = \frac{c_{m,j}}{\omega_{m,j}^2 - \omega^2}\tilde{\mathcal{Q}}_j. \tag{S43}$$

Then, Eq S42c can be solved for $\tilde{q}_{\rm c}$, as follows

$$\tilde{q}_{\rm c} = \frac{-\omega_{\rm c}^2}{\omega_{\rm c}^2 + L(\omega)} \sum_j \nu_j \tilde{\mathcal{Q}}_j, \tag{S44}$$

with

$$L(\omega) = -\omega^2 \left[1 + \sum_n \frac{c_n^2}{\omega_n^2(\omega_n^2 - \omega^2)}\right]. \tag{S45}$$

Plugging in Eq. S43 and S44 into Eq. S42b, one has

$$[\omega_{j'}^2 + P_{j'}(\omega)]\tilde{\mathcal{Q}}_{j'} + \frac{\omega_{\rm c}^2 L(\omega)\sum_j \nu_j \tilde{\mathcal{Q}}_j}{\omega_{\rm c}^2 + L(\omega)}\nu_{j'} = \mathcal{C}_{j'}\tilde{R}_0, \tag{S46}$$

where we defined

$$P_j(\omega) = -\omega^2 \left[1 + \sum_m \frac{c_{m,j}^2}{\omega_{m,j}^2(\omega_{m,j}^2 - \omega^2)}\right]. \tag{S47}$$

According to the Caldeira-Leggett model, the baths as well as their coupling to the system can be described by spectral density functions, defined as follows,

$$J_{{\rm Q},j}(\omega) = \frac{\pi}{2}\sum_m \frac{c_{m,j}^2}{\omega_{m,j}}\delta(\omega - \omega_{m,j}) = \frac{2\lambda_{{\rm Q},j}\gamma_{{\rm Q},j}\omega}{\omega^2 + \gamma_{{\rm Q},j}^2}, \tag{S48}$$

$$J_{\rm loss}(\omega) = \frac{\pi}{2}\sum_n \frac{c_n^2}{\omega_n}\delta(\omega - \omega_n) = \frac{\omega}{\tau_{\rm c}}e^{-\omega/\omega_{\rm m}}, \tag{S49}$$

where $J_{{\rm Q},j}(\omega)$ are the spectral densities of the spectator modes' local baths which are assumed to have the Drude-Lorentz form, and $J_{\rm loss}(\omega)$ is the photon-loss bath spectral density which is assumed to be strict Ohmic (by assuming $\omega_{\rm m} \to \infty$). As a result, Eqs. S47 and S45 can be further expressed

as follows

$$P_j(\omega) = -\omega^2 \left[1 + \frac{2}{\pi}\int_0^\infty d\omega' \frac{J_{\mathrm{Q},j}(\omega')}{\omega'(\omega'^2 - \omega^2)}\right] = -\omega^2 - \frac{2\lambda_{\mathrm{Q},j}\omega}{\omega + i\gamma_{\mathrm{Q},j}}, \tag{S50a}$$

$$L(\omega) = -\omega^2 \left[1 + \frac{2}{\pi}\int_0^\infty d\omega' \frac{J_{\mathrm{loss}}(\omega')}{\omega'(\omega'^2 - \omega^2)}\right] = -\omega^2 - i\tau_{\mathrm{c}}^{-1}\omega. \tag{S50b}$$

To proceed, we further express Eq. S46 in a matrix form as

$$\boldsymbol{\mathcal{M}}\tilde{\boldsymbol{\mathcal{Q}}} = \boldsymbol{\mathcal{C}}\tilde{R}_0, \tag{S51}$$

where $\tilde{\boldsymbol{\mathcal{Q}}} = [\tilde{\mathcal{Q}}_1, \cdots, \tilde{\mathcal{Q}}_N]^{\mathrm{T}}$, $\boldsymbol{\mathcal{C}} = [\mathcal{C}_1, \cdots, \mathcal{C}_N]^{\mathrm{T}}$, and $\boldsymbol{\mathcal{M}}$ is an N-dimensional matrix defined as follows

$$\boldsymbol{\mathcal{M}} = \begin{bmatrix} \omega_1^2 + P_1(\omega) + \psi(\omega)\cdot\nu_1^2 & \psi(\omega)\cdot\nu_2\nu_1 & \cdots & \psi(\omega)\cdot\nu_N\nu_1 \\ \psi(\omega)\cdot\nu_1\nu_2 & \omega_2^2 + P_2(\omega) + \psi(\omega)\cdot\nu_1^2 & \cdots & \psi(\omega)\cdot\nu_N\nu_2 \\ \vdots & \vdots & \ddots & \vdots \\ \psi(\omega)\cdot\nu_1\nu_N & \psi(\omega)\cdot\nu_2\nu_N & \cdots & \omega_N^2 + P_N(\omega) + \psi(\omega)\cdot\nu_N^2 \end{bmatrix}, \tag{S52}$$

where $\psi(\omega) = \omega_{\mathrm{c}}^2 L(\omega)/(\omega_{\mathrm{c}}^2 + L(\omega))$. Using Eq. S51, one has

$$\sum_{j=1}^{N} \mathcal{C}_j\tilde{\mathcal{Q}}_j = \boldsymbol{\mathcal{C}}^{\mathrm{T}}\cdot\tilde{\boldsymbol{\mathcal{Q}}} = \boldsymbol{\mathcal{C}}^{\mathrm{T}}\boldsymbol{\mathcal{M}}^{-1}\boldsymbol{\mathcal{C}}\tilde{R}_0. \tag{S53}$$

Using Eq. S53, Eq. S42a is then expressed as

$$-\tilde{V}'_\omega(\tilde{R}_0) = \mathcal{K}(\omega)\tilde{R}_0, \qquad \mathcal{K}(\omega) \equiv \boldsymbol{\mathcal{C}}_\omega^T\boldsymbol{\mathcal{C}}_\omega - \boldsymbol{\mathcal{C}}^T\boldsymbol{\mathcal{M}}^{-1}\boldsymbol{\mathcal{C}} - \omega^2, \tag{S54}$$

where $\boldsymbol{\mathcal{C}}_\omega = [\mathcal{C}_1/\omega_1, \cdots, \mathcal{C}_N/\omega_N]^T$. Finally, the effective spectral density is given by[34,35]

$$J_{\mathrm{eff}}(\omega) \equiv \lim_{\epsilon\to 0^+} \mathrm{Im}\left[\mathcal{K}(\omega - i\epsilon)\right] = \mathrm{Im}\left[\boldsymbol{\mathcal{C}}^T\boldsymbol{\mathcal{M}}^{-1}\boldsymbol{\mathcal{C}}\right]; \quad \omega \in \mathbb{R}. \tag{S55}$$

Eq. S54 and Eq. S55 are the most general formulations of the effective spectral density, as presented in the method section. Unfortunately, it is not an easy task to obtain a simple analytic form for this expression. Next, we will present specific cases of the theory, as well as the numerical

demonstrations.

B. Uniform light-matter and spectator - reaction coordinate interactions

We consider the case for which (1) the light-matter coupling, (2) the spectator modes frequency, and (3) the coupling to the reaction coordinate are the same for all the spectator modes, and are fully aligned with the cavity field polarization direction. This means that $\nu_j \to \nu_{\rm c}$, $\omega_j \to \omega_{\rm Q}$ and $\mathcal{C}_j \to \mathcal{C}$. Under these limits, Eq. S46 becomes

$$[\omega_{\rm Q}^2 + P_{j'}(\omega)]\tilde{\mathcal{Q}}_{j'} + \frac{\omega_{\rm c}^2 \nu^2 L(\omega) \sum_j \tilde{\mathcal{Q}}_j}{\omega_{\rm c}^2 + L(\omega)} = \mathcal{C}\tilde{R}_0. \tag{S56}$$

Summing both sides of the above equation over the different j', we have

$$[\omega_Q^2 + P_{j'}(\omega)] \sum_{j'} \tilde{\mathcal{Q}}_{j'} + \frac{N\omega_{\rm c}^2 \nu^2 L(\omega) \sum_j \tilde{\mathcal{Q}}_j}{\omega_{\rm c}^2 + L(\omega)} = N\mathcal{C}\tilde{R}_0 \tag{S57}$$

which leads to

$$\sum_j \tilde{\mathcal{Q}}_j(\omega) = \frac{N\mathcal{C}}{\omega_{\rm Q}^2 + P_j(\omega) + \psi(\omega)} \tilde{R}_0, \tag{S58}$$

where

$$\psi(\omega) = \frac{2N\omega_{\rm c}\eta_{\rm c}^2 L(\omega)}{\omega_{\rm c}^2 + L(\omega)}. \tag{S59}$$

and P_j is defined in Eq. S50a. Further, under the current special limit, Eq. S42a can be written as

$$\mathcal{K}(\omega)\tilde{R}_0 = -\tilde{V}'_\omega(\tilde{R}_0), \tag{S60}$$

where

$$\mathcal{K}(\omega) = \frac{N\mathcal{C}^2}{\omega_{\rm Q}^2} \cdot \frac{P(\omega) + \psi(\omega)}{\omega_{\rm Q}^2 + P(\omega) + \psi(\omega)} - \omega^2, \tag{S61}$$

and $P(\omega)$ is expressed in Eq. S50a, $\psi(\omega) = \omega_{\rm c}^2 L(\omega)/(\omega_{\rm c}^2 + L(\omega))$, and $L(\omega)$ is expressed in Eq. S50b.

Finally, the effective spectral density is given by $J_{\text{eff}}(\omega) = \lim_{\epsilon\to 0^+} \text{Im}\left[\mathcal{K}(\omega - i\epsilon)\right]$, leading to

$$J_{\text{eff}}(\omega) = \frac{N\mathcal{C}^2}{2} \frac{\Gamma_{\text{Q}}(\omega)\omega}{\omega^2\Gamma_{\text{Q}}^2(\omega) + (\omega_{\text{Q}}^2 - \omega^2 + \tilde{\mathcal{R}}(\omega))^2}, \tag{S62}$$

where Γ_{Q} and $\tilde{\mathcal{R}}(\omega)$ are expressed as

$$\Gamma_{\text{Q}} = J_{\text{Q}}(\omega) + \frac{2N\omega_{\text{c}}^3\eta_{\text{c}}^2 J_{\text{loss}}(\omega)}{\left[\omega_{\text{c}}^2 - \omega^2 + R_{\text{c}}(\omega)\right]^2 + J_{\text{loss}}^2(\omega)}, \tag{S63a}$$

$$\tilde{\mathcal{R}}(\omega) = \mathcal{R}_{\text{Q}}(\omega) + \frac{2N\omega_{\text{c}}\eta_{\text{c}}^2 \left\{\left[\omega^2 - \mathcal{R}_{\text{c}}(\omega)\right]^2 - (\omega\omega_{\text{c}})^2 + \omega_{\text{c}}^2\mathcal{R}_{\text{c}}(\omega) + J_{\text{loss}}^2(\omega)\right\}}{\left[\omega_{\text{c}}^2 - \omega^2 + \mathcal{R}_{\text{c}}(\omega)\right]^2 + J_{\text{loss}}^2(\omega)}. \tag{S63b}$$

where $\mathcal{R}_{\text{c}}(\omega) = \frac{\omega}{\gamma_{\text{c}}} J_{\text{loss}}(\omega)$ and $\mathcal{R}_{\text{Q}}(\omega) = \frac{\omega}{\gamma_{\text{Q}}} J_{\text{Q}}(\omega)$. Under the Markovian limit, $\{\gamma_{\text{Q}}, \gamma_{\text{c}}\} \to \infty$, then $\{\mathcal{R}_{\text{Q}}(\omega), \mathcal{R}_{\text{c}}(\omega)\} \to 0$, and Eq. S63 is simplified as

$$\Gamma_{\text{Q}}(\omega) = \frac{2\lambda_{\text{Q}}}{\gamma_{\text{Q}}} + \frac{2N\omega_{\text{c}}^3\eta_{\text{c}}^2\tau_{\text{c}}^{-1}}{\left(\omega_{\text{c}}^2 - \omega^2\right)^2 + \omega^2\tau_{\text{c}}^{-2}}, \tag{S64a}$$

and $\tilde{\mathcal{R}}$

$$\tilde{\mathcal{R}}(\omega) = \frac{2N\omega_{\text{c}}\eta_{\text{c}}^2\omega^2}{\left(\omega_{\text{c}}^2 - \omega^2\right)^2 + \omega^2\tau_{\text{c}}^{-2}} \cdot (\omega^2 - \omega_{\text{c}}^2 + \tau_{\text{c}}^{-2}). \tag{S64b}$$

Eq. S62 (using Eq. S64 and Eq. S64b) is presented in Fig. S7 alongside the numerical solution of Eq. S55 with the restrictions considered in this section. The analytical and numerical results perfectly match in this case. Interestingly, as long as the light-matter interaction and the spectator - Rxn coupling is the same for each molecule, only the upper and lower polariton states appear in $\text{J}_{\text{eff}}(\omega)$.

C. Uniform $\mathcal{Q}_j - R_0$ couplings and disorders in dipole orientations

We consider a certain distribution of the angle φ_i, with identical (1) the light-matter coupling strength η_{c}, such that $\eta_i \to \eta_{\text{c}} \cdot \cos\varphi_i$, (2) identical spectator mode frequency the frequency $\omega_i \to \omega_{\text{Q}}$, and (3) identical coupling strength between $\mathcal{Q}_j$ and R_0, so $\mathcal{C}_i \to \mathcal{C}$. By summing over the different

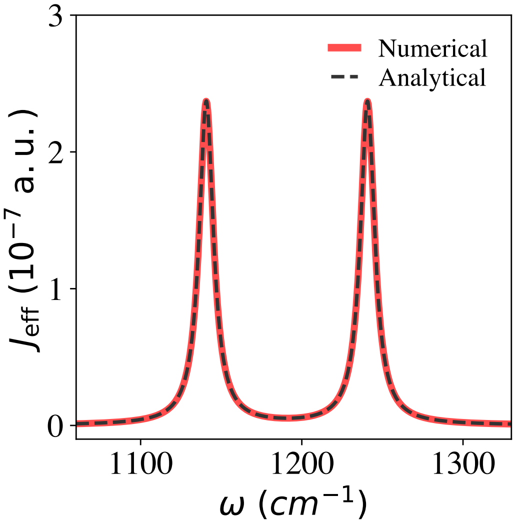

Figure S7: Numerical solution to Eq. S55 (solid line) and its analytical solution (Eq. S62, dashed line) when a uniform light-matter interaction ($\cos\varphi_j = 1$ for all j) and spectator mode-Rxn coupling ($\mathcal{C}_j = \mathcal{C}$) is considered. Here, we use $N = 1000$, $\Omega_{\mathrm{R}} = 100$ cm^{-1}, $\mathcal{C} = 0.15$ cm^{-1} and $\tau_{\mathrm{c}} = 500$ fs.

j', Equation S42b becomes

$$[\omega_{\mathrm{Q}}^2 + P(\omega)] \sum_{j'} \tilde{\mathcal{Q}}_{j'} + \frac{\omega_{\mathrm{c}}^2 L(\omega) \sum_j \nu_j \tilde{\mathcal{Q}}_j}{\omega_{\mathrm{c}}^2 + L(\omega)} \cdot \sum_j \nu_{j'} = N\mathcal{C}\tilde{R}_0(\omega). \tag{S65}$$

Taking $\nu_j = \sqrt{2/\omega_{\mathrm{c}}}\eta_{\mathrm{c}} \cdot \cos\varphi_j$, one can simplify the above equation as

$$[\omega_{\mathrm{Q}}^2 + P(\omega)] \sum_j \tilde{\mathcal{Q}}_j + \frac{2\omega_{\mathrm{c}}\eta_{\mathrm{c}}^2 L(\omega) \sum_{j'} \cos\varphi_{j'} \tilde{\mathcal{Q}}_{j'}}{\omega_{\mathrm{c}}^2 + L(\omega)} \cdot \sum_j \cos\varphi_j = N\mathcal{C}\tilde{R}_0(\omega). \tag{S66}$$

Defining $\chi = 1/N \sum_j \cos\varphi_j \equiv \langle\cos\varphi\rangle$ and approximating $\sum_{j'} \cos\varphi_{j'} \tilde{\mathcal{Q}}_{j'} \approx \chi \sum_{j'} \tilde{\mathcal{Q}}_{j'}$, one has

$$\left[\omega_{\mathrm{Q}}^2 + P(\omega) + \frac{2N\omega_{\mathrm{c}}\eta_{\mathrm{c}}^2\chi^2 L(\omega)}{\omega_{\mathrm{c}}^2 + L(\omega)}\right] \sum_j \tilde{\mathcal{Q}}_j = N\mathcal{C}\tilde{R}_0(\omega), \tag{S67}$$

which leads to

$$\sum_j \tilde{\mathcal{Q}}_j = \frac{N\mathcal{C}}{\omega_{\mathrm{Q}}^2 + P(\omega) + \psi(\omega)} \tilde{R}_0(\omega), \tag{S68}$$

with

$$\psi(\omega) = \frac{2N\omega_\mathrm{c}\eta_\mathrm{c}^2\chi^2 L(\omega)}{\omega_\mathrm{c}^2 + L(\omega)}. \tag{S69}$$

Finally, Eq. S42a is written as

$$\begin{aligned}
-\tilde{V}'_\omega(\tilde{R}_0) &= \left(N\frac{\mathcal{C}^2}{\omega_\mathrm{Q}^2} - \omega^2\right)\tilde{R}_0(\omega) - \mathcal{C}\sum_j \tilde{\mathcal{Q}}_j, \\
-\tilde{V}'_\omega(\tilde{R}_0) &= \left(\frac{N\mathcal{C}^2}{\omega_\mathrm{Q}^2}\cdot\frac{P(\omega)+\psi(\omega)}{\omega_\mathrm{Q}^2 + P(\omega) + \psi(\omega)} - \omega^2\right)\tilde{R}_0.
\end{aligned} \tag{S70}$$

Following the steps outlined in the previous section, the spectral density under these conditions is

$$J_\mathrm{eff}(\omega) = \frac{N\mathcal{C}^2}{2}\frac{\Gamma_\mathrm{Q}(\omega)\omega}{\omega^2\Gamma_\mathrm{Q}^2(\omega) + (\omega_\mathrm{Q}^2 - \omega^2 + \tilde{\mathcal{R}}(\omega))^2}, \tag{S71}$$

with

$$\Gamma_\mathrm{Q}(\omega) = \frac{2\lambda_\mathrm{Q}}{\gamma_\mathrm{Q}} + \frac{2N\omega_\mathrm{c}^3\eta_\mathrm{c}^2\chi^2\tau_\mathrm{c}^{-1}}{\left(\omega_\mathrm{c}^2 - \omega^2\right)^2 + \omega^2\tau_\mathrm{c}^{-2}}, \tag{S72a}$$

and $\tilde{\mathcal{R}}$

$$\tilde{\mathcal{R}}(\omega) = \frac{2N\omega_\mathrm{c}\eta_\mathrm{c}^2\chi^2\omega^2}{\left(\omega_\mathrm{c}^2 - \omega^2\right)^2 + \omega^2\tau_\mathrm{c}^{-2}}\cdot(\omega^2 - \omega_\mathrm{c}^2 + \tau_\mathrm{c}^{-2}). \tag{S72b}$$

Particularly, in the limit of large N, it is expected that $\chi \to 0$ in an isotropic distribution of dipoles and the cavity is completely decoupled from the matter part. Fig. S8 shows the numerical solution to Eq. S55 using the conditions considered in this section. Increasing the dipole disorder decreases the cavity effects as the dark states' spectral density starts to emerge. In the isotropic case, the polaritonic states disappear meaning that the matter DOF is completely decoupled from the cavity as discussed before.

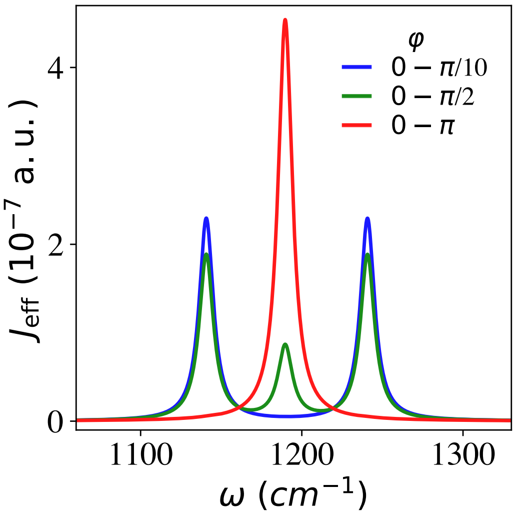

Figure S8: Numerical solution of Eq. S55 for a uniform spectator mode - Rxn coupling and varying the angle between the molecular dipole and the direction of the cavity field (φ_j); the angles are sample from a uniform distribution given a certain range. Here case $N = 1000$, $\Omega_{\mathrm{R}} = 100$ cm^{-1} and $\tau_{\mathrm{c}} = 500$ fs, the reorganization energy (λ_{eff}) is constant.

D. Uniform light-matter interaction and $\mathcal{C}_j$ disorder

Here, we consider a uniform light-matter interaction where $\cos\varphi_j = 1$, and the spectator modes have varying coupling $\mathcal{C}_j$ with the reaction coordinate. Then, Eq. S42b is written as

$$[\omega_{\mathrm{Q}}^2 + P_{j'}(\omega)]\tilde{\mathcal{Q}}_{j'} + \frac{\omega_{\mathrm{c}}^2 \nu^2 L(\omega) \sum_j \tilde{\mathcal{Q}}_j}{\omega_{\mathrm{c}}^2 + L(\omega)} = \mathcal{C}_{j'} \tilde{R}_0. \tag{S73}$$

Multiplying on both sides by $\mathcal{C}_{j'}$ and summing over all j'

$$[\omega_{\mathrm{Q}}^2 + P_{j'}(\omega)] \sum_{j'} \mathcal{C}_{j'} \tilde{\mathcal{Q}}_{j'} + \frac{\omega_{\mathrm{c}}^2 \nu^2 L(\omega)}{\omega_{\mathrm{c}}^2 + L(\omega)} \sum_j \tilde{\mathcal{Q}}_j \sum_{j'} \mathcal{C}_{j'} = \sum_{j'} \mathcal{C}_{j'}^2 \tilde{R}_0. \tag{S74}$$

As a **crude approximation**, we consider $\sum_j \tilde{\mathcal{Q}}_j \sum_{j'} \mathcal{C}_{j'} \approx N \sum_j \mathcal{C}_{j'} \tilde{\mathcal{Q}}_{j'}$, which leads to

$$[\omega_{\mathrm{Q}}^2 + P_{j'}(\omega)] \sum_{j'} \mathcal{C}_{j'} \tilde{\mathcal{Q}}_{j'} + \frac{2N\omega_{\mathrm{c}} \eta_{\mathrm{c}}^2 L(\omega)}{\omega_{\mathrm{c}}^2 + L(\omega)} \sum_j \mathcal{C}_{j'} \tilde{\mathcal{Q}}_{j'} = \sum_{j'} \mathcal{C}_{j'}^2 \tilde{R}_0, \tag{S75}$$

$$\sum_{j'} \mathcal{C}_{j'} \tilde{\mathcal{Q}}_{j'} = \frac{\sum_{j'} \mathcal{C}_{j'}^2 \tilde{R}_0}{\omega_{\mathrm{Q}}^2 + P_{j'}(\omega) + \psi(\omega)}, \tag{S76}$$

where we have used $\nu = \sqrt{2/\omega_c}\eta_c$, $\psi(\omega) = \frac{2N\omega_c\eta_c^2 L(\omega)}{\omega_c^2+L(\omega)}$ and $P_j(\omega)$ has been previously defined. Finally, Eq. S42a becomes

$$-\tilde{V}'_\omega(\tilde{R}_0) = \left(\sum_j \frac{\mathcal{C}_j^2}{\omega_Q^2} - \omega^2\right)\tilde{R}_0(\omega) - \frac{\sum_j \mathcal{C}_j^2 \tilde{R}_0}{\omega_Q^2 + P_j(\omega) + \psi(\omega)}, \tag{S77}$$

$$-\tilde{V}'_\omega(\tilde{R}_0) = \left(\frac{P_j(\omega)+\psi(\omega)}{\omega_Q^2 + P_j(\omega) + \psi(\omega)} \sum_j \frac{\mathcal{C}_j^2}{\omega_Q^2} - \omega^2\right)\tilde{R}_0, \tag{S78}$$

and we retrieve the effective spectral density

$$J_{\text{eff}}(\omega) = \frac{\Lambda\omega_Q^2 \cdot \omega\Gamma_Q(\omega)}{\left[\omega_Q^2 - \omega^2 + \tilde{\mathcal{R}}(\omega)\right]^2 + [\omega\Gamma_Q(\omega)]^2}, \tag{S79}$$

where $\Lambda = \sum_j \mathcal{C}_j^2/(2\omega_Q^2)$.

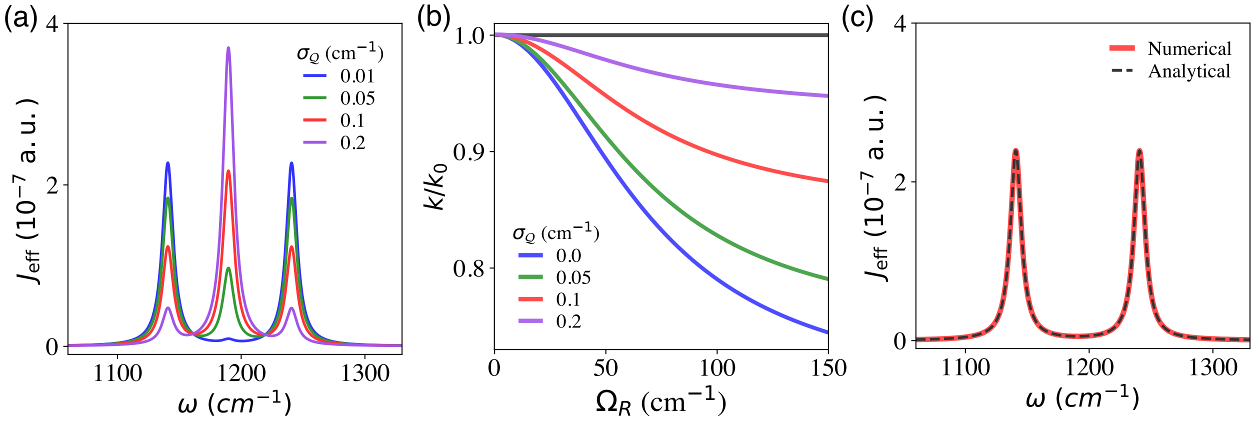

Figure S9: (**a**) Numerical solution to Eq S55 for a uniform light-matter interaction and disordered spectator mode - Rxn coupling; the $\mathcal{C}_i$ couplings are sample from a normal distribution with standard deviation $\sigma_\mathcal{Q}$. (**b**) k/k_0 as a function of Ω_R using the J_{eff} presented in (**a**). (**c**) Numerical (solid line) and analytical (Eq. S90, dashed line) solutions taking the approximation used in Eq. S75 for a $\varepsilon = 0.5$. Here, we use $N = 1000$, $\Omega_R = 100$ cm^{-1} and $\tau_c = 500$ fs, the reorganization energy (Λ) is constant.

Fig. S9**a** presents the numerical solution of Eq. S55 for the conditions assumed in this section; the spectator mode - Rxn couplings $\mathcal{C}_j$ are sampled from a normal distribution with standard deviation $\sigma_\mathcal{Q}$; the results show that increasing the disorder of the couplings reduces the cavity effects and the dark states start to emerge in the effective spectral density, the larger the disorder of the system, the larger the role of the dark states to the point where these take over the polaritonic states; thus,

it is expected that the cavity effects would be negligible as shown in Fig. S9(**b**). Fig. S9(**c**) shows the comparison between the numerical and analytic (Eq. S90) solutions using the approximation in Eq. S75, both solutions match perfectly, however, any effects that the disorder has on the $J_{\text{eff}}(\omega)$ shape are washed out.

E. Static Disorder in spectator modes' frequency ω_{Q}

We further consider inhomogeneous broadening to the spectator modes frequency ω_{Q} by modeling it as a Gaussian static disorder, as considered in the previous work,[36] while keeping the dipoles fully aligned with the cavity field (keep $\chi = 1$) and a uniform $\mathcal{C}_j$ coupling for $R_0 - \mathcal{Q}_j$.

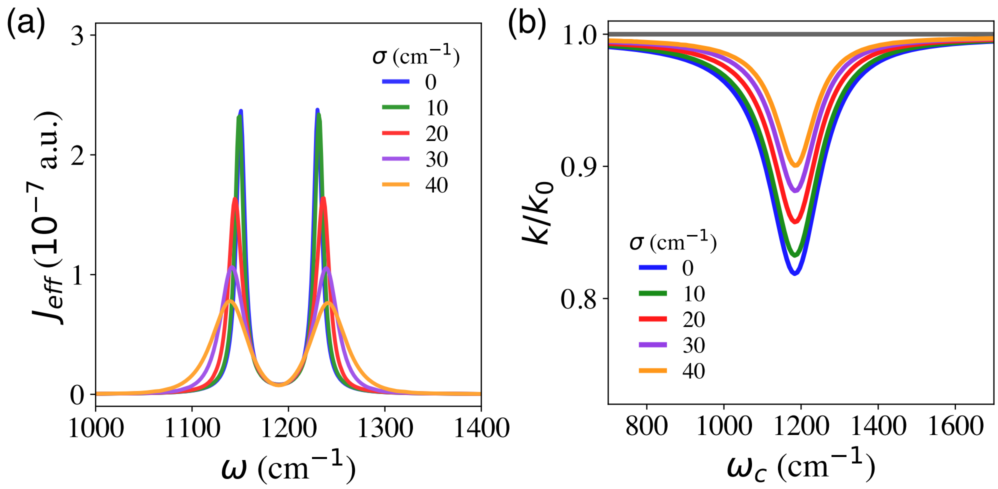

Figure S10: (**a**) Effective spectral density $J_{\text{eff}}(\omega)$ obtained from the numerical solution of Eq. S55 for a system with the static disorder of ω_{Q} (with gaussian disorder and width σ) and a uniform light-matter coupling strength. Here, we use $N = 1000$, and $\tau_{\text{c}} = 500$ fs. The light-matter coupling strength is $g_{\text{c}} = 1.26$ cm^{-1} per molecule, such that when $\sigma = 0$ for $N = 1000$, the collective Rabi splitting is $\Omega_{\text{R}} = 80$ cm^{-1}. (**b**) k/k_0 as a function of the cavity frequency ω_{c} using the $J_{\text{eff}}(\omega)$ presented in (**a**).

Fig. S10a presents the effective spectral density for a system with static disorder in frequency ω_j (c.f. Eq. S39b), which satisfies the Gaussian normal distribution $P(\omega_j) \sim e^{-(\omega_j - \omega_{\text{Q}})^2/2\sigma^2}$, where the value of the standard deviation is σ. The light-matter coupling strength (per molecule) is $g_{\text{c}} = \eta_{\text{c}}\omega_{\text{c}}\mu_{\text{Q}} = 1.26$ cm^{-1}. When $\sigma = 0$, the corresponding η_{c} correspond to a collective $\Omega_{\text{R}} = 80$ cm^{-1} for $N = 1000$ molecules. This type of static disorder on ω_{Q} broadens the shape of the spectral density profile $J_{\text{eff}}(\omega)$, evaluated using Eq. S55. As can be seen from the figure, when increasing the width of the static disorder σ while keeping the effective reorganization energy $\lambda_{\text{eff}} = \int d\omega \frac{J_{\text{eff}}(\omega)}{\omega}$

on R_0 as a constant, the height of the spectral density decreases, diminishing its influence on the rate constant (see Eq. 8 in the main text). Notably, we do not observe a significant emergence of dark states with σ, in contrast with the results provided in Fig. S8 and Fig. S9a. In addition, it seems that the effective Rabi splitting between two polariton peaks is increased when increasing σ. This is consistent with the recent theoretical work,[37,38] which suggests the molecular disorder perturbatively couples the polaritons (when $\sigma^2 < \sqrt{N} g_c$) to the manifold of dark states, inducing level repulsion between the polaritons.

Fig. S10b presents the corresponding rate constant with various static disorders on ω_Q. Here, we use the same FGR expression to evaluate k_{VSC}

$$k_{\mathrm{VSC}} = \int_0^{\infty} d\omega \; 2|\Delta_x|^2 \cdot \mathcal{A}_0(\omega - \omega_0) \cdot J_{\mathrm{eff}}(\omega) \cdot n(\omega), \tag{S80}$$

where $J_{\mathrm{eff}}(\omega)$ is now evaluated numerically with Eq. S55 (as shown in Fig. S10a), $\mathcal{A}_0(\omega - \omega_0)$ is the broadening function in Eq. S5, and $n(\omega)$ is the Bose-Einstein distribution function

$$n(\omega) = 1/(e^{\beta\hbar\omega} - 1) \approx e^{-\beta\hbar\omega}. \tag{S81}$$

We report the rate constant changes using k/k_0, and evaluate $k_0 = k_{\mathrm{D}} + \alpha \cdot k_{\mathrm{VSC}}(\eta_c = 0)$ with $\alpha = 0.7$ (which is the same universal scaling parameter we used in this work), and k_{D} is the bare double well rate constant obtained from the HEOM (see Method section in the main text). Here, $k_{\mathrm{VSC}}(\eta_c = 0)$ is evaluated using Eq. S80 by setting $\eta_c = 0$ in Eq. S55. The results indicate that the cavity can still modify reaction dynamics despite different levels of inhomogeneous broadening. Therefore, the variation in the cavity effect is primarily due to the reduced influence of the spectator modes on the reaction. This suggests that disorders in dipole orientation and reaction-spectator mode coupling are more relevant to our model.

F. Generalized case in C and D

The approximations presented in Sections C and D can be combined to provide a more general expression, which in principle contains disorders in light-matter coupling and $Q_j - R_0$ couplings. Starting from Eq. S46 and taking all the spectator modes to have the same frequency ($\omega_j = \omega_Q$),

we have

$$[\omega_{\rm Q}^2 + P_{j'}(\omega)]\tilde{\mathcal{Q}}_{j'} + \frac{\omega_{\rm c}^2 L(\omega)\sum_j \nu_j \tilde{\mathcal{Q}}_j}{\omega_{\rm c}^2 + L(\omega)}\nu_{j'} = \mathcal{C}_{j'}\tilde{R}_0, \tag{S82}$$

Multiplying bothside of the above equation by $\mathcal{C}_{j'}$ and summing over j', we have

$$[\omega_{\rm Q}^2 + P_{j'}(\omega)]\sum_{j'}\mathcal{C}_{j'}\tilde{\mathcal{Q}}_{j'} + \frac{\omega_{\rm c}^2 L(\omega)\sum_j \nu_j \tilde{\mathcal{Q}}_j}{\omega_{\rm c}^2 + L(\omega)}\sum_{j'}\mathcal{C}_{j'}\nu_{j'} = \sum_{j'}\mathcal{C}_{j'}^2\tilde{R}_0, \tag{S83}$$

Taking $\nu_j = \sqrt{2/\omega_{\rm c}}\eta_{\rm c} \cdot \cos\varphi_j$

$$[\omega_{\rm Q}^2 + P_{j'}(\omega)]\sum_{j'}\mathcal{C}_{j'}\tilde{\mathcal{Q}}_{j'} + \frac{2\omega_{\rm c}\eta_c^2 L(\omega)\sum_j \cos\varphi_j \tilde{\mathcal{Q}}_j}{\omega_{\rm c}^2 + L(\omega)}\sum_{j'}\mathcal{C}_{j'}\cos\varphi_{j'} = \sum_{j'}\mathcal{C}_{j'}^2\tilde{R}_0, \tag{S84}$$

Assuming a meanfield like approximation, $\cos\varphi_j \approx \langle\cos\varphi\rangle = \chi$, the above equation becomes

$$[\omega_{\rm Q}^2 + P_{j'}(\omega)]\sum_{j'}\mathcal{C}_{j'}\tilde{\mathcal{Q}}_{j'} + \frac{2\omega_{\rm c}\eta_c^2\chi^2 L(\omega)}{\omega_{\rm c}^2 + L(\omega)}\sum_{j}\tilde{\mathcal{Q}}_j\sum_{j'}\mathcal{C}_{j'} = \sum_{j'}\mathcal{C}_{j'}^2\tilde{R}_0, \tag{S85}$$

Now, if we consider another approximation, $\sum_j \tilde{\mathcal{Q}}_j \sum_{j'}\mathcal{C}_{j'} \approx N\sum_j \mathcal{C}_{j'}\tilde{\mathcal{Q}}_{j'}$, the previous equation becomes

$$[\omega_{\rm Q}^2 + P_{j'}(\omega)]\sum_{j'}\mathcal{C}_{j'}\tilde{\mathcal{Q}}_{j'} + \frac{2N\omega_{\rm c}\eta_c^2\chi^2 L(\omega)}{\omega_{\rm c}^2 + L(\omega)}\sum_{j'}\mathcal{C}_{j'}\tilde{\mathcal{Q}}_{j'} = \sum_{j'}\mathcal{C}_{j'}^2\tilde{R}_0, \tag{S86}$$

$$\sum_{j'}\mathcal{C}_{j'}\tilde{\mathcal{Q}}_{j'} = \frac{\sum_{j'}\mathcal{C}_{j'}^2\tilde{R}_0}{\omega_{\rm Q}^2 + P_{j'}(\omega) + \psi(\omega)}, \tag{S87}$$

where we have used $\psi(\omega) = \frac{2N\omega_{\rm c}\eta_c^2\chi^2 L(\omega)}{\omega_{\rm c}^2 + L(\omega)}$. Finally, Eq. S42a becomes

$$-\tilde{V}'_\omega(\tilde{R}_0) = \left(\sum_j \frac{\mathcal{C}_j^2}{\omega_{\rm Q}^2} - \omega^2\right)\tilde{R}_0(\omega) - \frac{\sum_j \mathcal{C}_j^2\tilde{R}_0}{\omega_{\rm Q}^2 + P_j(\omega) + \psi(\omega)}, \tag{S88}$$

$$-\tilde{V}'_\omega(\tilde{R}_0) = \left(\frac{P_j(\omega) + \psi(\omega)}{\omega_{\rm Q}^2 + P_j(\omega) + \psi(\omega)}\sum_j \frac{\mathcal{C}_j^2}{\omega_{\rm Q}^2} - \omega^2\right)\tilde{R}_0, \tag{S89}$$

and we retrieve the effective spectral density

$$J_{\text{eff}}(\omega) = \frac{\Lambda\omega_{\text{Q}}^2 \cdot \omega\Gamma_{\text{Q}}(\omega)}{\left[\omega_{\text{Q}}^2 - \omega^2 + \tilde{\mathcal{R}}(\omega)\right]^2 + [\omega\Gamma_{\text{Q}}(\omega)]^2}, \tag{S90}$$

where $\Lambda = \sum_j \mathcal{C}_{\text{j}}^2/(2\omega_{\text{Q}}^2)$ (see Eq. 3 of the main text and c.f. Eq. S40), Γ_{Q} is expressed

$$\Gamma_{\text{Q}}(\omega) = \frac{2\lambda_{\text{Q}}}{\gamma_{\text{Q}}} + \frac{2N\omega_{\text{c}}^3\eta_{\text{c}}^2\chi^2\tau_{\text{c}}^{-1}}{(\omega_{\text{c}}^2 - \omega^2)^2 + \omega^2\tau_{\text{c}}^{-2}}, \tag{S91a}$$

and $\tilde{\mathcal{R}}$ is expressed as

$$\tilde{\mathcal{R}}(\omega) = \frac{2N\omega_{\text{c}}\eta_{\text{c}}^2\chi^2\omega^2}{(\omega_{\text{c}}^2 - \omega^2)^2 + \omega^2\tau_{\text{c}}^{-2}} \cdot (\omega^2 - \omega_{\text{c}}^2 + \tau_{\text{c}}^{-2}). \tag{S91b}$$

which are Eqs. 9 presented in the main text.

Supporting Information 6: $J_{\text{eff}}(\omega)$ Simplification and Scaling Analysis

A. Lossless cavity

For the parameters considered in this work, we found that the rate modification is not very sensitive to the change in cavity lifetime once the strong coupling condition has been reached. In order to simplify Eq. S71

$$J_{\text{eff}}(\omega) = \frac{N\mathcal{C}^2}{2} \frac{\omega\Gamma_{\text{Q}}(\omega)}{[\omega_{\text{Q}}^2 - \omega^2 + \tilde{\mathcal{R}}(\omega)]^2 + \omega^2\Gamma_{\text{Q}}(\omega)^2}, \tag{S92}$$

where

$$\omega\Gamma_{\text{Q}}(\omega) = \frac{2\omega\lambda_{\text{Q}}}{\gamma_{\text{Q}}} + \frac{2N\omega_{\text{c}}^3\eta_{\text{c}}^2\chi^2\tau_{\text{c}}^{-1}\omega}{(\omega_{\text{c}}^2 - \omega^2)^2 + (\omega\tau_{\text{c}}^{-1})^2}, \tag{S93}$$

$$\tilde{\mathcal{R}}(\omega) = \frac{2N\omega_{\text{c}}\eta_{\text{c}}^2\chi^2\omega^2}{(\omega_{\text{c}}^2 - \omega^2)^2 + (\omega\tau_{\text{c}}^{-1})^2}(\omega^2 - \omega_{\text{c}}^2 + \tau_{\text{c}}^{-2}). \tag{S94}$$

we take the limit at which $\tau_{\text{c}} \to \infty$

$$\omega\Gamma_{\text{Q}}(\omega) = \frac{2\omega\lambda_{\text{Q}}}{\gamma_{\text{Q}}} + \pi N\omega_{\text{c}}^2\eta_{\text{c}}^2\chi^2 \cdot \delta(\omega - \omega_{\text{c}}), \tag{S95}$$

$$\tilde{\mathcal{R}} = \frac{2N\omega_{\text{c}}\eta_{\text{c}}^2\chi^2\omega^2}{(\omega_{\text{c}}^2 - \omega^2)}. \tag{S96}$$

Here we have used the fact that $\lim_{\epsilon\to 0} \frac{\epsilon}{\pi(x^2+\epsilon^2)} = \delta(x)$. Further, as $\delta(a-x)\cdot(a-x)^n = 0$,

$$\omega\Gamma_{\text{Q}}(\omega)\cdot(\omega_{\text{c}}^2 - \omega^2) = \frac{2\omega\lambda_{\text{Q}}}{\gamma_{\text{Q}}}\cdot(\omega_{\text{c}}^2 - \omega^2). \tag{S97}$$

Finally, taking the resonance condition $\omega_{\text{Q}} = \omega_{\text{c}}$,

$$J_{\text{eff}}(\omega) = \frac{N\mathcal{C}^2}{2} \frac{\omega\Gamma_{\text{Q}}(\omega)\cdot(\omega_{\text{Q}}^2 - \omega^2)^2}{[(\omega_{\text{Q}}^2 - \omega^2)^2 - \chi^2\Omega_{\text{R}}^2\omega^2]^2 + [\omega\Gamma_{\text{Q}}(\omega)\cdot(\omega_{\text{Q}}^2 - \omega^2)]^2}, \tag{S98}$$

where $\Omega_{\text{R}} = \sqrt{2N\omega_{\text{c}}}\eta_{\text{c}}$.

B. Scaling analysis

From the previous equation, there is a $1/\Omega_R^2$ dependence of the effective spectral density which will be reflected in the rate constant. To get a sense of the rate constant scaling, we evaluated Eq. S71 at $\omega = \omega_0$.

$$J_{\text{eff}}(\omega_0) = \frac{N\mathcal{C}^2}{2} \frac{\Gamma_Q(\omega_0)\omega_0}{[\omega_Q^2 - \omega_0^2 + \tilde{\mathcal{R}}(\omega_0)]^2 + (\omega_0\Gamma_Q(\omega_0))^2}, \tag{S99}$$

where

$$\omega\Gamma_Q(\omega_0) = \frac{2\lambda_Q\omega_0}{\gamma_Q} + \frac{2N\omega_c^3\eta_c^2\chi^2\tau_c^{-1}\omega_0}{(\omega_c^2 - \omega_0^2)^2 + (\omega_0\tau_c^{-1})^2}, \tag{S100}$$

$$\tilde{\mathcal{R}}(\omega_0) = \frac{2N\omega_c\eta_c^2\chi^2\omega_0^2}{(\omega_c^2 - \omega_0^2)^2 + (\omega_0\tau_c^{-1})^2}(\omega_0^2 - \omega_c^2 + \tau_c^{-2}). \tag{S101}$$

At resonance ($\omega_c = \omega_Q$) and taking $\Omega_R = \sqrt{2N\omega_c}\eta_c$,

$$\omega_0\Gamma_Q(\omega_0) = \frac{2\lambda_Q\omega_0}{\gamma_Q} + \frac{\Omega_R^2\chi^2\omega_0}{\tau_c^{-1}}, \qquad \tilde{\mathcal{R}}(\omega_0) = \frac{\Omega_R^2\chi^2\omega_0^2}{(\omega_0\tau_c^{-1})^2}(\tau_c^{-2}) = \Omega_R^2\chi^2. \tag{S102}$$

And the effective spectral density becomes

$$J_{\text{eff}}(\omega_0) = \frac{N\mathcal{C}^2}{2} \frac{\Gamma_Q(\omega_0)\omega_0}{[\tilde{\mathcal{R}}(\omega_0)]^2 + [\omega_0\Gamma_Q(\omega_0)]^2} = \frac{N\mathcal{C}^2}{2} \frac{\Gamma_Q(\omega_0)\omega_0}{\Omega_R^4\chi^4 + [\omega_0\Gamma_Q(\omega_0)]^2}. \tag{S103}$$

Taking the limit $\frac{2\lambda_Q\omega_0}{\gamma_Q} \to 0$, then $\omega_0\Gamma_Q(\omega) \to \frac{\Omega_R^2\chi^2\omega_0}{\tau_c^{-1}}$,

$$J_{\text{eff}}(\omega_0) = \frac{N\mathcal{C}^2}{2\tau_c^{-1}} \frac{\Omega_R^2\chi^2\omega_0}{\Omega_R^4\chi^4 + \left[\frac{\Omega_R^2\chi^2\omega_0}{\tau_c^{-1}}\right]^2} = \frac{N\mathcal{C}^2}{2\Omega_R^2\chi^2} \cdot \frac{\omega_0\tau_c^{-1}}{\tau_c^{-2} + \omega_0^2}. \tag{S104}$$

The rate is given by the FGR constant,

$$k_{\text{VSC}} = 2|\Delta_x|^2 \cdot J_{\text{eff}}(\omega_0) \cdot n(\omega_0) = \frac{|\Delta_x|^2 n(\omega_0) \cdot N\mathcal{C}^2\omega_0\tau_c^{-1}}{\chi^2(\tau_c^{-2} + \omega_0^2)} \cdot \frac{1}{\Omega_R^2}. \tag{S105}$$

Therefore $k_{\text{VSC}} \propto \Omega_R^{-2}$. The convolution between J_{eff} and $\mathcal{A}_0$ (see Eqs. 11 of the main text) will give the basic scaling between k_{VSC} and Ω_R.

We fit k/k_0 using the following function

$$f(\Omega_R) = \frac{k_{\mathrm{D}}}{k_0} + \frac{a}{1 + b \cdot \Omega_{\mathrm{R}}^2}, \tag{S106}$$

where a and b are fitting parameters. Fig. S11 presents these fitting for (a) FGR prediction in Fig. 3a of the main text, (b) numerical simulation from HEOM in Fig. 3a of the main text, as well as experimental data taken from Ref. 39.

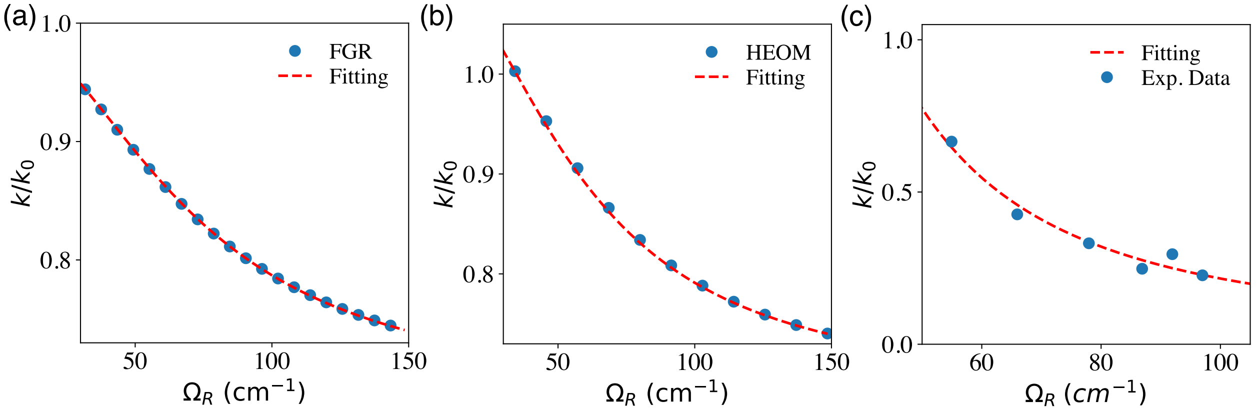

Figure S11: Fitting of Eq. S106 to (**a**) FGR and (**b**) HEOM results, under the resonance condition $\omega_{\mathrm{c}} = \omega_{\mathrm{Q}}$. The same fitting is done for (**c**) Ebbesen *et al.* experimental data.[39]

Supporting Information 7: Normal Mode Analysis

Here, we perform a normal mode transformation over the equation of motion. We closely follow the procedure outlined in Ref. 33. We start by considering the Hamiltonian

$$\hat{H} = \hat{H}_{\mathrm{M}} + \hat{H}_{\mathcal{Q}} + \hat{H}_{\mathrm{LM}}, \tag{S107}$$

with each term expressed as

$$\hat{H}_{\mathrm{M}} = \frac{\hat{P}_{\mathrm{M}}^2}{2} + \frac{\omega_0^2}{2}\hat{R}_0^2, \tag{S108a}$$

$$\hat{H}_{\mathcal{Q}} = \sum_{j=1}^{N}\left[\frac{\hat{\Pi}_j^2}{2} + \frac{\omega_j^2}{2}\left(\hat{\mathcal{Q}}_j - \frac{\mathcal{C}_j}{\omega_j^2}\cdot\hat{R}_0\right)^2\right], \tag{S108b}$$

$$\hat{H}_{\mathrm{LM}} = \frac{\hat{P}_{\mathrm{c}}^2}{2} + \frac{\omega_{\mathrm{c}}^2}{2}\left[\hat{q}_{\mathrm{c}} + \sum_{j=1}^{N}\nu_j\cdot\hat{\mathcal{Q}}_j\right]^2, \tag{S108c}$$

where we have approximated the left-side of the double-well potential as a harmonic oscillator of frequency ω_0. We consider these equations as classical, the equations of motion for this Hamiltonian are given by

$$-\ddot{R}_0 = \omega_0^2 R_0 - \sum_j \mathcal{C}_j\left(\mathcal{Q}_j - \frac{\mathcal{C}_j}{\omega_j^2}R_0\right), \tag{S109a}$$

$$-\ddot{\mathcal{Q}}_{j'} = \omega_{j'}^2\left(\mathcal{Q}_{j'} - \frac{\mathcal{C}_{j'}}{\omega_{j'}^2}R_0\right) + \omega_{\mathrm{c}}^2\nu_{j'}\left(q_{\mathrm{c}} + \sum_j \nu_j\mathcal{Q}_j\right), \tag{S109b}$$

$$-\ddot{q}_{\mathrm{c}} = \omega_{\mathrm{c}}^2\left(q_{\mathrm{c}} + \sum_j \nu_j\mathcal{Q}_j\right), \tag{S109c}$$

which can be expressed in matrix form as

$$-\begin{bmatrix} \ddot{R}_0 \\ \ddot{\mathcal{Q}}_1 \\ \ddot{\mathcal{Q}}_2 \\ \vdots \\ \ddot{\mathcal{Q}}_N \\ \ddot{q}_\mathrm{c} \end{bmatrix} = \begin{bmatrix} \omega_0^2 + \sum_j \frac{\mathcal{C}_j^2}{\omega_j^2} & -\mathcal{C}_1 & -\mathcal{C}_2 & \cdots & -\mathcal{C}_N & 0 \\ -\mathcal{C}_1 & \omega_1^2 + \omega_\mathrm{c}^2 \nu_1^2 & \omega_\mathrm{c}^2 \nu_1 \nu_2 & \cdots & \omega_\mathrm{c}^2 \nu_1 \nu_N & \omega_\mathrm{c}^2 \nu_1 \\ -\mathcal{C}_2 & \omega_\mathrm{c}^2 \nu_2 \nu_1 & \omega_2^2 + \omega_\mathrm{c}^2 \nu_2^2 & \cdots & \omega_\mathrm{c}^2 \nu_2 \nu_N & \omega_\mathrm{c}^2 \nu_2 \\ \vdots & \vdots & \vdots & \ddots & \vdots & \vdots \\ -\mathcal{C}_N & \omega_\mathrm{c}^2 \nu_N \nu_1 & \omega_\mathrm{c}^2 \nu_N \nu_2 & \cdots & \omega_N^2 + \omega_\mathrm{c}^2 \nu_N^2 & \omega_\mathrm{c}^2 \nu_N \\ 0 & \omega_\mathrm{c}^2 \nu_1 & \omega_\mathrm{c}^2 \nu_2 & \cdots & \omega_\mathrm{c}^2 \nu_N & \omega_\mathrm{c}^2 \end{bmatrix} \begin{bmatrix} R_0 \\ \mathcal{Q}_1 \\ \mathcal{Q}_2 \\ \vdots \\ \mathcal{Q}_N \\ q_\mathrm{c} \end{bmatrix}, \tag{S110}$$

or simply as

$$-\ddot{\boldsymbol{\mathcal{R}}} = \boldsymbol{\mathcal{H}} \boldsymbol{\mathcal{R}}. \tag{S111}$$

Here, $\boldsymbol{\mathcal{H}}$ is the Hessian matrix associated with the system. Taking the matrix

$$\mathbf{U_R} = \begin{bmatrix} 1 & 0 & 0 & \cdots & 0 & 0 \\ 0 & \mathcal{M}_1^1 & \mathcal{M}_2^1 & \cdots & \mathcal{M}_N^1 & 0 \\ 0 & \mathcal{M}_1^2 & \mathcal{M}_2^2 & \cdots & \mathcal{M}_N^2 & 0 \\ \vdots & \vdots & \vdots & \ddots & \vdots & \vdots \\ 0 & \mathcal{M}_1^N & \mathcal{M}_2^N & \cdots & \mathcal{M}_N^N & 0 \\ 0 & 0 & 0 & \cdots & 0 & 1 \end{bmatrix}. \tag{S112}$$

A unitary transformation is performed on Eq. S110 as

$$-\boldsymbol{U}_\mathbf{R} \ddot{\boldsymbol{\mathcal{R}}} = \boldsymbol{U}_\mathbf{R} \boldsymbol{\mathcal{H}} \boldsymbol{U}_\mathbf{R}^\dagger \cdot \boldsymbol{U}_\mathbf{R} \boldsymbol{\mathcal{R}}, \tag{S113}$$

which takes the left-hand side of the equation and the position vector to

$$\boldsymbol{U}_{\mathbf{R}}\ddot{\boldsymbol{\mathcal{R}}} = \begin{bmatrix} \ddot{R}_0 \\ \sum_i \mathcal{M}_i^1 \ddot{\mathcal{Q}}_i \\ \sum_i \mathcal{M}_i^2 \ddot{\mathcal{Q}}_i \\ \vdots \\ \sum_i \mathcal{M}_i^N \ddot{\mathcal{Q}}_i \\ \ddot{q}_\mathrm{c} \end{bmatrix}, \qquad \boldsymbol{U}_{\mathbf{R}}\boldsymbol{\mathcal{R}} = \begin{bmatrix} R_0 \\ \sum_i \mathcal{M}_i^1 \mathcal{Q}_i \\ \sum_i \mathcal{M}_i^2 \mathcal{Q}_i \\ \vdots \\ \sum_i \mathcal{M}_i^N \mathcal{Q}_i \\ q_\mathrm{c} \end{bmatrix}. \tag{S114}$$

Further, the Hessian matrix transformation $\boldsymbol{U}_{\mathbf{R}}\boldsymbol{\mathcal{H}}\boldsymbol{U}_{\mathbf{R}}^{\dagger}$ in the spectator subspace. It has diagonal elements

$$\Omega_i^2 = \sum_k^N \left(\mathcal{M}_k^i \omega_k\right)^2 + \omega_\mathrm{c}^2 \left(\sum_k \mathcal{M}_k^i \nu_k\right)^2 = \omega_\mathrm{Q}^2 \sum_k^N \left(\mathcal{M}_k^i\right)^2 + \omega_\mathrm{c}^2 \nu^2 \left(\sum_k \mathcal{M}_k^i\right)^2, \tag{S115}$$

and off-diagonal elements

$$d_{ij} = \sum_k^N \mathcal{M}_k^i \mathcal{M}_k^j \omega_k^2 + \omega_\mathrm{c}^2 \left(\sum_k \mathcal{M}_k^i \nu_k\right)\left(\sum_h \mathcal{M}_h^j \nu_h\right) = \omega_\mathrm{Q}^2 \sum_k^N \mathcal{M}_k^i \mathcal{M}_k^j + \omega_\mathrm{c}^2 \nu^2 \left(\sum_k \mathcal{M}_k^i\right)\left(\sum_h \mathcal{M}_h^j\right). \tag{S116}$$

A. Uniform light-matter coupling and spectator mode - Rxn interactions

If we define the effective mode as the collective bright mode $Q_B = \sum_i \mathcal{M}_i^1 \mathcal{Q}_i = \frac{1}{\kappa}\sum_i \nu_i \mathcal{Q}_i$, taking $\kappa = \sqrt{\sum_i \nu_i^2}$ and $\mathcal{M}_i^1 = \nu_i/\kappa$. We also define the set of dark in a similar way, taking $\tilde{Q}_k = \sum_i \mathcal{M}_i^k \mathcal{Q}_i$ for $2 \leq k \leq N$, as these must be orthogonal to the $\mathcal{Q}_B$ mode, the coefficients are subject to $\sum_i \mathcal{M}_i^k \mathcal{M}_i^1 = 0$. Under these conditions, and taking $\omega_i = \omega_\mathrm{Q}$ as well as $\nu_i = \nu$, the Hessian is

written as

$$
\boldsymbol{U}_{\mathbf{R}}\boldsymbol{\mathcal{H}}\boldsymbol{U}_{\mathbf{R}}^{\dagger} = \begin{bmatrix} \omega_0^2 + \sum_j \frac{\mathcal{C}_j^2}{\omega_j^2} & \sum_i \mathcal{M}_i^1\mathcal{C}_i & \sum_i \mathcal{M}_i^2\mathcal{C}_i & \cdots & \sum_i \mathcal{M}_i^N\mathcal{C}_i & 0 \\ \sum_i \mathcal{M}_i^1\mathcal{C}_i & \omega_{\mathrm{Q}}^2 + \omega_{\mathrm{c}}^2\kappa^2 & 0 & \cdots & 0 & \omega_{\mathrm{c}}^2\kappa \\ \sum_i \mathcal{M}_i^2\mathcal{C}_i & 0 & \omega_{\mathrm{Q}}^2 & \cdots & 0 & 0 \\ \vdots & \vdots & \vdots & \ddots & \vdots & \vdots \\ \sum_i \mathcal{M}_i^N\mathcal{C}_i & 0 & 0 & \cdots & \omega_{\mathrm{Q}}^2 & 0 \\ 0 & \omega_{\mathrm{c}}^2\kappa & 0 & \cdots & 0 & \omega_{\mathrm{c}}^2 \end{bmatrix}. \tag{S117}
$$

Finally, if consider the mode "Quasi-Bright" $\mathcal{Q}_{\mathrm{QB}} = \frac{1}{\kappa'}\sum_{j=2}^{N}(\sum_i \mathcal{M}_i^j\mathcal{C}_i)\tilde{\mathcal{Q}}_j$, with $\kappa' = \sqrt{\sum_j(\sum_i \mathcal{M}_i^j\mathcal{C}_i)^2}$ it is possible to further separate the modes, where $\mathcal{Q}_{\mathrm{B}}$ directly couple to both R_0 and q_{c}, and $\mathcal{Q}_{\mathrm{QB}}$ only couple to R_0,

$$
\begin{bmatrix} \ddot{R}_0 \\ \ddot{\mathcal{Q}}_{\mathrm{B}} \\ \ddot{\mathcal{Q}}_{\mathrm{QB}} \\ \ddot{q}_{\mathrm{c}} \end{bmatrix} = \begin{bmatrix} \omega_0^2 + \sum_j \frac{\mathcal{C}_j^2}{\omega_j^2} & \frac{1}{\sqrt{N}}\sum_i \mathcal{C}_i & \kappa' & 0 \\ \frac{1}{\sqrt{N}}\sum_i \mathcal{C}_i & \omega_{\mathrm{Q}}^2 + \omega_{\mathrm{c}}^2\kappa^2 & 0 & \omega_{\mathrm{c}}^2\nu\sqrt{N} \\ \kappa' & 0 & \omega_{\mathrm{Q}}^2 & 0 \\ 0 & \omega_{\mathrm{c}}^2\nu\sqrt{N} & 0 & \omega_{\mathrm{c}}^2 \end{bmatrix} \begin{bmatrix} R_0 \\ \mathcal{Q}_{\mathrm{B}} \\ \mathcal{Q}_{\mathrm{QB}} \\ q_{\mathrm{c}} \end{bmatrix}. \tag{S118}
$$

Here, both the bright and dark states couple to the reaction coordinate and the rest of the $N-2$ dark modes are decoupled. These QB modes have the original frequency $\omega_{\mathrm{Q}}$and show up in Fig. S8 and Fig. S9a.

In the case of $\mathcal{C}_i = \mathcal{C}$, $\kappa' = 0$, the "Quasi-Bright" modes are completely decoupled from the reaction coordinate (as in Eq. S64), leading to the following Hessian

$$
\begin{bmatrix} \ddot{R}_0 \\ \ddot{\mathcal{Q}}_{\mathrm{B}} \\ \ddot{q}_{\mathrm{c}} \end{bmatrix} = \begin{bmatrix} \omega_0^2 + N\frac{\mathcal{C}^2}{\omega_{\mathrm{Q}}^2} & \sqrt{N}\mathcal{C} & 0 \\ \sqrt{N}\mathcal{C} & \omega_{\mathrm{Q}}^2 + N\omega_{\mathrm{c}}^2\nu & \omega_{\mathrm{c}}^2\nu\sqrt{N} \\ 0 & \omega_{\mathrm{c}}^2\nu\sqrt{N} & \omega_{\mathrm{c}}^2 \end{bmatrix} \begin{bmatrix} R_0 \\ \mathcal{Q}_{\mathrm{B}} \\ q_{\mathrm{c}} \end{bmatrix}. \tag{S119}
$$

B. $\mathcal{Q}_j - R_0$ coupling disorder

We define an effective mode

$$\tilde{\mathcal{Q}}_1 = \frac{1}{\sqrt{\sum_i \mathcal{C}_i^2}} \sum_i \mathcal{M}_i^1 \mathcal{Q}_i = \frac{1}{\sqrt{\sum_i \mathcal{C}_i^2}} \sum_i \mathcal{C}_i \mathcal{Q}_i. \tag{S120}$$

Taking $\mathcal{M}_i^1 = \mathcal{C}_i / \sqrt{\sum_i \mathcal{C}_i^2}$, $\nu_i = \nu$ and $\mathcal{C}_j = \mathcal{C}$ leads to

$$\boldsymbol{U}_{\mathbf{R}} \boldsymbol{\mathcal{H}} \boldsymbol{U}_{\mathbf{R}}^{\dagger} = \begin{bmatrix} \omega_0^2 + \sum_j \frac{\mathcal{C}_j^2}{\omega_j^2} & \sqrt{\sum_i \mathcal{C}_i^2} & 0 & \cdots & 0 & 0 \\ \sqrt{\sum_i \mathcal{C}_i^2} & \omega_{\mathrm{Q}}^2 + \omega_{\mathrm{c}}^2 \nu^2 \frac{(\sum_i \mathcal{C}_i)^2}{\sum_j \mathcal{C}_j^2} & d_{12} & \cdots & d_{1N} & \omega_{\mathrm{c}}^2 \nu \frac{\sum_i \mathcal{C}_i}{\sqrt{\sum_j \mathcal{C}_j}} \\ 0 & d_{21} & \Omega_2^2 & \cdots & d_{2N} & 0 \\ \vdots & \vdots & \vdots & \ddots & \vdots & \vdots \\ 0 & d_{N1} & d_{N2} & \cdots & \Omega_N^2 & 0 \\ 0 & \omega_{\mathrm{c}}^2 \nu \frac{\sum_i \mathcal{C}_i}{\sqrt{\sum_j \mathcal{C}_j}} & \omega_{\mathrm{c}}^2 \nu \sum_i \mathcal{M}_i^2 & \cdots & \omega_{\mathrm{c}}^2 \nu \sum_i \mathcal{M}_i^N & \omega_{\mathrm{c}}^2 \end{bmatrix}. \tag{S121}$$

In this way, there is an effective mode that directly couples to the reaction coordinate while the influence of the rest of the modes is incorporated by coupling to the effective one. In the same spirit of Eq. S75, we consider only the first mode that is directly coupled to the reaction coordinate as follows

$$\begin{bmatrix} \ddot{R}_0 \\ \ddot{\tilde{\mathcal{Q}}}_1 \\ \ddot{q}_{\mathrm{c}} \end{bmatrix} = \begin{bmatrix} \omega_0^2 + \sum_j \frac{\mathcal{C}_j^2}{\omega_j^2} & \sqrt{\sum_i \mathcal{C}_i^2} & 0 \\ \sqrt{\sum_i \mathcal{C}_i^2} & \omega_{\mathrm{Q}}^2 + \omega_{\mathrm{c}}^2 \nu^2 \frac{(\sum_i \mathcal{C}_i)^2}{\sum_j \mathcal{C}_j^2} & \omega_{\mathrm{c}}^2 \nu \frac{\sum_i \mathcal{C}_i}{\sqrt{\sum_j \mathcal{C}_j}} \\ 0 & \omega_{\mathrm{c}}^2 \nu \frac{\sum_i \mathcal{C}_i}{\sqrt{\sum_j \mathcal{C}_j}} & \omega_{\mathrm{c}}^2 \end{bmatrix} \begin{bmatrix} R_0 \\ \tilde{\mathcal{Q}}_1 \\ q_{\mathrm{c}} \end{bmatrix}. \tag{S122}$$

However, the rest of the dark modes' effect will show up due to their coupling to $\mathcal{Q}_1$ as the magnitude of the disorder increases, as indicated in Fig. S9a.

Supporting Information 8: Cavity Lifetime Dependence

We evaluate the cavity lifetime dependence of the VSC rate constant at a $\Omega_{\mathrm{R}} = 100\ \mathrm{cm}^{-1}$ and the resonance condition $\omega_{\mathrm{c}} = \omega_{\mathrm{Q}}$. The results are obtained from HEOM (dots) and FGR rate theory (solid line), presented in Fig. S12. The strong coupling condition requires $\Omega_{\mathrm{R}} \gg \frac{1}{2}\tau_{\mathrm{c}}^{-1} + \lambda_{\mathrm{Q}}/\gamma_{\mathrm{Q}}$ which is met at around $\tau_{\mathrm{c}} > 250$ fs, corresponding to $\tau_{\mathrm{c}}^{-1} \approx 20\ \mathrm{cm}^{-1}$. The FGR theory agrees well with the numerically exact results. The VSC suppression starts to saturate for $\tau_{\mathrm{c}} > 350$ fs.

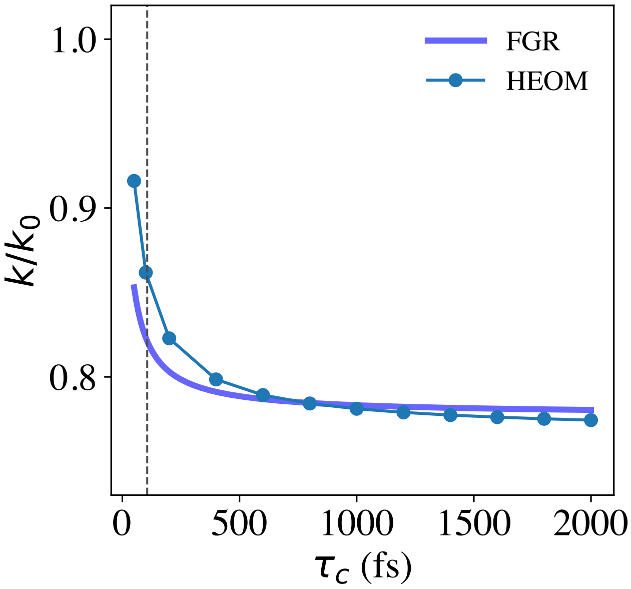

Figure S12: Cavity lifetime dependence of the rate. The dashed line indicates $\tau_{\mathrm{c}} \approx 50$ fs. The typical cavity lifetime is $\tau_{\mathrm{c}} = 200$ fs ~ 1 ps in VSC experiments.

Supporting Information 9: Many Modes Hamiltonian and the Normal Incidence Condition for VSC effects

Here, we explore the resonance condition at the normal incidence by generalizing the Hamiltonian into a single molecule coupled to many cavity modes.

A. The many-mode Hamiltonian

Under the long-wavelength approximation, the many-mode Hamiltonian is expressed as[40]

$$\hat{H} = \hat{H}_{\mathrm{M}} + \hat{H}_{\mathcal{Q}} + \hat{H}_{\nu} + \hat{H}_{\mathrm{loss}} + \frac{1}{2}\sum_{\mathbf{k}}\left[\hat{p}_{\mathbf{k}}^2 + \omega_{\mathbf{k}}^2\Big(\hat{q}_{\mathbf{k}} + \frac{\lambda_{\mathrm{c}}}{\omega_{\mathbf{k}}}\cdot\hat{\mathcal{S}}_{\mathbf{k}}\Big)^2\right], \tag{S123}$$

where $\hat{\mathcal{S}}_{\mathbf{k}} = \sum_j \hat{\mathcal{Q}}_j \cos\varphi_{j,\mathbf{k}}$, and $\varphi_{j,\mathbf{k}}$ is the angle between the spectator mode $\hat{\mathcal{Q}}_j$ dipole orientation $\vec{\mathbf{e}}_j$ and the $\mathbf{k}$-th cavity mode field polarization direction $\vec{\mathbf{e}}_{\mathbf{k}}$, such that $\vec{\mathbf{e}}_j\cdot\vec{\mathbf{e}}_{\mathbf{k}} = \cos\varphi_{j,\mathbf{k}}$. Further, the photonic wavevector $\mathbf{k}$ (also the field propagation direction) has two components, one perpendicular to the cavity mirror $k_\perp$, and the other coplanar with the cavity $k_\parallel$. The dispersion relation of the FP cavity is

$$\omega_{\mathbf{k}}(k_\parallel) = \frac{c}{n_{\mathrm{c}}}\sqrt{k_\perp^2 + k_\parallel^2} = \frac{ck_\perp}{n_{\mathrm{c}}}\sqrt{1+\tan^2\theta}, \tag{S124}$$

where c is the speed of light in vacuum, n_{c} is the refractive index of the cavity, c/n_{c} is the speed of the light inside the cavity, and θ is the incident angle, which is the angle of the photonic mode wavevector $\mathbf{k}$ relative to the norm direction of the mirrors. For simplicity, we explicitly drop n_{c} for the later discussions (because for most of the experiments,$n_{\mathrm{c}} \approx 1$). When $k_\parallel = 0$ (or $\theta = 0$), the photon mode frequency is

$$\omega_{\mathrm{c}} \equiv \omega_{\mathbf{k}}(k_\parallel = 0) = ck_\perp. \tag{S125}$$

In Eq. S123, we had assumed the dipole of the molecule is parallel with the mirrors, $\lambda_{\mathrm{c}} = \sqrt{1/(\epsilon_0\mathcal{V})}$, $\phi_{\mathbf{k}}$ is the angle between the dipole and the field, and we only consider the TE modes of the field. See Ref. 40 Supplementary Material for a detailed discussion. Furthermore, $\hat{q}_{\mathbf{k}} = \sqrt{\hbar/(2\omega_{\mathbf{k}})}(\hat{a}_{\mathbf{k}}^\dagger + \hat{a}_{\mathbf{k}})$ and $\hat{p}_{\mathbf{k}} = i\sqrt{\hbar\omega_{\mathbf{k}}/2}(\hat{a}_{\mathbf{k}}^\dagger - \hat{a}_{\mathbf{k}})$, $\hat{a}_{\mathbf{k}}$ and $\hat{a}_{\mathbf{k}}^\dagger$ are the photonic field annihilation and creation operators for mode $\mathbf{k}$, respectively. By using the cavity dispersion relation in Eq. S124, one has the photonic

density of states (DOS) for a 1D FP cavity as follows,[40]

$$g_{1\mathrm{D}}(\omega) = \frac{2}{c\Delta k_{\parallel}} \cdot \frac{\omega}{\sqrt{\omega^2 - \omega_{\mathrm{c}}^2}} \cdot \Theta(\omega - \omega_{\mathrm{c}}), \tag{S126}$$

where $\Theta(\omega - \omega_{\mathrm{c}})$ is the Heaviside step function, $\Delta k_{\parallel}$ is the spacing of the in-plane wavevector $k_{\parallel}$ (or the k-space lattice constant). The DOS, $g_{1\mathrm{D}}(\omega)$, in Eq. S126 has a singularity at $\omega = \omega_{\mathrm{c}}$, which is known as (the first type of) the van-Hove-type singularity.[41] This singularity naturally explains the resonance condition at the normal incidence for the cases of a 1D FP cavity,[40] because it will force the dominant contribution of the rate constant to only show up at $\omega - \omega_{\mathrm{c}}$. The DOS for a 2D FP cavity is[40]

$$g_{2\mathrm{D}}(\omega) = \frac{2\pi}{(c\Delta k_{\parallel})^2} \cdot \omega \cdot \Theta(\omega - \omega_{\mathrm{c}}), \tag{S127}$$

which does not have any singularity. The detailed derivations of these two DOS can be found in Ref. 40 Supplementary Material.

Further, $\hat{H}_{\nu}$ in Eq. S123 is the same as Eq. 29 of the main text, and $\hat{H}_{\mathrm{loss}}$ is expressed as follows

$$\hat{H}_{\mathrm{loss}} = \frac{1}{2} \sum_{\mathbf{k},\zeta} \Big[\hat{p}_{\mathbf{k},\zeta}^2 + \omega_{\mathbf{k},\zeta}^2 \Big(\hat{x}_{\mathbf{k},\zeta} - \frac{c_{\mathbf{k},\zeta}}{\omega_{\mathbf{k},\zeta}^2} \hat{q}_{\mathbf{k}}\Big)^2\Big], \tag{S128}$$

where $\{\hat{x}_{\mathbf{k},\zeta}, \hat{p}_{\mathbf{k},\zeta}\}$ are the mass-weighted coordinate and momentum operators of the $\{\mathbf{k}, \zeta\}$-th non-cavity bath mode, respectively, which directly couple to the photon mode coordinate operator $\hat{q}_{\mathbf{k}}$. The $\mathbf{k}$-th photon-loss bath as well as its coupling to the photon mode coordinate operator $\hat{q}_{\mathbf{k}}$ are described by the spectral density function

$$J_{\mathrm{loss}}(\omega, \mathbf{k}) = \frac{\pi}{2} \sum_{\zeta} \frac{c_{\mathbf{k},\zeta}^2}{\omega_{\mathbf{k},\zeta}} \delta(\omega - \omega_{\mathbf{k},\zeta}) = \frac{\omega}{\tau_{\mathrm{c}}} e^{-\omega/\omega_{\mathrm{m}}}, \tag{S129}$$

where $\omega_{\mathbf{k},\zeta}$, $c_{\mathbf{k},\zeta}$ are the oscillator frequencies and coupling coefficients, respectively. Note that for simplicity we had assumed that the cavity loss is homogeneous (*i.e.*, does not depend on $\mathbf{k}$) and strictly Ohmic, where the cutoff frequency $\omega_{\mathrm{m}} \to \infty$, reaching to the Markovian limit. The discussion of the non-Markovian loss can be found in Ref. 5.

B. Effective spectral density with many cavity modes: the most general case

We derive the general expression of the effective spectral density by considering many cavity modes. Following the same derivations procedure provided in Supporting Information 5, Sec. A, it is easy to derive that the effective spectral density $J_{\text{eff}}(\omega)$ has the same general form as expressed in Eq. S55, but with modified $\boldsymbol{\mathcal{M}}$ matrix, expressed as follows,

$$\boldsymbol{\mathcal{M}} = \text{diag}\Big\{\omega_1^2 + P_1(\omega), \omega_2^2 + P_2(\omega), \cdots, \omega_N^2 + P_N(\omega)\Big\} + \boldsymbol{\Psi}(\omega), \tag{S130}$$

where the matrix elements of $\boldsymbol{\Psi}(\omega)$ is expressed as

$$[\boldsymbol{\Psi}]_{jj'}(\omega) = \sum_{\mathbf{k}} \frac{\lambda_{\text{c}}^2 L(\omega)}{\omega_{\mathbf{k}}^2 + L(\omega)} \cos\varphi_{j,\mathbf{k}} \cdot \cos\varphi_{j',\mathbf{k}}, \qquad j, j' = 1, \cdots, N, \tag{S131}$$

and the expression for $L(\omega)$ can be found in Eq. S50b. Nevertheless, $J_{\text{eff}}(\omega)$ with many modes cannot be solved analytically either. One can get numerical solutions easily given the detailed configurations of the molecules and cavity modes.

C. Effective spectral density with many cavity modes: analytic form with approximations

To obtain analytic results, we consider the case with uniform spectator mode frequencies and adopt the same approximations made in Supporting Information 5, Sec. C, and D. As a result, $\omega_j \to \omega_{\text{Q}}$, and all the matrix elements of $\boldsymbol{\Psi}(\omega)$ become identical, reading as

$$[\boldsymbol{\Psi}]_{jj'}(\omega) = \sum_{\mathbf{k}} \frac{\lambda_{\text{c}}^2 \chi^2 L(\omega)}{\omega_{\mathbf{k}}^2 + L(\omega)} \cos^2\phi_{\mathbf{k}} \equiv \psi(\omega), \tag{S132}$$

where $\phi_{\mathbf{k}}$ is the angle between the field polarization and the projection of the dipole on the $k_{\|}$ plane.[40] One obtains the analytic form of $J_{\text{eff}}(\omega)$ expressed as follows,

$$J_{\text{eff}}^{\text{D}}(\omega) = \frac{\Lambda \cdot \omega_{\text{Q}}^2 \cdot \omega \Gamma_{\text{Q}}^{\text{D}}(\omega)}{[\omega_{\text{Q}}^2 - \omega^2 + \tilde{\mathcal{R}}^{\text{D}}(\omega)]^2 + [\omega \Gamma_{\text{Q}}^{\text{D}}(\omega)]^2}, \tag{S133}$$

where $\mathrm{D} = 1, 2$ denotes the dimension. Eq. S133 is of the same form as Eq. S62, but with a modified expressions of $\Gamma_{\mathrm{Q}}(\omega)$ and $\tilde{\mathcal{R}}(\omega)$ that each sums over all modes, expressed as follows

$$\Gamma_{\mathrm{Q}}^{\mathrm{D}}(\omega) = \frac{2\lambda_{\mathrm{Q}}}{\gamma_{\mathrm{Q}}} + \sum_{\mathbf{k}} \frac{N\chi^2 \cdot \lambda_{\mathrm{c}}^2 \cos^2\phi_{\mathbf{k}} \omega_{\mathbf{k}}^2 \tau_{\mathrm{c}}^{-1}}{\left(\omega_{\mathbf{k}}^2 - \omega^2\right)^2 + \omega^2\tau_{\mathrm{c}}^{-2}}, \tag{S134a}$$

$$\tilde{\mathcal{R}}^{\mathrm{D}}(\omega) = \sum_{\mathbf{k}} \frac{N\chi^2 \cdot \lambda_{\mathrm{c}}^2 \cos^2\phi_{\mathbf{k}} \omega^2}{\left(\omega_{\mathbf{k}}^2 - \omega^2\right)^2 + \omega^2\tau_{\mathrm{c}}^{-2}} \cdot (\omega^2 - \omega_{\mathbf{k}}^2 + \tau_{\mathrm{c}}^{-2}), \tag{S134b}$$

where $\lambda_{\mathrm{c}} = \sqrt{1/(\epsilon_0 \mathcal{V})}$ is the cavity frequency independent coupling strength, $\phi_{\mathbf{k}}$ is the angle between the field polarization and the dipole projected on the plane, which is zero for a 1D FP cavity and varies from 0 to 2π for a 2D FP cavity.

Next, we use the photonic mode DOS introduced in the previous section (Eq. S126 and Eq. S127) to replace the sum over modes as an integral $\sum_{\mathbf{k}} f(\mathbf{k}) \to \int d\omega\ g_{\mathrm{D}}(\omega) f(\omega)$, where $g_{\mathrm{D}}(\omega)$ is the DOS for the cavity modes. Nevertheless, $g_{\mathrm{2D}}(\omega)$ is unbounded, which leads to ultraviolet divergence if one performs the integral directly. To solve this problem, we keep the total reorganization energy of light-matter interaction fixed.[40] For thermally driven processes, we further introduce $\mathcal{P}_{\mathbf{k}} = e^{-\beta\hbar\omega_{\mathbf{k}}}/\mathcal{Z}$ which represents the thermal weighting factor for accessing the cavity mode $\omega_{\mathbf{k}}$, and $\mathcal{Z}$ is the partition function such that $\sum_{\mathbf{k}} \mathcal{P}_{\mathbf{k}} = 1$. Eq. S134 is modified as follows

$$\Gamma_{\mathrm{Q}}^{\mathrm{D}}(\omega) = \frac{2\lambda_{\mathrm{Q}}}{\gamma_{\mathrm{Q}}} + \sum_{\mathbf{k}} \mathcal{P}_{\mathbf{k}} \cdot \frac{N\chi^2 \cdot \lambda_{\mathrm{c}}^2 \cos^2\phi_{\mathbf{k}} \omega_{\mathbf{k}}^2 \tau_{\mathrm{c}}^{-1}}{\left(\omega_{\mathbf{k}}^2 - \omega^2\right)^2 + \omega^2\tau_{\mathrm{c}}^{-2}}, \tag{S135a}$$

$$\tilde{\mathcal{R}}^{\mathrm{D}}(\omega) = \sum_{\mathbf{k}} \mathcal{P}_{\mathbf{k}} \cdot \frac{N\chi^2 \cdot \lambda_{\mathrm{c}}^2 \cos^2\phi_{\mathbf{k}} \omega^2}{\left(\omega_{\mathbf{k}}^2 - \omega^2\right)^2 + \omega^2\tau_{\mathrm{c}}^{-2}} \cdot (\omega^2 - \omega_{\mathbf{k}}^2 + \tau_{\mathrm{c}}^{-2}), \tag{S135b}$$

As a result, the total reorganization energy of light-matter interaction fixed.[40] One can also associate the $\mathcal{P}_{\mathbf{k}}$ factor (or $\mathcal{P}(\omega) = e^{-\beta\hbar\omega}/\mathcal{Z}$) with the DOS $g_{\mathrm{D}}(\omega)$ and view $g_{\mathrm{D}}(\omega)\mathcal{P}(\omega)$ as the thermal DOS, which is bounded and normalized (since $\mathcal{Z} = \int d\omega\ g_{\mathrm{D}}(\omega)\mathcal{P}(\omega)$). On the other hand, the $\mathcal{P}_{\mathbf{k}}$ factor also accounts for **k**-dependent light-matter coupling.

For a 1D FP cavity, due to the van-Hove-type singularity[41] in the 1D photonic DOS at $\omega = \omega_{\mathrm{c}}$, $g_{\mathrm{1D}}(\omega)$ forces the integral $\int d\omega\ g_{\mathrm{D}}(\omega) f(\omega)$ to take the value at $\omega = \omega_{\mathrm{c}}$ (which is the dominant contribution to the integral), and Eq. S135a reduces back to Eq. S64a, which means the resulting $J_{\mathrm{eff}}(\omega)$ is almost the same as the single mode case. Specifically, under the continuous limit, one has

$$
\begin{aligned}
\Gamma_{\mathrm{Q}}^{1\mathrm{D}}(\omega) &= \frac{2\lambda_{\mathrm{Q}}}{\gamma_{\mathrm{Q}}} + \sum_{\mathbf{k}} \mathcal{P}_{\mathbf{k}} \cdot \frac{N\chi^2 \cdot \lambda_{\mathrm{c}}^2 \omega_{\mathbf{k}}^2 \tau_{\mathrm{c}}^{-1}}{\left(\omega_{\mathbf{k}}^2 - \omega^2\right)^2 + \omega^2 \tau_{\mathrm{c}}^{-2}} \\
&= \frac{2\lambda_{\mathrm{Q}}}{\gamma_{\mathrm{Q}}} + \int d\omega \; g_{1\mathrm{D}}(\omega) \mathcal{P}(\omega) \cdot \frac{N\chi^2 \cdot \lambda_{\mathrm{c}}^2 \omega^2 \tau_{\mathrm{c}}^{-1}}{\left(\omega^2 - \omega^2\right)^2 + \omega^2 \tau_{\mathrm{c}}^{-2}} \\
&\approx \frac{2\lambda_{\mathrm{Q}}}{\gamma_{\mathrm{Q}}} + \frac{N\chi^2 \cdot \lambda_{\mathrm{c}}^2 \omega_{\mathrm{c}}^2 \tau_{\mathrm{c}}^{-1}}{\left(\omega_{\mathrm{c}}^2 - \omega^2\right)^2 + \omega^2 \tau_{\mathrm{c}}^{-2}}, \qquad \text{(S136a)} \\
\tilde{\mathcal{R}}^{1\mathrm{D}}(\omega) &= \sum_{\mathbf{k}} \mathcal{P}_{\mathbf{k}} \cdot \frac{N\chi^2 \cdot \lambda_{\mathrm{c}}^2 \omega^2}{\left(\omega_{\mathbf{k}}^2 - \omega^2\right)^2 + \omega^2 \tau_{\mathrm{c}}^{-2}} \cdot \left(\omega^2 - \omega_{\mathbf{k}}^2 + \tau_{\mathrm{c}}^{-2}\right) \\
&= \int d\omega \; g_{1\mathrm{D}}(\omega) \mathcal{P}(\omega) \cdot \frac{N\chi^2 \cdot \lambda_{\mathrm{c}}^2 \omega^2}{\left(\omega^2 - \omega^2\right)^2 + \omega^2 \tau_{\mathrm{c}}^{-2}} \cdot \left(\omega^2 - \omega^2 + \tau_{\mathrm{c}}^{-2}\right) \\
&\approx \frac{N\chi^2 \cdot \lambda_{\mathrm{c}}^2 \omega^2}{\left(\omega_{\mathrm{c}}^2 - \omega^2\right)^2 + \omega^2 \tau_{\mathrm{c}}^{-2}} \cdot \left(\omega^2 - \omega_{\mathrm{c}}^2 + \tau_{\mathrm{c}}^{-2}\right), \qquad \text{(S136b)}
\end{aligned}
$$

where $\mathcal{P}(\omega) = e^{-\beta\hbar\omega}/\mathcal{Z}$, and we have explicitly used $\cos\phi_{\mathbf{k}} = 1$, and the 1D DOS is defined and evaluated as follows

$$
g_{1\mathrm{D}}(\omega) = \int \frac{dk}{\Delta k_{\parallel}} \; \delta(\omega - \omega_{\mathbf{k}}) = \frac{2}{c\Delta k_{\parallel}} \cdot \frac{\omega}{\sqrt{\omega^2 - \omega_{\mathrm{c}}^2}} \cdot \Theta(\omega - \omega_{\mathrm{c}}). \qquad \text{(S137)}
$$

By substituting $\lambda_{\mathrm{c}}^2 = 2\eta_{\mathrm{c}}^2 \hbar\omega_{\mathrm{c}}$, Eqs. 9-12 (the single mode case) of the main text are recovered. The effective spectral density $J_{\mathrm{eff}}^{1\mathrm{D}}(\omega)$ is identical as the Eq. 9 of the main text. As a result, inside a 1D FP cavity, VSC modification occurs only at the normal incidence $\omega_{\mathrm{c}} = \omega_0$. One can also numerically evaluate the integral in Eq. S136, which is the solid line presented in Fig. 6a of the main text, and visually identical to the analytic results (dashed line) in Fig. 6a.

On the other hand, all of the known VSC experiments[28,36,42,43] have been performed in 2D FP cavities. To explain the normal incidence conditions, our previous work[40] suggests that thermal photons with a finite momentum in the in-plane direction will leave the effective mode area characterized by the following effective lifetime[40]

$$
\tau_{\parallel}(k_{\parallel}) = \frac{\mathcal{D}}{c \cdot \sin\theta} = \frac{\mathcal{D}}{c} \cdot \frac{\omega_{\mathbf{k}}}{\sqrt{\omega_{\mathbf{k}}^2 - \omega_{\mathrm{c}}^2}}, \qquad \text{(S138)}
$$

where $\mathcal{D}$ characterizes the spatial extent of a given mode (along the $k_{\parallel}$ direction). Using the

experimental molecular density and the effective number of molecules coupled to a given mode,[44] one can estimate[40] that $\mathcal{D} \approx 10^{-1} \sim 100\ \mu$m. Note that $\tau_{\parallel}$ is different than τ_c that characterizes the loss along the norm direction (perpendicular to the mirror). Taking into account of $\tau_{\parallel}$ in Eq. S138, the probability for occupying $\omega_\mathbf{k}$ mode $\mathcal{P}_\mathbf{k}$ (that appears in Eq. S134) will be modified as

$$\mathcal{P}_\mathrm{eff}(\omega_\mathbf{k}) = \frac{1}{\mathcal{Z}_\mathrm{eff}} \frac{\tau_\mathrm{c}^{-1} e^{-\beta\hbar\omega_\mathbf{k}}}{\tau_\mathrm{c}^{-1} + \tau_{\parallel}^{-1}}, \tag{S139}$$

where $\mathcal{Z}_\mathrm{eff}$ is the normalization factor (effective partition function) for $\mathcal{P}_\mathrm{eff}$. Note that the correction factor $\tau_\mathrm{c}^{-1}/(\tau_\mathrm{c}^{-1} + \tau_{\parallel}^{-1})$ should also be applied to the 1D FP cavity but will not introduce any visible difference, due to the van-Hove singularity in the 1D DOS that dominates the entire integral,[40] forcing $\mathcal{P}_\mathrm{eff}(\omega_\mathbf{k}) \to \mathcal{P}_\mathbf{k}$ (as $\tau_{\parallel} \to \infty$ when $k_{\parallel} = 0$). To simplify our theoretical analysis, we assume that the dipolar orientation disorder can be viewed as a static disorder, and use isotropic average $\cos^2\phi_\mathbf{k} \to \langle \cos^2\phi_\mathbf{k} \rangle = 1/2$ in both Eq. S134a and Eq. S134b as follows

$$\begin{aligned}
\Gamma_\mathrm{Q}^\mathrm{2D}(\omega) &= \frac{2\lambda_\mathrm{Q}}{\gamma_\mathrm{Q}} + \sum_\mathbf{k} \frac{1}{2} \mathcal{P}_\mathrm{eff}(\omega_\mathbf{k}) \cdot \frac{N\chi^2 \cdot \lambda_\mathrm{c}^2 \omega_\mathbf{k}^2 \tau_\mathrm{c}^{-1}}{\left(\omega_\mathbf{k}^2 - \omega^2\right)^2 + \omega^2 \tau_\mathrm{c}^{-2}} \\
&= \frac{2\lambda_\mathrm{Q}}{\gamma_\mathrm{Q}} + \int d\tilde{\omega}\ \frac{1}{2} g_\mathrm{2D}(\tilde{\omega}) \mathcal{P}_\mathrm{eff}(\tilde{\omega}) \cdot \frac{N\chi^2 \cdot \lambda_\mathrm{c}^2 \tilde{\omega}^2 \tau_\mathrm{c}^{-1}}{\left(\tilde{\omega}^2 - \omega^2\right)^2 + \omega^2 \tau_\mathrm{c}^{-2}} \\
&= \frac{2\lambda_\mathrm{Q}}{\gamma_\mathrm{Q}} + \int_{\omega_\mathrm{c}}^{\infty} d\tilde{\omega} \frac{\mathcal{F}(\tilde{\omega}) \cdot N\chi^2 \cdot \lambda_\mathrm{c}^2 \tilde{\omega}^2 \tau_\mathrm{c}^{-1}}{\left(\tilde{\omega}^2 - \omega^2\right)^2 + \omega^2 \tau_\mathrm{c}^{-2}},
\end{aligned} \tag{S140a}$$

$$\begin{aligned}
\tilde{\mathcal{R}}^\mathrm{2D}(\omega) &= \sum_\mathbf{k} \frac{1}{2} \mathcal{P}_\mathbf{k} \cdot \frac{N\chi^2 \cdot \lambda_\mathrm{c}^2 \omega^2}{\left(\omega_\mathbf{k}^2 - \omega^2\right)^2 + \omega^2 \tau_\mathrm{c}^{-2}} \cdot (\omega^2 - \omega_\mathbf{k}^2 + \tau_\mathrm{c}^{-2}) \\
&= \int d\tilde{\omega}\ \frac{1}{2} g_\mathrm{2D}(\tilde{\omega}) \mathcal{P}_\mathrm{eff}(\tilde{\omega}) \cdot \frac{N\chi^2 \cdot \lambda_\mathrm{c}^2 \omega^2}{\left(\tilde{\omega}^2 - \omega^2\right)^2 + \omega^2 \tau_\mathrm{c}^{-2}} \cdot (\omega^2 - \tilde{\omega}^2 + \tau_\mathrm{c}^{-2}) \\
&= \int_{\omega_\mathrm{c}}^{\infty} d\tilde{\omega}\ \frac{\mathcal{F}(\tilde{\omega}) \cdot N\chi^2 \cdot \lambda_\mathrm{c}^2 \omega^2}{\left(\tilde{\omega}^2 - \omega^2\right)^2 + \omega^2 \tau_\mathrm{c}^{-2}} \cdot (\omega^2 - \tilde{\omega}^2 + \tau_\mathrm{c}^{-2}),
\end{aligned} \tag{S140b}$$

where the weighting factor $\mathcal{F}(\omega) \equiv \frac{1}{2} g_\mathrm{2D}(\omega) \mathcal{P}_\mathrm{eff}(\omega)$ is expressed as

$$\mathcal{F}(\omega) = \frac{1}{\mathcal{Z}_\mathrm{eff}} \frac{\pi}{(c\Delta k_{\parallel})^2} \cdot \frac{\tau_\mathrm{c}^{-1} \omega e^{-\beta\hbar\omega}}{\tau_\mathrm{c}^{-1} + \tau_{\parallel}^{-1}(\omega)}, \tag{S141}$$

with the 2D photonic DOS defined and evaluated as follows,

$$g_{\rm 2D}(\omega) = \int \frac{dk^2}{(\Delta k_{\parallel})^2}\ \delta(\omega - \omega_{\mathbf{k}}) = \frac{2\pi}{(c\Delta k_{\parallel})^2} \cdot \omega \cdot \Theta(\omega - \omega_{\rm c}), \tag{S142}$$

and $\tau_{\parallel}(\omega) = \omega\mathcal{D}/\big[c\sqrt{\omega^2 - \omega_{\rm c}^2}\ \big]$ (c.f. Eq. S138). The cavity frequency $\omega_{\rm c}$ at the normal incidence ($k_{\parallel} = 0$) implicitly shows up in the expressions of $\Gamma_{\rm Q}^{\rm 2D}(\omega)$ and $\tilde{\mathcal{R}}^{\rm 2D}(\omega)$ (see Eq. S140a and Eq. S140b) as the lower bound of the integral, due to the photonic DOS only has a finite value with $\omega_{\mathbf{k}} \geq \omega_{\rm c}$ (for $|k_{\parallel}| \geq 0$). This is different than the single-mode case where $\omega_{\rm c}$ explicitly shows up in the expression of $\Gamma_{\rm Q}(\omega)$ (Eq. S64a) and $\tilde{\mathcal{R}}(\omega)$ (Eq. S64b), or for the 1D FP cavity case where the van-Hove-type singularity in photonic DOS forces the integral to take value only at $\omega = \omega_{\rm c}$.

With Eqs. S140a-S140b, one has the effective spectral density inside a 2D cavity as follows,

$$J_{\rm eff}^{\rm 2D}(\omega) = \frac{\Lambda\omega_{\rm Q}^2 \cdot \omega\Gamma_{\rm Q}^{\rm 2D}(\omega)}{[\omega_{\rm Q}^2 - \omega^2 + \tilde{\mathcal{R}}^{\rm 2D}(\omega)]^2 + [\omega\Gamma_{\rm Q}^{\rm 2D}(\omega)]^2}, \tag{S143}$$

and the FGR rate constant is expressed as

$$\begin{aligned} k_{\rm VSC}^{\rm 2D} &= 2|\Delta_x|^2 \int_0^{\infty} d\omega\ J_{\rm eff}^{\rm 2D}(\omega) \cdot n(\omega) \cdot \mathcal{A}_0(\omega - \omega_0) \\ &= 2|\Delta_x|^2 \int_0^{\infty} d\omega\ \frac{\Lambda\omega_{\rm Q}^2 \cdot \omega\Gamma_{\rm Q}^{\rm 2D}(\omega) \cdot \mathcal{A}_0(\omega - \omega_0) \cdot n(\omega)}{[\omega_{\rm Q}^2 - \omega^2 + \tilde{\mathcal{R}}^{\rm 2D}(\omega)]^2 + [\omega\Gamma_{\rm Q}^{\rm 2D}(\omega)]^2}, \end{aligned} \tag{S144}$$

which is just Eq. 22 of the main text. And $\Gamma_{\rm Q}^{\rm 2D}(\omega)$ and $\mathcal{R}^{\rm 2D}(\omega)$ are expressed in Eqs. S140a-S140b (recovering Eq. 23 of the main text).

D. Numerical details of evaluating $k_{\rm VSC}^{\rm 2D}$

Here, we choose $\mathcal{D}/c = 3.33$ fs, which corresponds to $\mathcal{D} = 1\ \mu$m for the effective mode diameter. First, we use $\omega_{\rm m} = 5\omega_{\rm c}$ as the cutoff frequency to perform trapezoidal integration scheme of Eqs. S140a-S140b to obtain $\Gamma_{\rm Q}^{\rm 2D}(\omega)$ and $\mathcal{R}^{\rm 2D}(\omega)$, with 4×10^4 grid points, where numerical convergence is carefully checked. Note that in the above calculations, the cutoff frequency $\omega_{\rm m}$ is treated as a convergence parameter, and using $\omega_{\rm m} = 3\omega_{\rm c}$ (with 10^4 grid points) already provides almost converged results. Then, the resulting $\Gamma_{\rm Q}^{\rm 2D}(\omega)$ and $\mathcal{R}^{\rm 2D}(\omega)$ are plug into Eq. S143 to obtain the

effective spectral density inside a 2D cavity, and the VSC rate constant in Eq. S144 is evaluated via Riemann sum using 10^4 grid points within the range of $[0, 3\omega_c]$ for the convolution.

References

(1) Topaler, M.; Makri, N. Quantum rates for a double well coupled to a dissipative bath: Accurate path integral results and comparison with approximate theories. *J. Chem. Phys.* **1994**, *101*, 7500–7519.

(2) Shi, Q.; Zhu, L.; Chen, L. Quantum rate dynamics for proton transfer reaction in a model system: Effect of the rate promoting vibrational mode. *J. Chem. Phys.* **2011**, *135*, 044505.

(3) Colbert, D. T.; Miller, W. H. A novel discrete variable representation for quantum mechanical reactive scattering via the S-matrix Kohn method. *J. Chem. Phys.* **1992**, *96*, 1982–1991.

(4) Lindoy, L. P.; Mandal, A.; Reichman, D. R. Quantum dynamical effects of vibrational strong coupling in chemical reactivity. *Nat. Commun.* **2023**, *14*, 2733.

(5) Ying, W.; Huo, P. Resonance theory and quantum dynamics simulations of vibrational polariton chemistry. *J. Chem. Phys.* **2023**, *159*, 084104.

(6) Garg, A.; Onuchic, J. N.; Ambegaokar, V. Effect of friction on electron transfer in biomolecules. *J. Chem. Phys.* **1985**, *83*, 4491–4503.

(7) Ying, W.; Huo, P. Resonance theory of vibrational strong coupling enhanced polariton chemistry and the role of photonic mode lifetime. *Commun. Mater.* **2024**, *5*, 110.

(8) Mukamel, S. *Principles of Nonlinear Optical Spectroscopy*; Oxford University Press, 1995.

(9) Tanimura, Y. Stochastic Liouville, Langevin, Fokker–Planck, and Master Equation Approaches to Quantum Dissipative Systems. *J. Phys. Soc. Jpn.* **2006**, *75*, 082001.

(10) Xu, R.-X.; Cui, P.; Li, X.-Q.; Mo, Y.; Yan, Y. Exact quantum master equation via the calculus on path integrals. *J. Chem. Phys.* **2005**, *122*, 041103.

(11) Xu, R.-X.; Yan, Y. Dynamics of quantum dissipation systems interacting with bosonic canonical bath: Hierarchical equations of motion approach. *Phys. Rev. E* **2007**, *75*, 031107.

(12) Shao, J. Decoupling quantum dissipation interaction via stochastic fields. *J. Chem. Phys.* **2004**, *120*, 5053–5056.

(13) Yan, Y.; Yang, F.; Liu, Y.; Shao, J. Hierarchical approach based on stochastic decoupling to dissipative systems. *Chem. Phys. Lett.* **2004**, *395*, 216–221.

(14) Yan, Y. Theory of open quantum systems with bath of electrons and phonons and spins: Many-dissipaton density matrixes approach. *J. Chem. Phys.* **2014**, *140*, 054105.

(15) Yan, Y.; Jin, J.; Xu, R.-X.; Zheng, X. Dissipation equation of motion approach to open quantum systems. *Front. Phys.* **2016**, *11*, 110306.

(16) Zhang, H.-D.; Xu, R.-X.; Zheng, X.; Yan, Y. Statistical quasi-particle theory for open quantum systems. *Mol. Phys.* **2018**, *116*, 780–812.

(17) Weiss, U. *Quantum Dissipation Systems*; World Scientific, Singapore, 1993.

(18) Ozaki, T. Continued fraction representation of the Fermi-Dirac function for large-scale electronic structure calculations. *Phys. Rev. B* **2007**, *75*, 035123.

(19) Hu, J.; Xu, R.-X.; Yan, Y. Communication: Padé spectrum decomposition of Fermi function and Bose function. *J. Chem. Phys.* **2010**, *133*, 101106.

(20) Hu, J.; Luo, M.; Jiang, F.; Xu, R.-X.; Yan, Y. Padé spectrum decompositions of quantum distribution functions and optimal hierarchical equations of motion construction for quantum open systems. *J. Chem. Phys.* **2011**, *134*, 244106.

(21) Liu, H.; Zhu, L.; Bai, S.; Shi, Q. Reduced quantum dynamics with arbitrary bath spectral densities: Hierarchical equations of motion based on several different bath decomposition schemes. *J. Chem. Phys.* **2014**, *140*, 134106.

(22) Duan, C.; Wang, Q.; Tang, Z.; Wu, J. The study of an extended hierarchy equation of motion in the spin-boson model: The cutoff function of the sub-Ohmic spectral density. *J. Chem. Phys.* **2017**, *147*, 164112.

(23) Wang, Q.; Gong, Z.; Duan, C.; Tang, Z.; Wu, J. Dynamical scaling in the Ohmic spin-boson model studied by extended hierarchical equations of motion. *J. Chem. Phys.* **2019**, *150*, 084114.

(24) Lambert, N.; Ahmed, S.; Cirio, M.; Nori, F. Modelling the ultra-strongly coupled spin-boson model with unphysical modes. *Nat. Commun.* **2019**, *10*, 3721.

(25) Chen, Z.-H.; Wang, Y.; Zheng, X.; Xu, R.-X.; Yan, Y. Universal time-domain Prony fitting decomposition for optimized hierarchical quantum master equations. *J. Chem. Phys.* **2022**, *156*, 221102.

(26) Tanimura, Y. Reduced hierarchy equations of motion approach with Drude plus Brownian spectral distribution: Probing electron transfer processes by means of two-dimensional correlation spectroscopy. *J. Chem. Phys.* **2012**, *137*, 22A550.

(27) Zhang, H.-D.; Qiao, Q.; Xu, R.-X.; Zheng, X.; Yan, Y. Efficient steady-state solver for hierarchical quantum master equations. *J. Chem. Phys.* **2017**, *147*, 044105.

(28) Thomas, A.; George, J.; Shalabney, A.; Dryzhakov, M.; Varma, S. J.; Moran, J.; Chervy, T.; Zhong, X.; Devaux, E.; Genet, C.; Hutchison, J. A.; Ebbesen, T. W. Ground-State Chemical Reactivity under Vibrational Coupling to the Vacuum Electromagnetic Field. *Angew. Chem. Int. Ed.* **2016**, *55*, 11462–11466.

(29) Wang, H.; Skinner, D. E.; Thoss, M. Calculation of reactive flux correlation functions for systems in a condensed phase environment: A multilayer multiconfiguration time-dependent Hartree approach. *J. Chem. Phys.* **2006**, *125*, 174502.

(30) Hu, D.; Ying, W.; Huo, P. Resonance Enhancement of Vibrational Polariton Chemistry Obtained from the Mixed Quantum-Classical Dynamics Simulations. *J. Phys. Chem. Lett.* **2023**, *14*, 11208–11216.

(31) Menzeleev, A. R.; Ananth, N.; Miller, T. F. Direct simulation of electron transfer using ring polymer molecular dynamics: Comparison with semiclassical instanton theory and exact quantum methods. *The Journal of Chemical Physics* **2011**, *135*, 074106.

(32) Sirjoosingh, A.; Hammes-Schiffer, S. Diabatization Schemes for Generating Charge-Localized Electron–Proton Vibronic States in Proton-Coupled Electron Transfer Systems. *Journal of Chemical Theory and Computation* **2011**, *7*, 2831–2841.

(33) Mandal, A.; Li, X.; Huo, P. Theory of vibrational polariton chemistry in the collective coupling regime. *J. Chem. Phys.* **2022**, *156*, 014101.

(34) Hughes, K. H.; Christ, C. D.; Burghardt, I. Effective-mode representation of non-Markovian dynamics: A hierarchical approximation of the spectral density. I. Application to single surface dynamics. *J. Chem. Phys.* **2009**, *131*, 024109.

(35) Hughes, K. H.; Christ, C. D.; Burghardt, I. Effective-mode representation of non-Markovian dynamics: A hierarchical approximation of the spectral density. II. Application to environment-induced nonadiabatic dynamics. *J. Chem. Phys.* **2009**, *131*, 124108.

(36) Ahn, W.; Triana, J. F.; Recabal, F.; Herrera, F.; Simpkins, B. S. Modification of ground-state chemical reactivity via light–matter coherence in infrared cavities. *Science* **2023**, *380*, 1165–1168.

(37) Schwennicke, K.; Giebink, N. C.; Yuen-Zhou, J. Extracting accurate light–matter couplings from disordered polaritons. *Nanophotonics* **2024**, *13*, 2469–2478.

(38) Chen, H.-T.; Zhou, Z.; Sukharev, M.; Subotnik, J. E.; Nitzan, A. Interplay between disorder and collective coherent response: Superradiance and spectral motional narrowing in the time domain. *Phys. Rev. A.* **2022**, *106*, 053703.

(39) Thomas, A.; Jayachandran, A.; Lethuillier-Karl, L.; Vergauwe, R. M.; Nagarajan, K.; Devaux, E.; Genet, C.; Moran, J.; Ebbesen, T. W. Ground state chemistry under vibrational strong coupling: dependence of thermodynamic parameters on the Rabi splitting energy. *Nanophotonics* **2020**, *9*, 249–255.

(40) Ying, W.; Taylor, M.; Huo, P. Resonance Theory of Vibrational Polariton Chemistry at the Normal Incidence. *Nanophotonics* **2024**, *13*, 2601–2615.

(41) Hove, L. V. The Occurrence of Singularities in the Elastic Frequency Distribution of a Crystal. *Phys. Rev.* **1953**, *89*, 1189–1193.

(42) Hirai, K.; Hutchison, J. A.; Uji-i, H. Recent Progress in Vibropolaritonic Chemistry. *ChemPlusChem* **2020**, *85*, 1981–1988.

(43) Campos-Gonzalez-Angulo, J. A.; Poh, Y. R.; Du, M.; Yuen-Zhou, J. Swinging between shine and shadow: Theoretical advances on thermally activated vibropolaritonic chemistry. *J. Chem. Phys.* **2023**, *158*, 230901.

(44) Shalabney, A.; George, J.; J. Hutchison, G. P.; Genet, C.; Ebbesen, T. Coherent coupling of molecular resonators with a microcavity mode. *Nat. Comm.* **2015**, *6*, 5981.
